# Multi-Omics insights into the molecular mechanisms of trochlear dysplasia: A proteomic and metabolomic study in rats

**DOI:** 10.1371/journal.pone.0325562

**Published:** 2025-08-11

**Authors:** Zhenhui Huo, Huijun Kang, Peishuo Liu, Yanshuo Li, Yingzhen Niu, Kuo Hao, Chongyi Fan, Fei Wang, Wei Lin

**Affiliations:** 1 Department of Orthopaedic Surgery, Third Hospital of Hebei Medical University, Shijiazhuang, Hebei, P.R. China; 2 Department of Orthopaedics, Hebei Province Cangzhou Hospital of Integrated Traditional Chinese Medicine-Western Medicine, Cangzhou, Hebei, P.R. China; 3 Hebei Key Laboratory of Integrated Traditional and Western Medicine in Osteoarthrosis Research, Cangzhou, Hebei, P.R. China; 4 School of Basic Medical Science, Hebei University, Baoding, Hebei, P.R. China; 5 Department of Orthopedics, Aerospace Central Hospital, Beijing, P.R. China; Yarmouk University, JORDAN

## Abstract

**Introduction:**

Trochlear dysplasia (TD) is a skeletal deformity that causes abnormal morphology of the trochlear groove, leading to patellar instability and related joint issues. Despite its clinical importance, the molecular mechanisms behind TD are not well understood. This study aims to explore these mechanisms using an integrated proteomic and metabolomic approach in a rat model of TD.

**Methods:**

A rat model was developed by inducing a flat trochlear groove and increasing the sulcus angle. Validation was performed using gross morphology and micro-CT. Subchondral bone loss was evaluated through micro-CT. Non-targeted metabolomics was applied to identify differential metabolites, and proteomics was conducted to identify altered proteins. Pathway enrichment and interaction analyses were used to interpret the data.

**Results:**

The TD rat model exhibited significant morphological and bone density changes, including notable subchondral bone loss. Metabolomic analysis identified 52 differentially expressed metabolites, with creatine and L-malic acid prominently altered. Proteomic analysis revealed 204 differentially expressed proteins. KEGG analysis highlighted critical pathways such as glycine, serine, and threonine metabolism and the PI3K-Akt signaling pathway. Integrative analysis showed correlations between key metabolites and proteins, providing deeper insights into TD-related molecular changes.

**Conclusions:**

This study integrates proteomic and metabolomic analyses to uncover molecular alterations in a rat model of TD. Significant findings include upregulation of Col3a1 and altered metabolites such as creatine and L-malic acid. These results highlight the role of metabolic disturbances such as glycine, serine, and threonine metabolism and the PI3K-Akt signaling pathway in TD pathology. The study provides valuable biomarkers and insights into the mechanisms of TD, offering potential targets for future therapeutic and diagnostic strategies.

## Introduction

The patellofemoral joint represents a complex articulation with precise functional and biomechanical demands. The geometry of the trochlear groove plays a critical role in maintaining the stability of this joint, which is further influenced by the intricate interplay of dynamic and static stabilizers [1]. Trochlear dysplasia (TD) is a common lower-extremity deformity, characterized by abnormal anatomy of the medial or lateral facets of the trochlear groove, resulting in either insufficient depth or altered angles. This leads to improper movement of the patella within the joint and poor contact between the patella and femur. Such abnormalities can cause joint pain, a sense of instability, and other skeletal-related issues [2,3]. Some researchers also regard TD as a significant risk factor for early-onset patellofemoral osteoarthritis [4].

As a major pathological factor contributing to patellar instability, the development of TD may be associated with excessive femoral anteversion, patella alta, and an increased tibial tubercle to trochlear groove distance [5,6]. However, the molecular mechanisms underlying its occurrence and development at the molecular level are currently not well understood. Previous animal experimental studies have indicated that early patellar instability can impact the normal development of the femoral trochlear groove in young rabbits, resulting in developmental abnormalities of the groove [7,8]. Therefore, early diagnosis and intervention are crucial to reducing the incidence of patellofemoral joint diseases and improving clinical outcomes for patients with TD.

In recent research, Xu et al. identified potential genes and targets related to the transcriptomic changes in patellar instability [9]. Similarly, Ma et al. validated the association between subchondral bone loss in the femoral trochlea and the activation of JAK1/STAT3 [10]. Both molecular pathways play critical roles in bone metabolism, regulating the growth, proliferation, differentiation, apoptosis, and senescence of bone marrow mesenchymal stem cells, osteoblasts, and osteoclasts. Therefore, understanding these bone metabolism changes in TD could enhance early diagnosis and treatment strategies.

Proteomics and metabolomics offer a holistic view of biological processes, from protein expression to small-molecule metabolite activity, enabling detailed analysis of the effects of dysplasia on skeletal tissues. These advanced techniques can detect low-abundance proteins and metabolites, providing insights into subtle molecular changes over time.

Multi-omics technologies have made significant progress in the study of various diseases, such as osteoarthritis and femoral head necrosis, and have produced encouraging results [11,12]. However, no studies have specifically examined the interaction between metabolomics and proteomics in TD. Therefore, this study aimed to investigate the alterations in metabolites and proteomes in a rat model of TD using a multi-omics approach. By providing new insights into the disease’s pathogenesis, the research seeks to enhance understanding of the underlying mechanisms and explore potential therapeutic strategies to address the clinical challenges posed by TD.

## Materials and methods

### Study design and surgical technique

This study was approved by the Ethics Committee of the Third Hospital of Hebei Medical University (Z2024-009-1).

A total of 24 male Sprague-Dawley rats, aged three weeks, were obtained from the Laboratory Animal Center of Hebei Medical University. As rats require approximately 12 weeks to reach skeletal maturity [13], the animals were acclimatized for one week before being randomly assigned to two groups: the control group (Group C, n = 12) and the experimental group (Group M, n = 12). In Group C, no surgical intervention was performed on the left knees of the rats. In Group M, the left knees underwent surgery to induce patellar instability. Sample size was determined based on previous studies involving similar models of TD [13,14] and recommendations from the literature. A retrospective power analysis conducted post-hoc indicated that a sample size of 6 rats per group provided sufficient power (>80%) to detect significant differences (effect size of 1.5, alpha = 0.05) in primary outcomes such as sulcus angle and subchondral bone parameters.

### Anesthesia and analgesia

Rats were anesthetized using pentobarbital sodium (30 mg/kg, intraperitoneal injection) prior to surgery to ensure deep sedation and pain relief. Postoperative pain management was provided with daily administration of acetaminophen (30 mg/kg) for five days. Additionally, efforts were made to monitor the animals closely for any signs of pain or distress during the recovery period, and appropriate actions were taken to ensure their welfare.

### Surgical procedure

To create the TD model, a medial joint capsule incision was performed. Following previously described protocols [9,10], a midline skin incision was made, followed by separation of the skin and subcutaneous tissue to expose the joint capsule. A 0.5 cm longitudinal incision was made along the medial capsule and patellar retinaculum, inducing patellar instability without causing damage to the cartilage. The incision site was then irrigated, sutured, and dressed with sterile bandages.

### Efforts to alleviate suffering

Postoperative care included regular monitoring for signs of discomfort, with additional analgesia administered as needed. Rats were allowed free access to food and water and housed under standard laboratory conditions. To prevent disuse atrophy, all rats were subjected to 60 minutes of daily treadmill exercise following surgery. This physical activity was implemented to ensure rat activity and promote recovery.

### Methods of sacrifice

At eight weeks post-surgery, rats were euthanized using an overdose of pentobarbital sodium (150 mg/kg, intraperitoneal injection), ensuring humane euthanasia according to AVMA guidelines. Following euthanasia, samples from the distal femur were harvested, rapidly frozen in liquid nitrogen, and stored in cryogenic vials at −80°C for subsequent analysis.

### Macroscopic morphological and micro-CT analysis assessment

**Fig 1:**
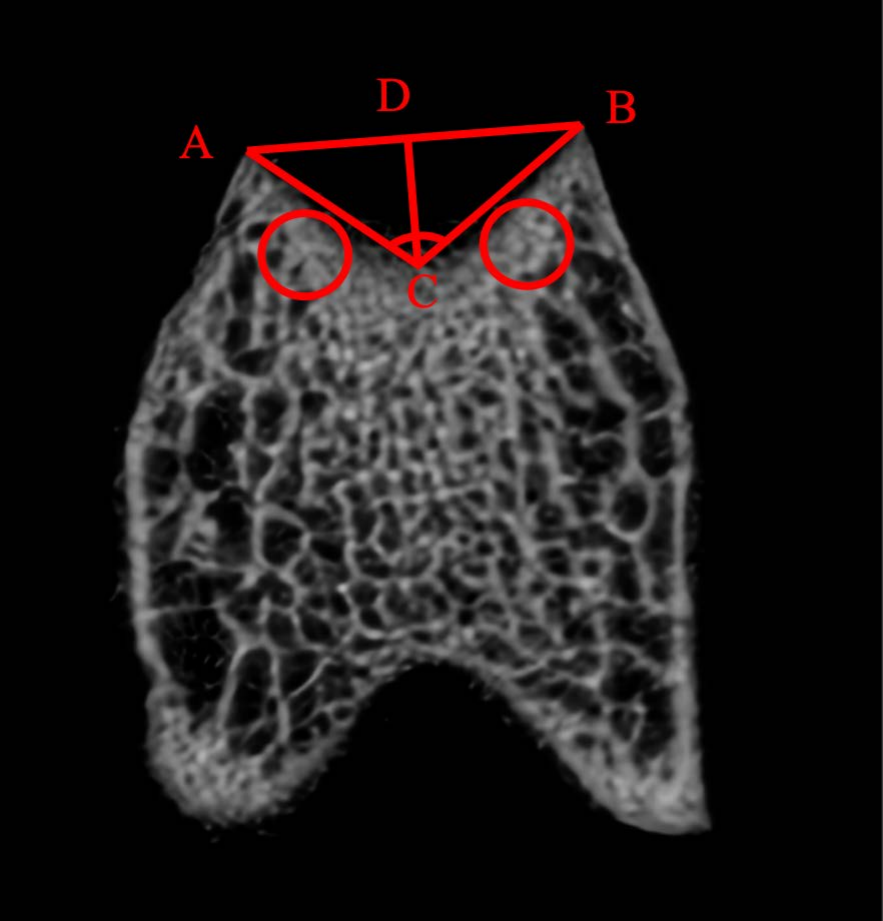
Measurement diagram. DC, trochlear depth; ACB, sulcus angle.

Six femur specimens from each of Groups C and M were selected for micro-CT analysis. All distal femurs were scanned via micro-CT. The micro-CT scans were acquired using the SkyScan 1076 system (Bruker MicroCT, Kontich, Belgium) with the following optimized parameters: 10 μm isotropic voxel size, 0.5 mm aluminum filter, 50 kV tube voltage, 800 μA current, and 180° rotation with 0.4° rotation step. Image reconstruction utilized NRecon software (v1.7.4.2) with beam hardening correction set to 30% and ring artifact reduction level 5. As described previously [15], axial slices of the trochlea were identified; trochlear depth and sulcus angle were then calculated (Fig 1). Using the diagnostic criteria defined by Dejour et al. [16], TD was diagnosed in micro-CT images. For analysis of microstructural parameters, the region of interest was located transversely below the lateral and medial facets of the trochlea with two red cylinders of 3-mm diameter (Fig 1); micro-CT scanning data were transformed into a three-dimensional model by using Mimics software, version 19.0 (Materialise, Leuven, Belgium). Based on previous studies [13], we selected the following representative parameters to evaluate changes in subchondral bone: bone volume to total volume fraction (BV/TV, %), trabecular number (TB.N, mm^-1^), trabecular thickness (TB.Th, mm), trabecular separation (TB.Sp, mm), and bone mineral density (BMD, mg/cm^3^).

### Metabolomic analysis

#### Metabolites extraction.

Distal femur tissues from six rats per group (Groups C and M) were processed using standardized protocols. Precisely 25 mg of tissue was homogenized in 500 μL ice-cold extraction solvent (methanol:water = 3:1 v/v) containing isotopically-labelled internal standards (Waters #186006312). The homogenization procedure consisted of three cycles of mechanical disruption (35 Hz, 4 min) interspersed with ultrasonic treatment (5 min, 4°C ice-water bath). After 1 h incubation at −40°C, samples were centrifuged at 12,000 rpm (RCF = 13,800 × g, radius = 8.6 cm) for 15 min at 4°C. The supernatants were transferred to certified LC-MS vials (Waters #186005408cv) and stored at −80°C until analysis. Quality control (QC) samples were prepared by pooling equal volumes from all individual supernatants.

#### LC-MS/MS analysis.

Chromatographic separation was performed on a Waters ACQUITY UPLC HSS T3 column (2.1 × 100 mm, 1.8 μm) maintained at 45°C using a Vanquish UHPLC system (Thermo Scientific). The mobile phase consisted of (A) 5 mmol/L ammonium acetate with 0.1% formic acid in ultrapure water and (B) 0.1% formic acid in LC-MS grade acetonitrile. The gradient elution program was delivered at 0.4 mL/min as follows: 0–2 min, 2% B; 2–25 min, 2–100% B (linear); 25–28 min, 100% B; 28–30 min, 2% B.

Mass spectrometric detection was conducted using an Orbitrap Exploris 120 instrument (Thermo Scientific) equipped with a HESI-II ion source. Key parameters included: sheath gas flow 50 arb, auxiliary gas 15 arb, capillary temperature 320°C, spray voltage ±3.8 kV (positive/negative mode), full MS resolution 60,000 (@200 m/z), and MS/MS resolution 15,000. Data acquisition employed information-dependent acquisition (IDA) mode with m/z range 100–1500 and stepped normalized collision energy (10/30/60%).

### Quality control

1 Instrument performance was validated through weekly calibration using ESI-L Low Concentration Tuning Mix (Agilent #G1969-85000). Process stability was monitored by injecting QC samples every 10 experimental runs, with acceptance criteria requiring >85% of metabolic features showing relative standard deviation (RSD) <15%. Carryover was assessed through solvent blank injections after every 20 samples.

#### Data analysis for metabolomics.

Raw data files were converted to mzXML format using ProteoWizard (v3.0.20212) and processed through an optimized workflow in R (v4.3.1). Peak detection utilized the CentWave algorithm (Δm/z = 15 ppm, SNR = 6) with retention time alignment via Obiwarp (m/z tolerance = 0.015 Da). Metabolite annotation was performed against the BiotreeDB v5.0 in-house library with a match score threshold of 0.3. Data normalization applied QC-based robust LOESS correction prior to statistical analysis.

### Proteomics analysis

#### Sample preparation.

Among the rats used for metabolomics analysis, distal femur tissues of three rats in each group were selected for proteomics analysis. The remaining three rats per group (total six rats) served as backup animals in case of sample loss or unexpected technical difficulties and were not utilized in the final analyses. Samples were prepared through a series of steps. Initially, all samples were ground in liquid nitrogen at 35 Hz for 4 minutes in steel tubes. Half of each ground sample was mixed with 500 μL of RIPA working solution and sonicated in an ice water bath for 20 minutes to fully lyse the cells. The lysates were then centrifuged at 12,000 rpm, 4°C for 10 minutes, and the supernatants were transferred to new EP tubes.

Following protein extraction, a BCA assay was conducted by adding BCA working solution to a 96-well plate, with each well receiving 200 μL. Samples (diluted accordingly) or BSA standard protein were added, and the plate was shaken at 37°C for 30 minutes. Absorbance was measured at 562 nm to calculate protein concentrations based on a standard curve generated from BSA standards.

Subsequently, acetone precipitation was performed by diluting each sample containing 100 μg of total protein to approximately 1 mg/mL with H2O. Acetone, pre-cooled to −20°C, was added at a 5-fold volume to each sample, followed by overnight precipitation at −20°C. After centrifugation at 12,000 rpm, 4°C for 10 minutes, the supernatants were carefully removed. The pellets were washed twice with 200 μL of pre-cooled 80% acetone and centrifuged again to remove the supernatant.

#### Protein digestion and labeling.

The next step involved redissolving, reducing, and alkylating the proteins. Each sample was resuspended in 100 μL of protein redissolving solution and briefly centrifuged. Dithiothreitol (DTT) was added to a final concentration of 5 mM and incubated at 55°C for 20 minutes to reduce disulfide bonds. After cooling to room temperature, Iodoacetamide (IAA) was added to a final concentration of 15 mM and incubated in the dark for 30 minutes to alkylate the reduced disulfide bonds.

Following protein preparation, trypsin digestion was carried out by dissolving trypsin in Resuspension buffer to 0.5 μg/μL and incubating at room temperature for 5 minutes. After 5-minute activation at room temperature, samples were then mixed with trypsin at a ratio of 1:50 (trypsin to protein) and incubated overnight at 37°C with shaking. Digestion efficiency was verified by 12% SDS-PAGE analysis showing complete disappearance of protein bands >10 kDa.

After digestion, Tandem Mass Tag (TMT) labeling was performed by centrifuging the samples at high speed and transferring equal amounts of protein to new EP tubes. Labeling reactions were conducted in 50 mM HEPES buffer (pH 8.5) containing 30% (v/v) acetonitrile for 1 hr at 25°C. TMT labeling efficiency was assessed by MS/MS spectral analysis of randomly selected peptides, achieving >98% modification rate through quantification of labeled/unlabeled peptide ratio using Proteome Discoverer 2.4. Excess reagents were quenched with 5% (v/v) hydroxylamine in 50 mM HEPES (pH 8.5) for 15 min prior to pooling labeled samples.

Subsequently, SDC cleanup was performed by adding TFA to the samples (final concentration 2%) and centrifuging at high speed for 10 minutes. The supernatant was transferred to new EP tubes, and the peptides were extracted by adding 1000 μL of 2% TFA, followed by centrifugation at 12,000 rpm for 10 minutes (repeated twice). The combined supernatants were collected for further analysis.

The peptides were desalted using C18 columns by first activating them with Buffer C, followed by equilibration with Buffer A. The sample supernatant was slowly eluted through the columns, and after washing with Buffer A and eluting with Buffer B, the eluates were collected and vacuum-dried overnight at 4°C.

Finally, high-pH pre-fractionation was performed on the dried peptides by reconstituting them in mobile phase A and fractionating under alkaline conditions using RPUPLC. Fractions were collected, pooled, and stored at −80°C after vacuum drying.

#### NanoLCMS/MS analysis.

For each sample, 2μL of total peptides were separated and analyzed with a nanoUPLC (EASYnLC1200) coupled to a Q Exactive HFX Orbitrap instrument (Thermo Fisher Scientific) with a nanoelectrospray ion source. Separation was performed using a reversedphase column (100 μm ID × 15 cm, ReprosilPur 120 C18AQ, 1.9 μm, Dr. Maisch). Mobile phases were H2O with 0.1% formic acid (FA), 2% ACN (phase A) and 80% ACN, 0.1% FA (phase B). Separation of sample was executed with a 90 min gradient at 300 nL/min flow rate. Gradient B: 25% for 2 min, 522% for 68 min, 2245% for 16 min, 4595% for 2 min, 95% for 2 min.

Data dependent acquisition (DDA) was performed in profile and positive mode with Orbitrap analyzer at a resolution of 120,000 (@200 m/z) and m/z range of 3501600 for MS1; For MS2, the resolution was set to 15k with a fixed first mass of 110 m/z. The automatic gain control (AGC) target for MS1 was set to 3E6 with max IT 30 ms, and 1E5 for MS2 with max IT 96 ms. The top 20 most intense ions were fragmented by HCD with normalized collision energy (NCE) of 32%, and isolation window of 0.7 m/z. The dynamic exclusion time window was 45 s, single charged peaks and peaks with charge exceeding 6 were excluded from the DDA procedure.

#### Data analysis for proteomics.

Vendor’s raw MS files were processed using Proteome Discoverer (PD) software (Version 2.4.0.305) and the built-in Sequest HT search engine. MS spectra lists were searched against their specieslevel UniProt FASTA databases (uniprot-Rattus norvegicus-10116-2022-11. fast), with Carbamidomethyl [C], TMT 6 plex(K) and TMT 6 plex (N-term) as a fixed modification and Oxidation (M) and Acetyl (Protein Nterm) as variable modifications. Trypsin was used as proteases. A maximum of 2 missed cleavage(s) was allowed. The false discovery rate (FDR) was set to 0.01 for both PSM and peptide levels. Peptide identification was performed with an initial precursor mass deviation of up to 10 ppm and a fragment mass deviation of 0.02Da. Unique peptide and Razor peptide were used for protein quantification and total peptide amount for normalization. All the other parameters were reserved as default. Fold change thresholds (≤ 0.83 or ≥ 1.2) were chosen based on widely accepted criteria in proteomic studies for robust identification of biologically meaningful differences [17]. Representative raw data are provided in the supplementary material (S1 File).

#### Integrative analysis.

The correlation-based analysis between differentially expressed proteins (DEPs) and differentially expressed metabolites (DEMs) was conducted using R and the “*corrplot*” package. And differentially expressed protein-differentially expressed metabolite (DEP-DEM) interaction networks were generated using OmicsNet 2.0. The input data for the DEP and DEM names were converted to UniProt and KEGG IDs. Multiple databases, including KEGG, Recon3D, and STRING, were used to create independent networks for each omics network. The relationship between differential proteins and differential metabolites was visualized with p < 0.05, |r| = 1.

### Statistical analysis

The identification of DEPs and DEMs, along with the bioinformatics analysis, was carried out using various R packages in R (version 4.3.1). These included the *DESeq2, edgeR*, and *limma* packages, which were instrumental in performing statistical analyses to identify DEPs and DEMs present in varying quantities. For graphical representation, the ggplot2 package was used. The ropls packagewas employed for a comprehensive analysis of multivariate data, and the clusterProfiler package was essential for performing enrichment analysis. Additionally, the integration with biological databases was achieved seamlessly through the Biostrings and GenomicFeatures packages. All analyses involving outcome measurements (micro-CT and morphometric analysis) were conducted in a blinded manner, where investigators were unaware of group assignments to avoid bias. Data are presented as the mean ± standard deviation. Imaging data were compared using paired Student’s t-test and the Wilcoxon matched-pairs signed rank test for nonnormally distributed data. Multiple comparisons were corrected using the Benjamini-Hochberg FDR approach. Only results with FDR-adjusted p < 0.05 were considered significant.

## Results

### Gross morphology and micro‑CT assessment

**Fig 2:**
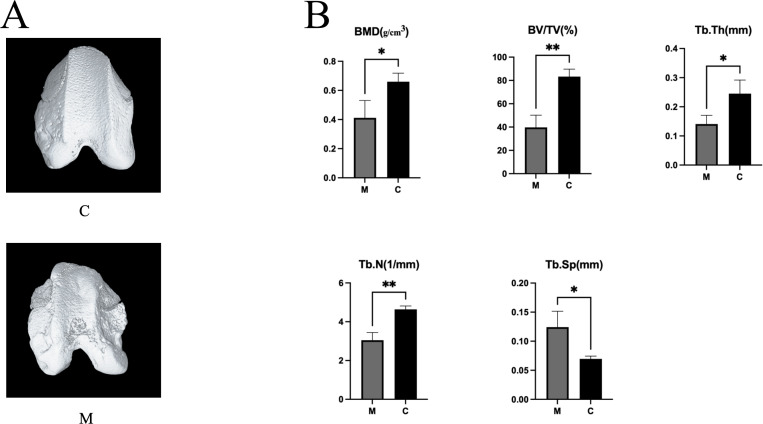
The micro-CT results. A. The trochlear depth and sulcus angle of Group M were regarded as normal values. The trochlear groove became significantly flatter and sulcus angle became significantly larger, compared with the Group C at 8 weeks after surgery; B. In the Group M, micro-CT displayed obvious loss of subchondral bone at 8 weeks after surgery. *p < 0.05; **p < 0.01. Abbreviations: BMD: bone mineral density; BV/TV: bone volume to total volume; TB.Th: trabecular thickness; TB.N: trabecular number; TB.Sp: trabecular separation; Group C, the control group; Group M, the experimental group.

In Group M, significant morphological alterations in the trochlear groove were observed compared to Group C at 8 weeks post-surgery (Table 1, Fig 2). These changes, characteristic of TD, include a notable increase in the sulcus angle, indicative of a flatter trochlear groove (Group M: 145.0° ± 3.0°; Group C: 132.0° ± 3.4°, p = 0.022). Additionally, the trochlear depth was significantly reduced in Group M, reflecting a shallower groove (Group M: 0.9 ± 0.2 mm; Group C: 1.5 ± 0.2 mm, p = 0.017). These findings underscored we successfully established an experimental model of TD in Group M.

**Table 1:** Sulcus angle and trochlear depth measurements compared between the two groups.

	Group C	Group M	p value
Sulcus angle	132.0° ± 3.4°	145.0° ± 3.0°	0.022
Trochlear depth	1.5 ± 0.2 mm	0.9 ± 0.2 mm	0.017

Mean ± standard deviation; Group C, the control group; Group M, the experimental group.

### Detection of subchondral bone loss by micro-CT

At 8 weeks post-surgery, Group M exhibited substantial bone loss compared to Group C, characterized by progressive decreases in bone mass and notable reductions in subchondral bone plate thickness (Fig 2A). Micro-CT analysis revealed significant differences in trabecular parameters between the groups, as detailed below (Fig 2B): The reduction in BV/TV was statistically significant (Group M: 39.70 ± 10.33%; Group C: 83.29 ± 6.50%, p = 0.004), reflecting a marked loss of bone volume in Group M. Tb.Th was significantly reduced in Group M (Group M: 0.14 ± 0.03 mm; Group C: 0.24 ± 0.05 mm, p = 0.032), indicating thinning of the trabecular structure. Tb.N showed a significant reduction in Group M (Group M: 3.05 ± 0.40 mm ⁻ ¹; Group C: 4.64 ± 0.17 mm ⁻ ¹, p = 0.003), suggesting a decrease in trabecular density. Tb.Sp was significantly increased in Group M (Group M: 0.124 ± 0.02 mm; Group C: 0.070 ± 0.004 mm, p = 0.027), reflecting reduced connectivity and greater separation between trabeculae. The reduction in BMD in Group M was statistically significant (Group M: 0.4115 ± 0.1192 g/cm³; Group C: 0.6590 ± 0.0594 g/cm³, p = 0.032). These findings confirm that patellar instability in Group M leads to significant subchondral bone loss and microstructural deterioration, consistent with the pathophysiology of TD.

### Metabolomics of rat model with TD

#### Metabolism analysis by LC-MS/MS.

**Fig 3:**
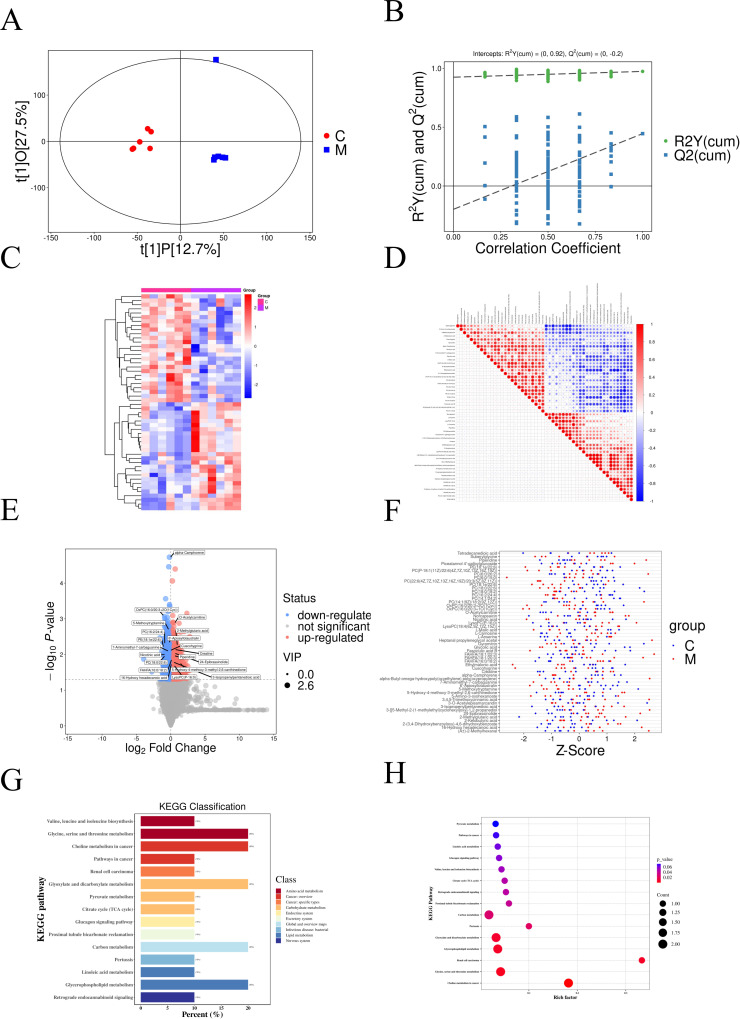
The metabolomics analysis results. A. Orthogonal projection to latent structures discriminant analysis (OPLS-DA) plot. PCA score plot showing clustering between TD and control groups. One TD sample displayed modest separation from the main cluster but was retained following quality control assessment; B. Permutation plot; C. Heatmap of differentially expressed metabolites; D. Correlation heatmap for the 52 differentially expressed metabolites (based on the VIP). Red indicates a positive correlation, blue indicates a negative correlation, and the larger the round node size is, the greater the correlation coefficient between the two metabolites; E. Volcano plot representing candidate metabolites. Red represents upregulated metabolites, and blue represents downregulated metabolites; F. Z-score plot. The points of different colors represent samples in different groups. The distribution of each differential metabolite between different groups can be seen very intuitively; G. Horizontal distribution plots of differential metabolites in KEGG pathway enrichment analysis. The x-axis represents the ratio (%) of differentially expressed metabolites annotated to each metabolic pathway compared to the total number of differentially expressed metabolites annotated to KEGG pathways. The y-axis represents the pathway names; H. Bubble plots of differential metabolites in KEGG pathway enrichment analysis. The x-axis represents the rich factor, and the y-axis represents the top 15 pathway information. Larger bubbles indicate a greater number of differential metabolites, and the color of the bubbles changes from purple to red, indicating a smaller p-value and greater significance. Abbreviations: Group C, the control group; Group M, the experimental group.

Non-targeted metabolomic analysis was performed using highresolution MS detection technology and identification with an internal library of authentic chemical standards. Based on the orthogonal projection to latent structures discriminant analysis (OPLS-DA) analysis, we found that there were great differences between the Groups C and M (Fig 3A). Although one TD sample exhibited slightly weaker separation in the PCA score plot, it passed all quality control criteria and was retained in the analysis. We chose not to exclude it, as the observed variability likely reflects inherent biological heterogeneity. Importantly, this sample did not alter the major findings, and key metabolomic differences remained significant. This supports the robustness of our results. To confirm the stability and robustness of the OPLS-DA model, 7-fold cross-validation and 200 permutation tests were performed. The permutation plots confirmed the stability of the OPLS-DA model (Fig 3B).

### Differences in relative abundances of metabolites between groups

DEMs were identified based on p-values and variable importance in projection (VIP) values, with those having p < 0.05 and VIP > 1 considered significant. Hierarchical clustering analysis of a heatmap visualized the accumulation patterns of 52 selected metabolites from both groups, highlighting differences between them (Fig 3C). Among these, 26 metabolites were upregulated and 26 downregulated. It was worth noting that there was a significant difference in the relative abundance of creatine and L-malate. Creatine was significantly upregulated (log_2_FC = 0.55, p = 0.007), while L-malic acid was significantly downregulated (log_2_FC = −0.41, p = 0.030). Volcano and Z-score plots (Fig 3E and F) illustrated the significant differences in metabolites, with detailed information summarized in Table 2. Correlation analysis, using the Pearson correlation coefficient, explored the relationships between differentially abundant metabolites (Fig 3D). Pathway enrichment analysis of differential metabolites using the KEGG database revealed the top 15 significant pathways, including glycine, serine, and threonine metabolism, the citrate cycle (TCA cycle), and glycerophospholipid metabolism (Fig 3G and H).

**Table 2:** The differential expression metabolites in Group M versus Group C.

Metabolites	HMDB ID	FC	log_2_FC	p value	Up/Down
alpha-Camphorene	HMDB0036852	0.877	−0.189	< 0.001	Down
OxPC(16:0/20:3 + 2O(1Cyc))	–	0.530	−0.916	0.001	Down
O-Acetylcarnitine	–	1.363	0.446	0.001	Up
5-Methoxytryptamine	HMDB0004095	0.901	−0.150	0.002	Down
6’-Apiosyllotaustralin	HMDB0034207	0.761	−0.393	0.005	Down
PC(16:2/24:4)	–	0.631	−0.663	0.005	Down
2-Methylglutaric acid	HMDB0000422	1.667	0.737	0.006	Up
PE(18:1e/22:6)	–	0.542	−0.884	0.006	Down
Creatine	HMDB0000064	1.466	0.552	0.007	Up
Cuscohygrine	HMDB0030290	0.911	−0.135	0.008	Down
7-Aminomethyl-7-carbaguanine	HMDB0011690	0.844	−0.245	0.008	Down
24-Epibrassinolide	HMDB0041130	1.429	0.515	0.009	Up
Nicotinic acid	HMDB0001488	0.592	−0.756	0.010	Down
PC(18:0/22:6)	–	0.596	−0.746	0.011	Down
Piperidine	HMDB0034301	1.367	0.451	0.011	Up
FAHFA(16:0/18:2)	–	1.220	0.287	0.014	Up
Gyromitrin	HMDB0033952	0.439	−1.189	0.015	Down
3-Isopropenylpentanedioic acid	HMDB0032352	1.251	0.323	0.016	Up
PC(P-18:1(11Z)/22:6(4Z,7Z,10Z,13Z,16Z,19Z))	HMDB0011295	0.625	−0.677	0.018	Down
5-Hydroxy-4-methoxy-3-methyl-2,6-canthinedione	HMDB0040798	2.401	1.263	0.018	Up
16-Hydroxy hexadecanoic acid	HMDB0006294	1.204	0.267	0.018	Up
LysoPC(P-16:0)	HMDB0010407	1.331	0.412	0.019	Up
PC(18:1e/22:6)	–	0.599	−0.740	0.022	Down
Fasciculic acid B	HMDB0036438	0.556	−0.848	0.023	Down
LysoPC(18:4(6Z,9Z,12Z,15Z))	HMDB0010389	1.164	0.220	0.024	Up
Ethylmalonic acid	HMDB0000622	0.839	−0.253	0.024	Down
FAHFA(18:1/20:3)	–	1.351	0.434	0.025	Up
alpha-Butyl-omega-hydroxypoly(oxyethylene) poly(oxypropylene)	HMDB0032181	1.707	0.771	0.026	Up
PC(14:1(9Z)/18:2(9Z,12Z))	HMDB0007907	2.133	1.093	0.028	Up
(Â±)-2-Methylhexanal	–	1.686	0.754	0.028	Up
3,4,5-Trimethoxycinnamic acid	HMDB0002511	1.709	0.774	0.028	Up
L-Malic acid	HMDB0000156	0.750	−0.416	0.030	Down
OxPC(16:0/20:3 + 1O(1Cyc))	–	0.599	−0.739	0.033	Down
L-Anserine	–	1.772	0.825	0.034	Up
2-(3,4-Dihydroxybenzoyloxy)-4,6-dihydroxybenzoate	HMDB0059651	1.427	0.513	0.035	Up
Glycolic acid	HMDB0000115	0.795	−0.332	0.037	Down
Norcapsaicin	HMDB0036327	1.260	0.334	0.037	Up
L-Carnosine	–	1.785	0.836	0.039	Up
3-[[5-Methyl-2-(1-methylethyl)cyclohexyl]oxy]-1,2-propanediol	HMDB0036133	1.746	0.804	0.039	Up
Piceatannol 4’-galloylglucoside	HMDB0040862	2.431	1.282	0.040	Up
Heptanal propyleneglycol acetal	HMDB0032302	1.682	0.750	0.041	Up
FAHFA(18:1/22:3)	–	1.164	0.220	0.041	Up
PC(18:0/18:2)	–	0.561	−0.834	0.042	Down
PC(22:6(4Z,7Z,10Z,13Z,16Z,19Z)/20:3(5Z,8Z,11Z))	HMDB0008737	0.647	−0.628	0.043	Down
PC(6:0/26:2)	–	1.956	0.968	0.045	Up
2-Ketobutyric acid	HMDB0000005	0.748	−0.420	0.045	Down
3-O-Acetylepisamarcandin	HMDB0031958	0.849	−0.236	0.045	Down
5-Amino-3-oxohexanoate	–	0.928	−0.108	0.045	Down
Tetradecanedioic acid	HMDB0000872	1.274	0.349	0.047	Up
Suberylglycine	HMDB0000953	0.946	−0.081	0.048	Down
PC(14:1/24:2)	–	0.550	−0.862	0.048	Down
PC(6:0/15:0)	–	0.705	−0.505	0.049	Down

Group C, the control group; Group M, the experimental group; FC, Fold Change.

### Proteomics of rat model with TD

**Fig 4:**
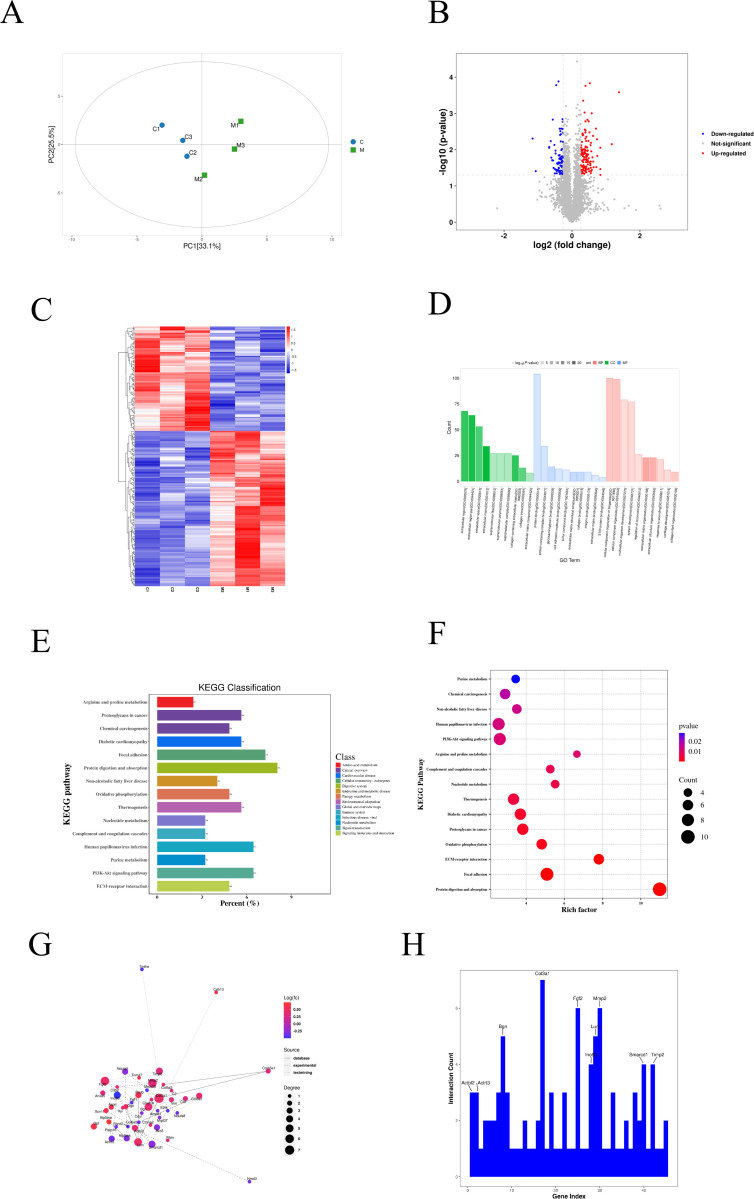
The protein analysis results. A. Principal component analysis of the protein abundances of proteins quantified in rat distal femurs. The abscissa PC1 and ordinate PC2 represent the scores of the first and second principal components, respectively, and the ellipse is the 95% confidence interval; B. Volcano plot representing candidate proteins. Red represents upregulated proteins, and blue represents downregulated proteins; C. Heatmap of differentially expressed proteins; D. GO-based enrichment analysis for the differentially expressed proteins between the trochlear dysplasia group and the normal group; E. Horizontal distribution plots of differential proteins in KEGG pathway enrichment analysis. The x-axis represents the ratio (%) of differentially expressed proteins annotated to each metabolic pathway compared to the total number of differentially expressed proteins annotated to KEGG pathways. The y-axis represents the pathway names; F. Bubble plots of differential proteins in KEGG pathway enrichment analysis. The x-axis represents the enrichment score, and the y-axis represents the top 15 pathway information. Larger bubbles indicate a greater number of differential proteins, and the color of the bubbles changes from purple to red, indicating a smaller p-value and greater significance; G. PPI analysis network based on the STRING database. Red represents a significant up-regulation, blue represents a significant down-regulation; the size of the circle represents the connectivity of differentially expressed proteins, the higher the connectivity, the larger the circle; the type of connection represents the source of the interaction, and the solid line represents the interaction. It comes from the database, the dotted line represents the interaction relationship from experiments, and the dotted line represents the interaction relationship from text mining; H. Protein interaction connectivity histogram. Abbreviations: PPI, protein-protein interaction. Group C, the control group; Group M, the experimental group.

To further investigate the proteomic profile of TD, TMT-labeled proteomic analysis was conducted on TD and normal rat samples. Principal component analysis (PCA) indicated distinct clustering of each group (Fig 4A). A total of 6,165 proteins and 41,046 peptides were identified from all distal femoral bone tissue samples. After data preprocessing, 5,736 proteins were retained, with 204 proteins differentially expressed between the Groups C and M. Of these, 122 proteins were upregulated and 82 were downregulated, with the top 20 differentially expressed proteins listed in Table 3. The volcano plot highlighted Map4k1 as one of the most significantly upregulated proteins in Group M (log_2_FC = 1.38, p < 0.001; Fig 4B). Other significantly altered proteins included Col3a1 (upregulated, log_2_FC = 1.35, p = 0.013) and Ndufs8 (downregulated, log_2_FC = −0.39, p < 0.001). Heatmap analysis, consistent with PCA data, showed clear separation between TD samples and controls (Fig 4C).

**Table 3:** The top 20 of the differential expression proteins in Group M versus Group C.

Gene name	UniProt ID	FC	log_2_FC	p value	Up/Down
Ndufs8	B0BNE6	0.759	−0.398	< 0.001	Down
Adipoq	A0A0G2K845	1.439	0.525	< 0.001	Up
Mrps25	Q4QR80	0.725	−0.464	< 0.001	Down
Nenf	Q6IUR5	1.317	0.397	< 0.001	Up
Map4k1	A0A8I6A6A3	2.611	1.384	< 0.001	Up
Vat1l	M0R3N4	1.257	0.330	< 0.001	Up
Rcn2	A0A8L2QBI0	1.292	0.370	0.001	Up
Rnase4	O55004	1.495	0.581	0.001	Up
Slc8a3	A0A8L2QN54	0.824	−0.279	0.001	Down
Txlnb	A0A0G2K2T1	0.675	−0.566	0.001	Down
Col6a3	A0A8I6ADR1	1.369	0.453	0.001	Up
Fam114a1	A0A8I6GGB2	1.316	0.396	0.002	Up
Ogn	D3ZVB7	1.405	0.491	0.002	Up
Tom1	A0A8I6ACG3	0.826	−0.275	0.002	Down
Olr1121	D3ZM97	1.631	0.705	0.003	Up
Gas6	A0A8I6AC16	0.804	−0.314	0.003	Down
Anapc4	A0A8I5ZJP1	0.777	−0.363	0.003	Down
Mrpl37	Q6AXT0	0.824	−0.279	0.003	Down
Camk1	A0A8L2UL04	1.300	0.379	0.003	Up
Angptl2	G3V862	1.319	0.400	0.003	Up

Group C, the control group; Group M, the experimental group; FC, Fold Change.

Next, Gene Ontology (GO) analysis of the 204 DEPs identified key categories, including extracellular matrix organization, extracellular matrix, and glycosaminoglycan binding in the Biological Process (BP), Cellular Component (CC), and Molecular Function (MF) categories, respectively (Fig 4D). KEGG pathway analysis of DEPs identified the top 15 significant pathways, highlighting their functional categories, p-values, and enrichment factors (Fig 4E and F). The results showed that the differential proteins were predominantly enriched in the PI3K-Akt signaling pathway, ECM-receptor interaction, and focal adhesion pathways, indicating their potential involvement in these critical biological processes. A protein-protein interaction (PPI) network constructed using the STRING database (Fig 4G), highlighted Col3a1 as a key interacting protein, with significant interactions involving Vim, Col16a1, Lum, Mmp2, Bgn, Aspn, and Timp2 (Fig 4H).

### Integrative analysis of DEPs and DEMs

**Fig 5:**
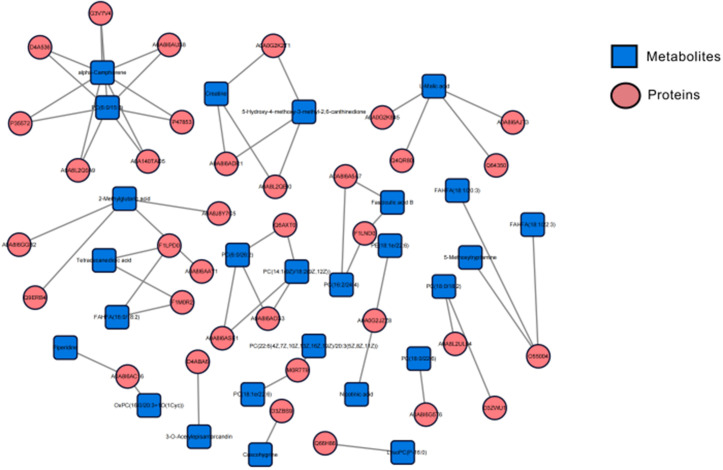
The network figure shows the integration of DEPs and DEMs using Omicsnet 2.0. The individual omics networks of DEPs and DEMs were generated by using the Recon3D, KEGG, and STRING databases, which were subsequently integrated multi-omics networks. To enhance clarity and conciseness, a minimum network setting was taken for the visualization of the metabolite-PPI network. Abbreviations: DEP, differentially expressed protein; DEM, differentially expressed metabolite.

Integrative analysis of proteomics and metabolomics data was performed using Omicsnet 2.0 (https://www.omicsnet.ca/, Fig 5) [18]. Correlation-based analysis revealed that creatine was positively correlated with A0A8I6ADR1 and A0A8L2QBI0, but negatively correlated with A0A0G2K2T1. L-Malic acid showed positive correlations with A0A8I6AJT3, Q64350, and Q4QR80, and negative correlations with A0A0G2K845. Additionally, LysoPC (P-16:0) was negatively correlated with Q66H86.

## Discussion

This study is the first to reveal molecular changes in the femoral distal bone tissue of a rat model of TD by integrating proteomics and metabolomics approaches. We identified significant differences between the proteome and metabolome profiles of TD and normal bone tissue. Notably, Col3a1, creatine, and L-malic acid were identified as key players in TD. The discovery of metabolic pathways such as glycine, serine, and threonine metabolism, as well as the PI3K-Akt signaling pathway, offers crucial insights into the pathophysiology of TD. Further investigation into these pathways could elucidate related molecular mechanisms and provide new therapeutic targets.

TD is a common skeletal developmental disorder that severely affects patients’ quality of life and physical function. We successfully established a rat model of TD induced by patellar instability during the growth period, characterized by subchondral bone loss and shallowing of the TD [14]. Imaging analysis confirmed the validity and reliability of the model, providing a solid foundation for subsequent proteomic and metabolomic studies. Patellar instability is a major cause of mobility disorders in adolescents with a complex pathology [19]. TD is a critical factor in this context, but its molecular mechanisms remain unclear [20]. Large datasets from proteomics and metabolomics require sophisticated bioinformatics analysis. With advances in computational capabilities and data analysis techniques, researchers can now better interpret the biological significance of these datasets, leading to new hypotheses and experimental designs. Combining proteomics and metabolomics allows for the systematic analysis of multiple cellular and tissue-level processes, facilitating a deeper understanding of the molecular mechanisms underlying developmental disorders [21]. To date, no studies have utilized metabolomics and proteomics to investigate the mechanism of TD. The DEPs and DEMs identified here may become the focus of future research and therapeutic interventions. Further functional studies can explore the specific roles of these molecules in bone development, stability, and repair, providing theoretical support for the development of new intervention strategies.

In our proteomic analysis, we found Col3a1 to be significantly upregulated in Group M. Col3 plays a critical role in skeletal development, as it is required for the growth acceleration of osteoblasts and may help preserve the osteogenic potential of mesenchymal stem cells [22]. Research also suggests that Col3 is involved in trabecular bone formation and maintenance through its direct effects on osteogenesis [23,24]. Additionally, Col3a1 has been linked to musculoskeletal injuries [25]. Its upregulation may affect both osteogenesis and cartilage structural integrity, which could be closely related to TD development. Abnormal expression of Col3 could not only directly hinder osteogenesis but also contribute to cartilage structural disorganization and decreased stability [25], potentially contributing to the pathogenesis of TD.

GO analysis revealed that DEPs were significantly associated with extracellular matrix (ECM) organization, the ECM itself, and glycosaminoglycan binding. The ECM plays a key role in supporting tissue structure and participating in various physiological processes, including cell signaling and cell-matrix interactions. Changes in ECM can directly impact tissue mechanics and structure, leading to abnormal development or impaired function [26]. Glycosaminoglycan binding is essential for ECM function and signal regulation, and abnormalities here may affect the biochemical properties of the matrix and its signaling capabilities [27].

Additionally, our study showed that DEPs were enriched in the PI3K-Akt signaling pathway, a key component in cell development, metabolism, apoptosis, and the cell cycle [28]. Our previous study showed that TD-induced cartilage degradation may be associated with the activation of the PI3K-Akt signaling pathway [13], which is consistent with our results. This confirms that the PI3K-Akt signaling pathway plays a key role in the pathogenesis of TD. Other pathways such as proteoglycans in cancer, ECM-receptor interaction, and focal adhesion have not been widely studied in TD but have attracted significant attention in knee diseases such as cartilage degeneration [29], osteoarthritis [30], and reduced patellar tendon function [31]. Therefore, exploring the link between these pathways and TD remains essential for future studies.

Metabolomics has become widely applied in joint disease research, so we utilized UPLC-MS/MS to extract and analyze bone tissue metabolites. We observed involvement of pathways such as glycine, serine, and threonine metabolism, glycerophospholipid metabolism, the TCA cycle, and glyoxylate and dicarboxylate metabolism in TD development.

Our study revealed that Group M showed reduced bone density and subchondral bone loss, while creatine content significantly increased, which may be associated with femoral trochlear bone remodeling. Creatine participates in glycine, serine, and threonine metabolism and is involved in the creatine/phosphocreatine system, which plays a crucial role in bioenergetic processes, especially in tissues with high metabolic demands such as skeletal muscle and bone [32]. Recent studies suggest that creatine may influence bone remodeling and support muscle strength in aging individuals. Creatine is also involved in glycine formation, and previous research has shown a negative correlation between serum glycine levels and cortical bone strength and fracture risk [33]. This could explain the subchondral bone loss observed in the Group M. Therefore, creatine, as a key metabolite in the glycine, serine, and threonine metabolic pathways, suggests the occurrence of bone remodeling in the context of patellar instability.

Malic acid is an important metabolite in the TCA cycle, and naturally occurring malic acid is the L-type. The TCA cycle is a key metabolic pathway that provides energy for osteoblast differentiation and plays a critical role in bone synthesis. In mature osteoblasts, glycolysis generates most of the energy required for osteoblast function maintenance [34,35]. The well-known osteogenic factor bone morphogenetic protein can promote glucose metabolism, thereby promoting the development of bone and cartilage [36]. Additionally, malic acid is involved in the glyoxylate and dicarboxylate metabolism pathways, which are also related to bone homeostasis [37]. Xu et al. demonstrated that increased lactoferrin could regulate bone formation through the niacin and niacinamide metabolism pathways, promoting bone mass formation in growing animals [24]. Our findings of reduced malic acid levels in the Group M may reflect impaired femoral trochlear development due to patellar instability, where insufficient metabolic energy disrupts osteoblast and osteoclast differentiation, impairing bone homeostasis and femoral development, ultimately leading to TD.

LysoPC (P-16:0) is a type of glycerophospholipids, which are the most abundant phospholipids in the body and regulate intracellular molecular signaling pathways by binding to G-protein-coupled receptors on biological membranes through phospholipase hydrolysis. They play an essential role in the metabolism of various processes, including inflammation, immunity, and tumor growth, which are crucial in the onset and progression of many diseases [38]. Abnormal accumulation of metabolites like PC(14:1(9Z)/18:2(9Z,12Z)) and LysoPC(P-16:0), along with disruptions in glycerophospholipid metabolism, may contribute to the abnormal development of the femoral trochlea.

While our study uncovered several molecular pathways significantly altered in the TD group, these changes should be interpreted as correlative patterns derived from cross-sectional multi-omics data. Rather than implying direct causality, the observed alterations-such as ECM remodeling and activation of PI3K-Akt signaling—may represent adaptive responses to changes in joint mechanics or exercise-induced remodeling. Recognizing this distinction is essential for guiding future mechanistic investigations and therapeutic development. Importantly, the rat model used in this study replicates key anatomical and pathological features of human TD, yet interspecies differences in joint biomechanics and developmental context should be taken into account when considering clinical translation. Our findings therefore serve as a foundation for future validation studies in human tissues and functional experiments employing genetic or pharmacologic modulation of candidate pathways. Notably, the variable expression of Col3a1 and the context-dependent roles of the PI3K-Akt pathway reported in previous studies highlight the complexity of cartilage biology across different disease settings. Rather than limiting interpretation, these aspects underscore the importance of integrative approaches and provide opportunities for deeper insight into the molecular landscape of TD.

Given that patellar instability primarily affects adolescents, early diagnosis and intervention are crucial for improving clinical outcomes. Our study enhances understanding of TD’s biological mechanisms, which could aid in developing more effective therapeutic strategies. We hope our findings contribute to clinical practice and serve as a basis for future research. Moving forward, we will continue to explore the biological significance of these findings and seek new collaborations to translate basic research into clinical applications. The use of proteomics and metabolomics in this study demonstrates the power of these technologies in uncovering complex disease mechanisms, offering guidance and inspiration for future research in related fields.

Although this is the first study to use metabolomics and proteomics to investigate femoral distal bone tissue in a rat model of TD, there are several limitations. First, the species and sex of the animals used may influence the results, and these findings may not directly translate to humans; future studies should include a broader range of subjects. Second, the sample size was relatively small, and validation in larger cohorts is needed. Third, while we identified differential proteins and metabolites, their specific functions in TD are not fully elucidated. Although strong correlations were observed, causation cannot be established, and alternative explanations such as secondary responses to mechanical instability should be considered. Fourth, key proteomic findings were not validated using methods like Western blot or immunohistochemistry. Finally, targeted metabolomic validation of key metabolites, including creatine and L-malic acid, was not performed, and future studies should employ LC-MS/MS-based approaches to confirm these findings.

Another limitation is the absence of functional assessments such as gait analysis or physical examination to detect patellar instability phenotypes. Although treadmill exercise was used to simulate mechanical loading, changes in joint kinematics or signs of recurrent dislocation were not systematically evaluated. Future work should include behavioral or biomechanical endpoints (e.g., quantitative gait tracking, kinematic analysis, or in vivo imaging) to better correlate molecular changes with joint function. Moreover, it remains unclear whether the molecular alterations observed are specific to TD or reflect general joint pathology. Comparative studies with other joint disease models will help clarify this. Finally, integrating transcriptomics and epigenetics in future multi-omics designs will further advance our understanding of TD and its regulatory networks.

In summary, this study provides a novel perspective on TD, revealing potential molecular mechanisms through proteomics and metabolomics. Our findings offer important insights for future treatment strategies, with both academic and clinical significance. We believe that continued research in this field will lead to new advancements in human health and help tackle the clinical challenges associated with TD. Future studies should validate identified molecular targets using gene knockdown, overexpression, or pharmacological inhibitors. Evaluating their therapeutic potential in preclinical TD models will help translate these findings into clinical applications.

## Conclusions

This study integrates proteomic and metabolomic analyses to uncover molecular alterations in a rat model of TD. Significant findings include upregulation of Col3a1 and altered metabolites such as creatine and L-malic acid. These results highlight the role of metabolic disturbances such as glycine, serine, and threonine metabolism and the PI3K-Akt signaling pathway in TD pathology. The study provides valuable biomarkers and insights into the mechanisms of TD, offering potential targets for future therapeutic and diagnostic strategies.

## Supporting information

S1 File (ZIP)

## Abbreviations

TD: Trochlear dysplasia
CT: Computed tomography
BV/TV: Bone volume to total volume
TB.N: Trabecular number
TB.Th: Trabecular thickness
TB.Sp: Trabecular separation
BMD: Bone mineral density
QC: Quality control
LC-MS/MS: Liquid chromatography–tandem mass spectrometry
UPLC: Ultra-performance liquid chromatography
IDA: Information-dependent acquisition
MS: Mass spectrometry
DDA: Data-dependent acquisition
FA: Formic acid
TMT: Tandem Mass Tag
DTT: Dithiothreitol
IAA: Iodoacetamide
FDR: False discovery rate
PPI: Protein-protein interaction
PCA: Principal component analysis
OPLS-DA: Orthogonal projections to latent structures discriminant analysis
GO: Gene Ontology
KEGG: Kyoto Encyclopedia of Genes and Genomes
ECM: Extracellular matrix
DEM: Differentially expressed metabolite
DEP: Differentially expressed protein
VIP: Variable importance in projection
FC: Fold change
AGC: Automatic gain control
EP: Eppendorf
SDS-PAGE: Sodium dodecyl sulfate–polyacrylamide gel electrophoresis.
