# Supplementary material for: Multi-Omics insights into the molecular mechanisms of trochlear dysplasia: A proteomic and metabolomic study in rats
Source: PLoS One. 2025 Aug 11;20(8):e0325562. doi: 10.1371/journal.pone.0325562 (PMC12338795; doi:10.1371/journal.pone.0325562)
Supplement: S1 File — (ZIP) [file pone.0325562.s001.zip › S1_File/Metabolomic analysis/Statistical Analysis/C-M/DonutPlot.pdf]

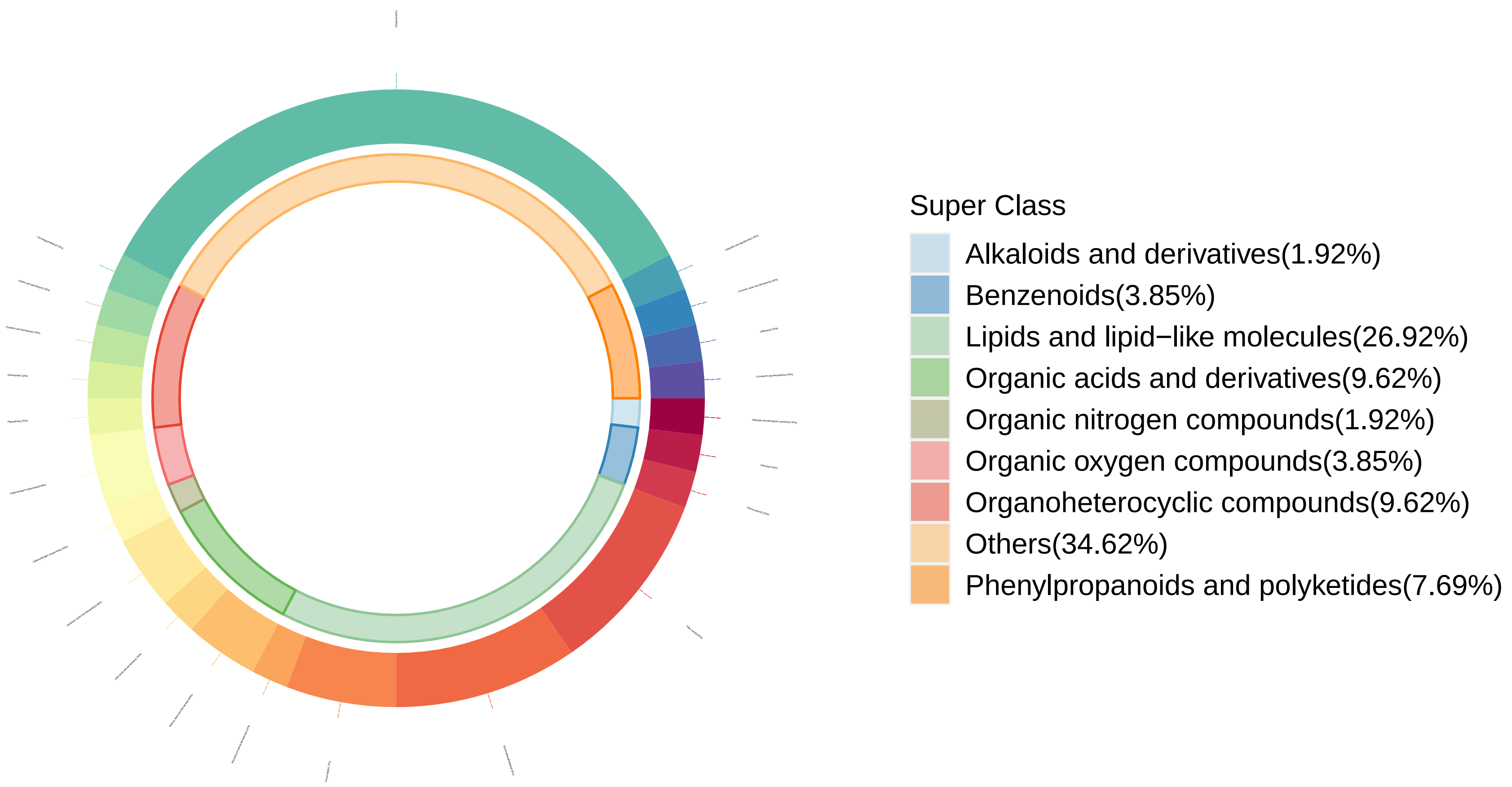
Super Class
Alkaloids and derivatives(1.92%)
Benzenoids(3.85%)
Lipids and lipid−like molecules(26.92%)
Organic acids and derivatives(9.62%)
Organic nitrogen compounds(1.92%)
Organic oxygen compounds(3.85%)
Organoheterocyclic compounds(9.62%)
Others(34.62%)
Phenylpropanoids and polyketides(7.69%)
