# Supplementary material for: Multi-Omics insights into the molecular mechanisms of trochlear dysplasia: A proteomic and metabolomic study in rats
Source: PLoS One. 2025 Aug 11;20(8):e0325562. doi: 10.1371/journal.pone.0325562 (PMC12338795; doi:10.1371/journal.pone.0325562)
Supplement: S1 File — (ZIP) [file pone.0325562.s001.zip › S1_File/Metabolomic analysis/Statistical Analysis/M-C/OPLS-DA permutation histogram.pdf]

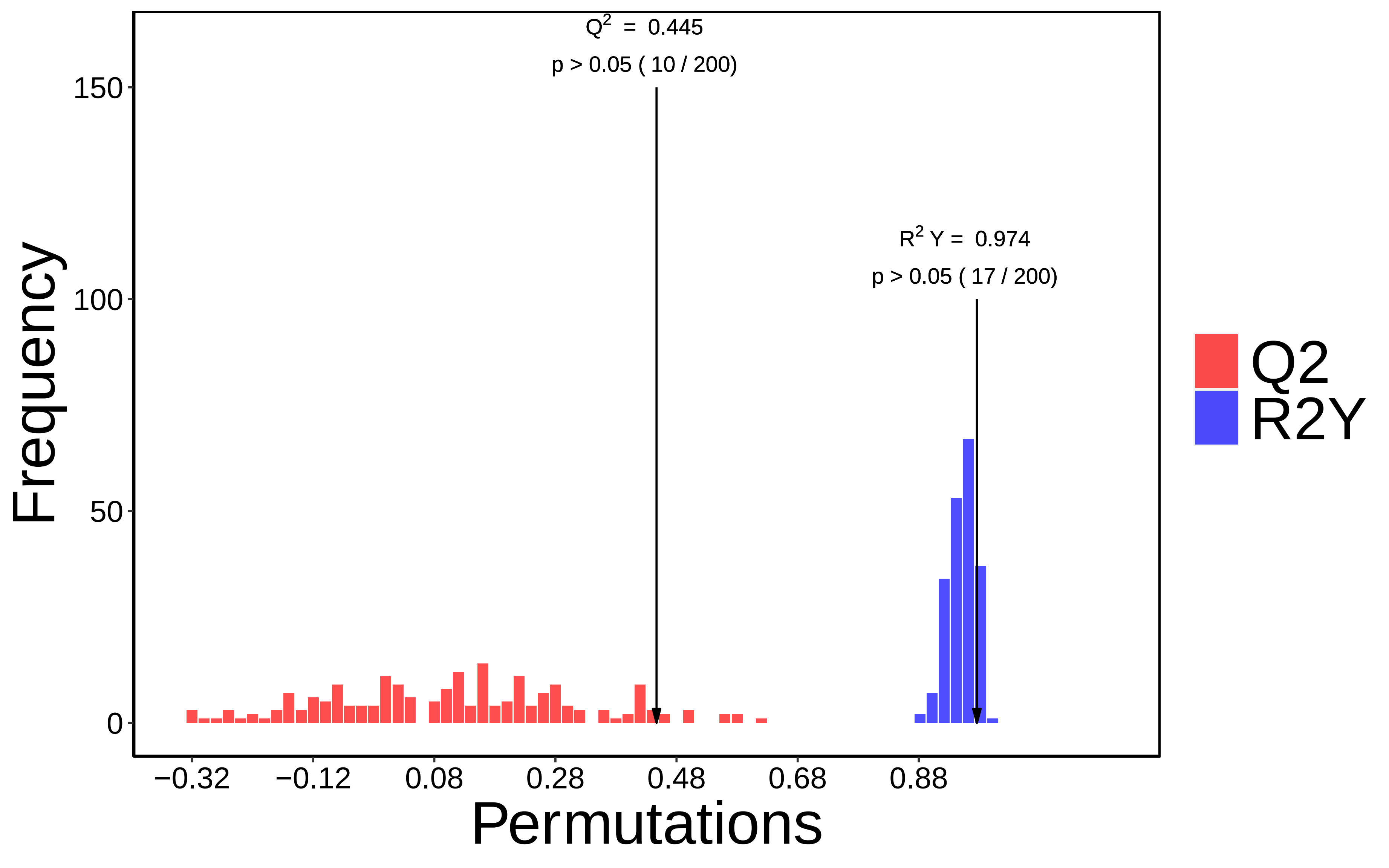

Q2 = 0.445
p > 0.05 (10 / 200)
R2 Y = 0.974
p > 0.05 (17 / 200)
150
100
50
0
Frequency
−0.32
−0.12
0.08
0.28
0.48
0.68
0.88
Permutations
Q2
R2Y
