# Supplementary material for: Multi-Omics insights into the molecular mechanisms of trochlear dysplasia: A proteomic and metabolomic study in rats
Source: PLoS One. 2025 Aug 11;20(8):e0325562. doi: 10.1371/journal.pone.0325562 (PMC12338795; doi:10.1371/journal.pone.0325562)
Supplement: S1 File — (ZIP) [file pone.0325562.s001.zip › S1_File/Metabolomic analysis/Statistical Analysis/TOTAL/DonutPlot.pdf]

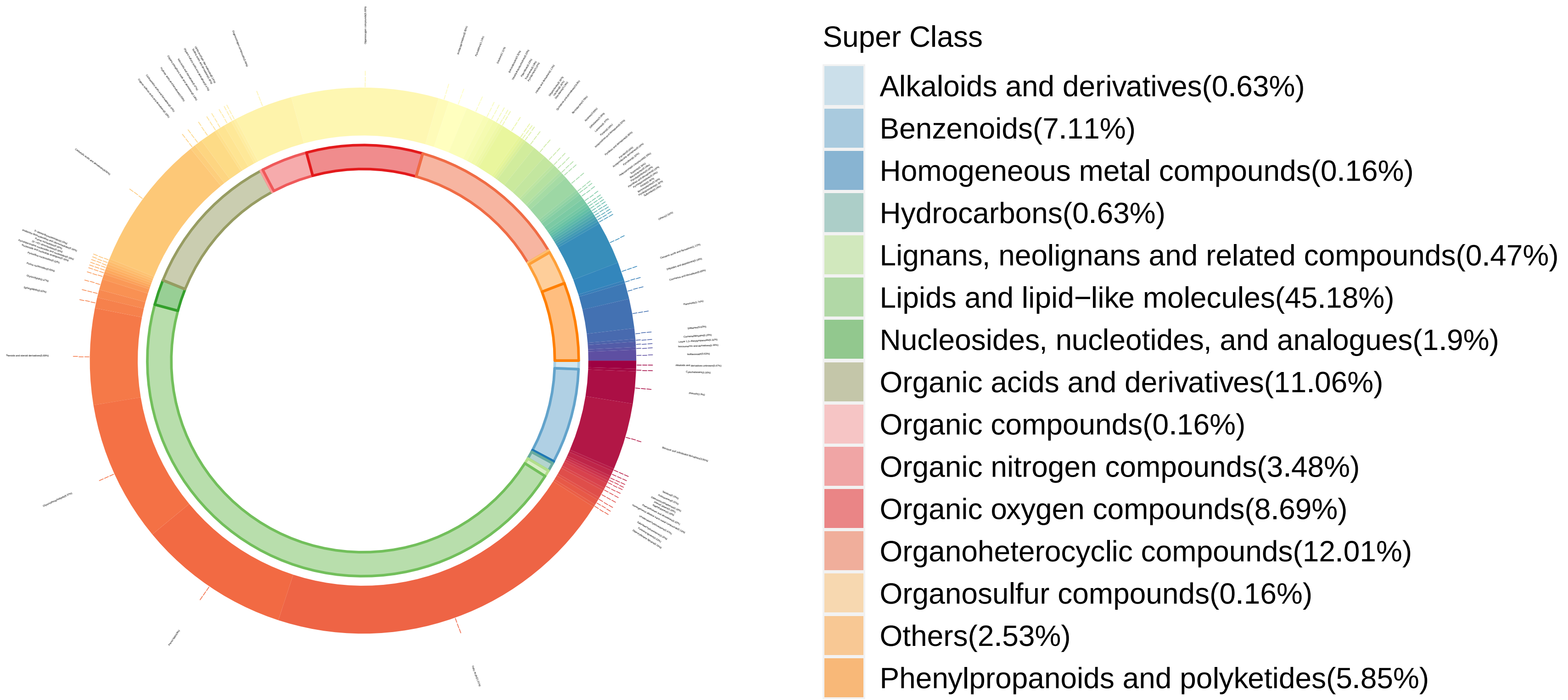

Super Class
Alkaloids and derivatives(0.63%)
Benzenoids(7.11%)
Homogeneous metal compounds(0.16%)
Hydrocarbons(0.63%)
Lignans, neolignans and related compounds(0.47%)
Lipids and lipid−like molecules(45.18%)
Nucleosides, nucleotides, and analogues(1.9%)
Organic acids and derivatives(11.06%)
Organic compounds(0.16%)
Organic nitrogen compounds(3.48%)
Organic oxygen compounds(8.69%)
Organoheterocyclic compounds(12.01%)
Organosulfur compounds(0.16%)
Others(2.53%)
Phenylpropanoids and polyketides(5.85%)
