# Supplementary material for: Multi-Omics insights into the molecular mechanisms of trochlear dysplasia: A proteomic and metabolomic study in rats
Source: PLoS One. 2025 Aug 11;20(8):e0325562. doi: 10.1371/journal.pone.0325562 (PMC12338795; doi:10.1371/journal.pone.0325562)
Supplement: S1 File — (ZIP) [file pone.0325562.s001.zip › S1_File/Metabolomic analysis/Statistical Analysis/TOTAL/pie plot.pdf]

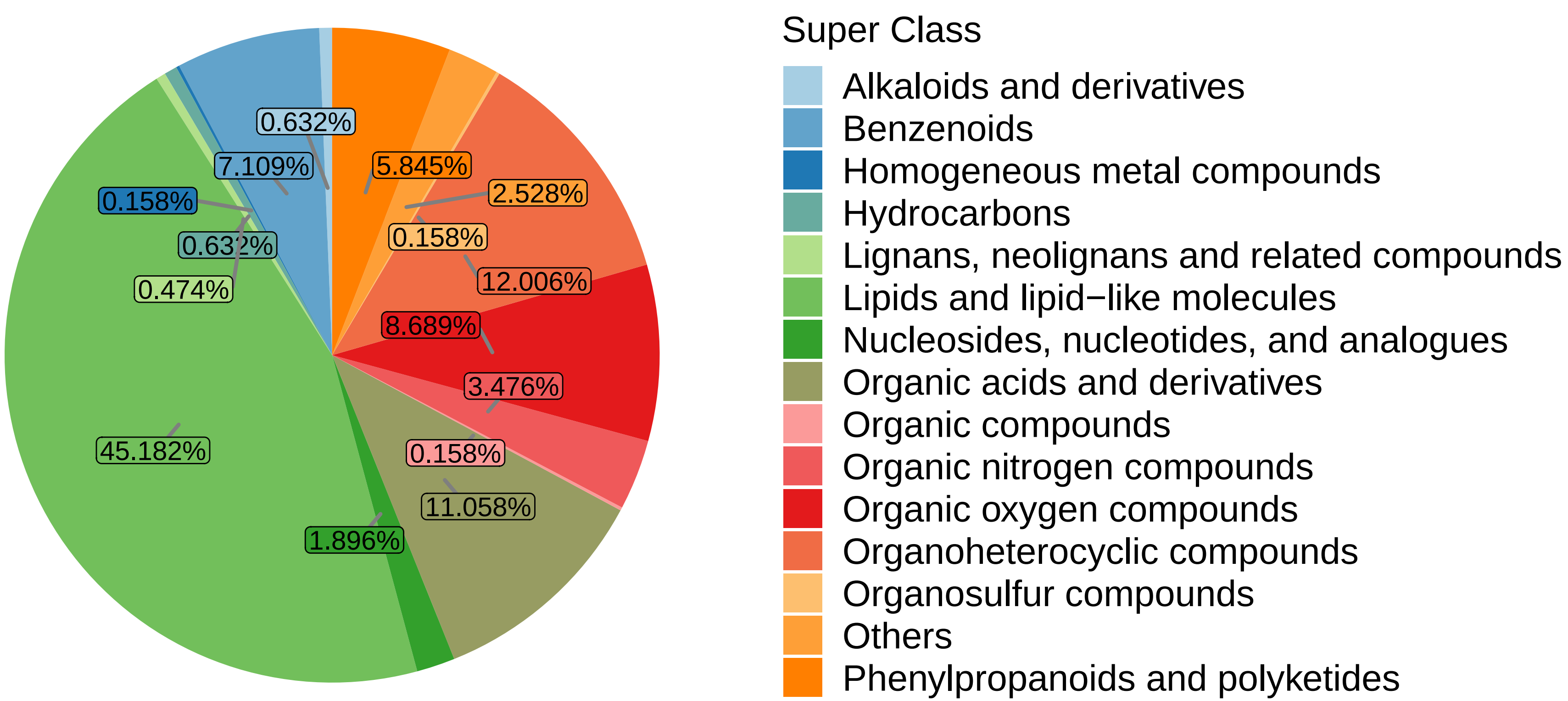

0.632%
7.109%
0.158%
0.632%
0.474%
45.182%
1.896%
5.845%
2.528%
0.158%
12.006%
8.689%
3.476%
0.158%
11.058%
Super Class
Alkaloids and derivatives
Benzenoids
Homogeneous metal compounds
Hydrocarbons
Lignans, neolignans and related compounds
Lipids and lipid−like molecules
Nucleosides, nucleotides, and analogues
Organic acids and derivatives
Organic compounds
Organic nitrogen compounds
Organic oxygen compounds
Organoheterocyclic compounds
Organosulfur compounds
Others
Phenylpropanoids and polyketides
