# Supplementary material for: Multi-Omics insights into the molecular mechanisms of trochlear dysplasia: A proteomic and metabolomic study in rats
Source: PLoS One. 2025 Aug 11;20(8):e0325562. doi: 10.1371/journal.pone.0325562 (PMC12338795; doi:10.1371/journal.pone.0325562)
Supplement: S1 File — (ZIP) [file pone.0325562.s001.zip › S1_File/Metabolomic analysis/Enrichment Analysis/C-M/KEGG Classification.pdf]

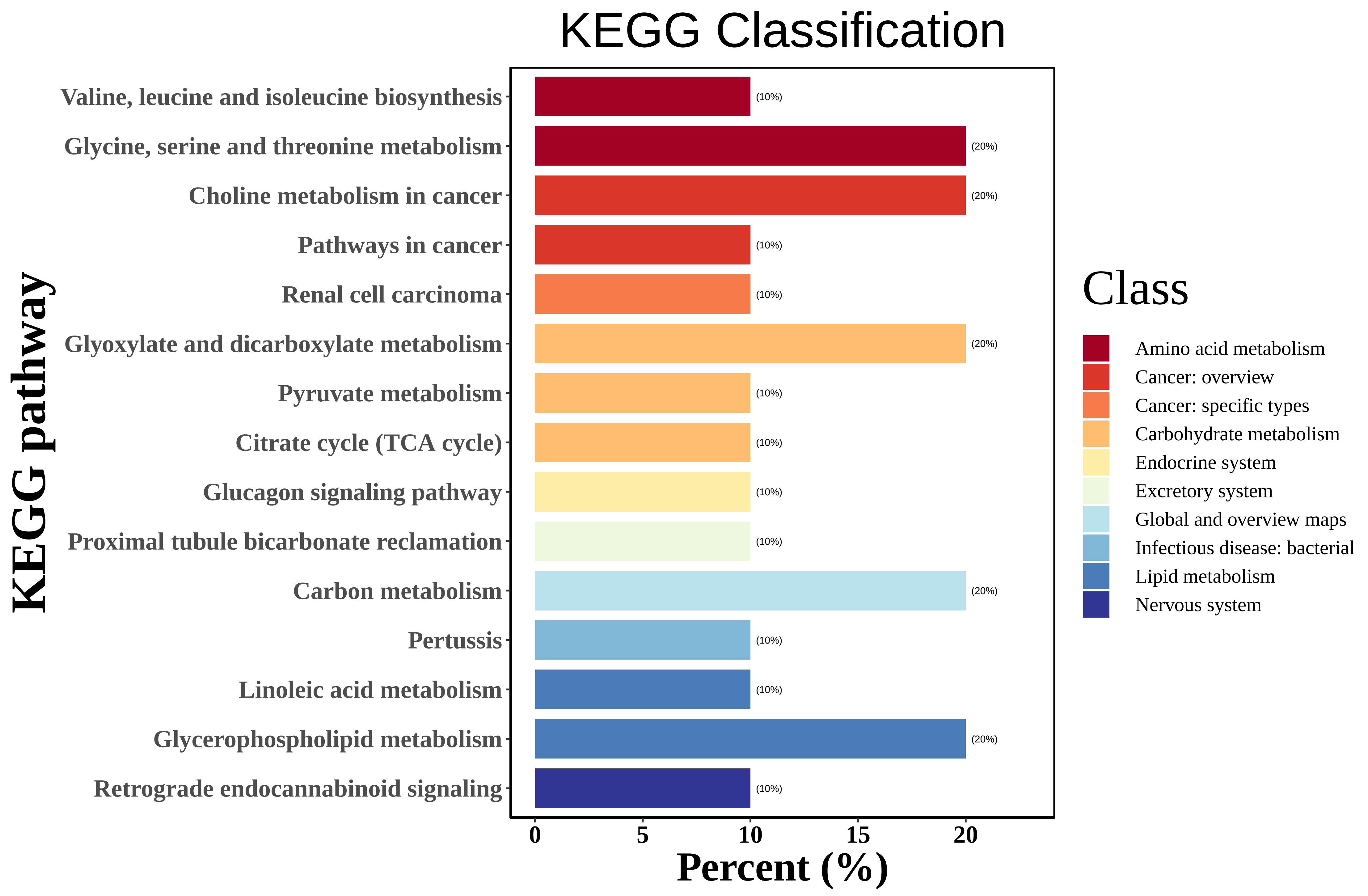
KEGG Classification
KEGG pathway
Valine, leucine and isoleucine biosynthesis
Glycine, serine and threonine metabolism
Choline metabolism in cancer
Pathways in cancer
Renal cell carcinoma
Glyoxylate and dicarboxylate metabolism
Pyruvate metabolism
Citrate cycle (TCA cycle)
Glucagon signaling pathway
Proximal tubule bicarbonate reclamation
Carbon metabolism
Pertussis
Linoleic acid metabolism
Glycerophospholipid metabolism
Retrograde endocannabinoid signaling
(10%)
(20%)
(20%)
(10%)
(10%)
(20%)
(10%)
(10%)
(10%)
(10%)
(20%)
(10%)
(10%)
(20%)
(10%)
0
5
10
15
20
Percent (%)
Class
Amino acid metabolism
Cancer: overview
Cancer: specific types
Carbohydrate metabolism
Endocrine system
Excretory system
Global and overview maps
Infectious disease: bacterial
Lipid metabolism
Nervous system
