# Supplementary material for: Multi-Omics insights into the molecular mechanisms of trochlear dysplasia: A proteomic and metabolomic study in rats
Source: PLoS One. 2025 Aug 11;20(8):e0325562. doi: 10.1371/journal.pone.0325562 (PMC12338795; doi:10.1371/journal.pone.0325562)
Supplement: S1 File — (ZIP) [file pone.0325562.s001.zip › S1_File/Metabolomic analysis/Enrichment Analysis/C-M/KEGG DA Score plot.pdf]

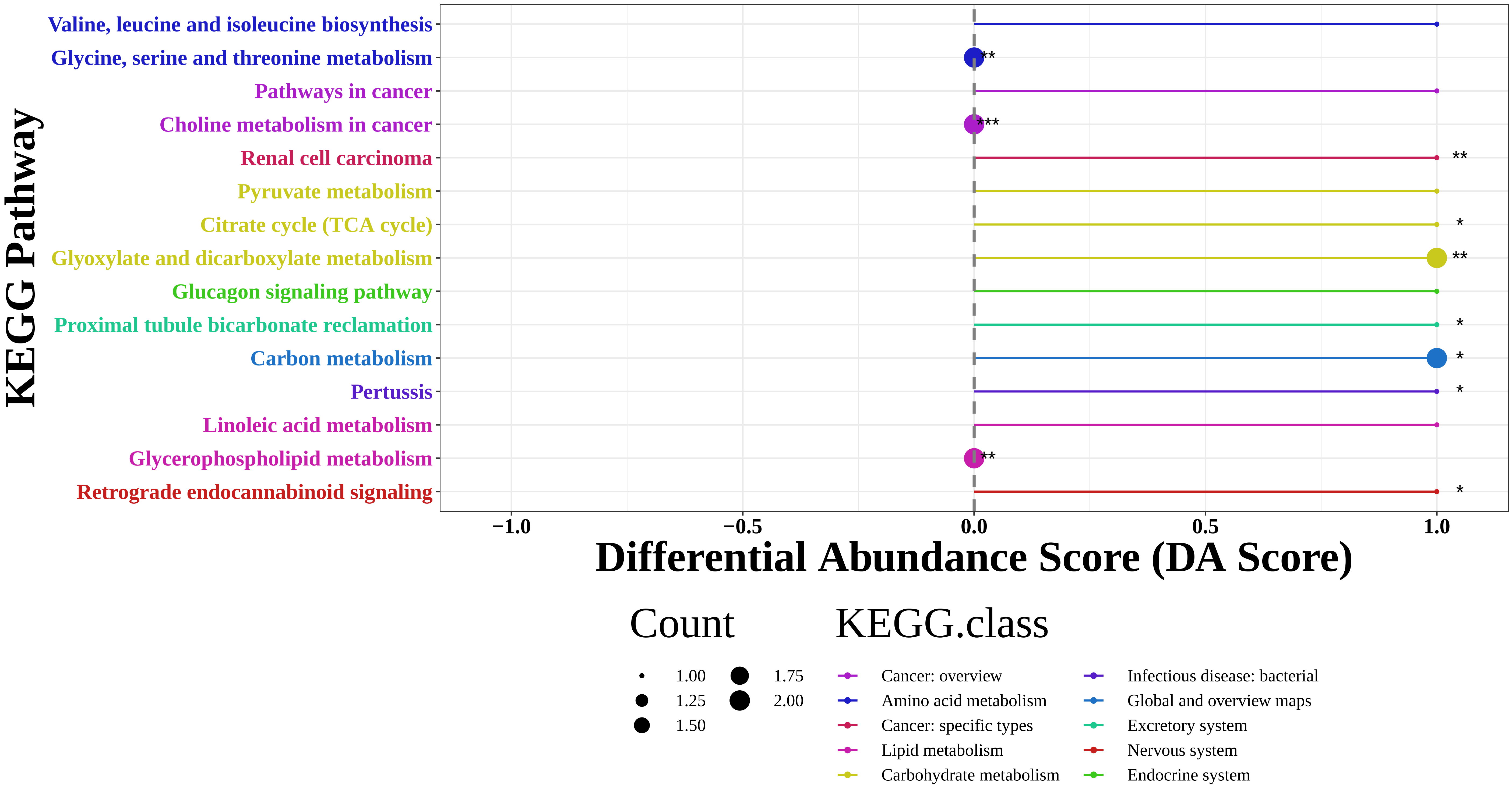
KEGG Pathway
Valine, leucine and isoleucine biosynthesis
Glycine, serine and threonine metabolism
Pathways in cancer
Choline metabolism in cancer
Renal cell carcinoma
Pyruvate metabolism
Citrate cycle (TCA cycle)
Glyoxylate and dicarboxylate metabolism
Glucagon signaling pathway
Proximal tubule bicarbonate reclamation
Carbon metabolism
Pertussis
Linoleic acid metabolism
Glycerophospholipid metabolism
Retrograde endocannabinoid signaling
**
***
**
*
**
*
*
*
**
*
−1.0
−0.5
0.0
0.5
1.0
Differential Abundance Score (DA Score)
Count
1.00
1.25
1.50
1.75
2.00
KEGG.class
Cancer: overview
Amino acid metabolism
Cancer: specific types
Lipid metabolism
Carbohydrate metabolism
Infectious disease: bacterial
Global and overview maps
Excretory system
Nervous system
Endocrine system
