# Supplementary material for: Multi-Omics insights into the molecular mechanisms of trochlear dysplasia: A proteomic and metabolomic study in rats
Source: PLoS One. 2025 Aug 11;20(8):e0325562. doi: 10.1371/journal.pone.0325562 (PMC12338795; doi:10.1371/journal.pone.0325562)
Supplement: S1 File — (ZIP) [file pone.0325562.s001.zip › S1_File/Proteomics analysis/KEGG Analysis/M_vs_C/KEGG Classification.pdf]

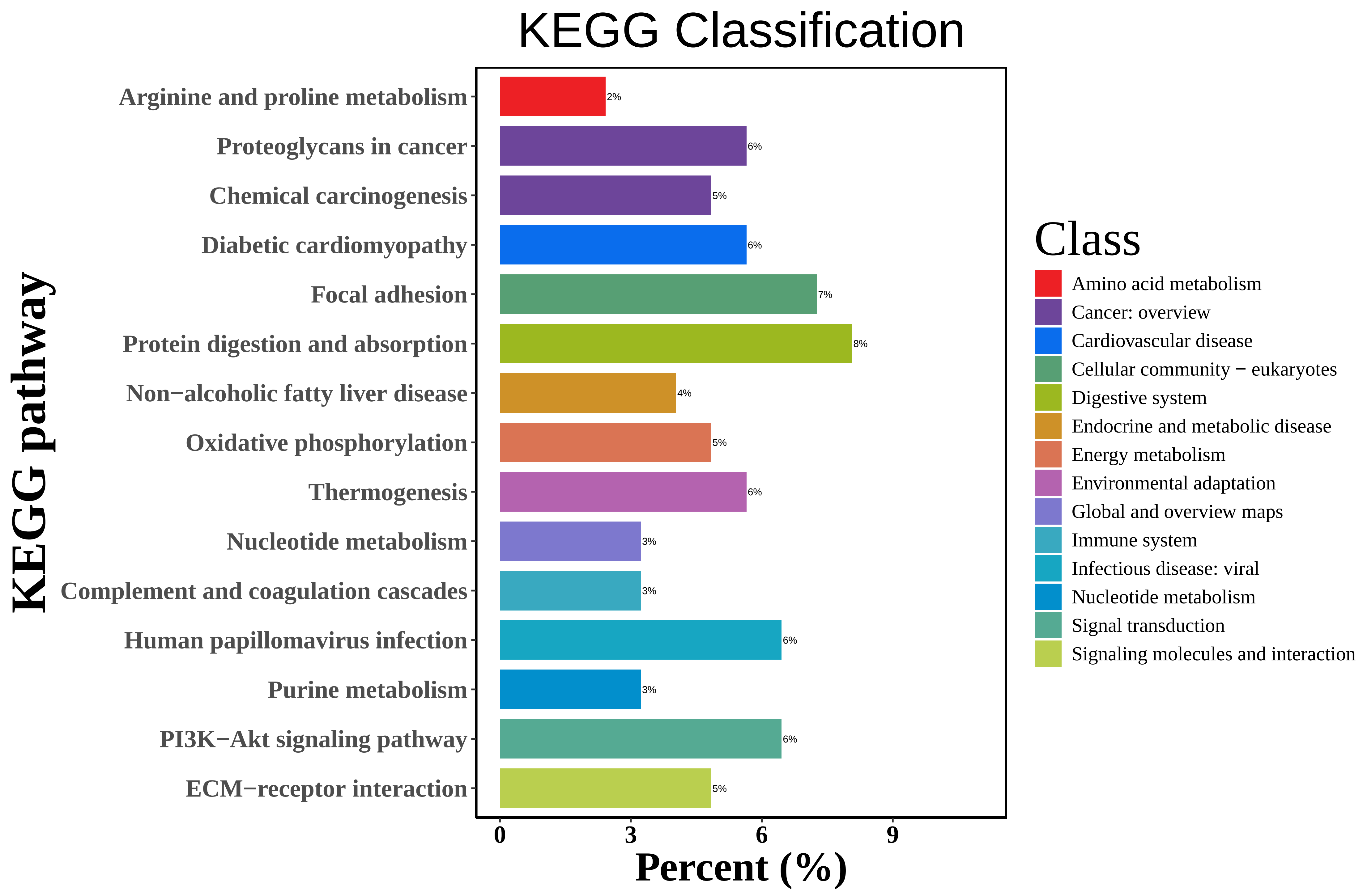

KEGG Classification
KEGG pathway
Arginine and proline metabolism
Proteoglycans in cancer
Chemical carcinogenesis
Diabetic cardiomyopathy
Focal adhesion
Protein digestion and absorption
Non−alcoholic fatty liver disease
Oxidative phosphorylation
Thermogenesis
Nucleotide metabolism
Complement and coagulation cascades
Human papillomavirus infection
Purine metabolism
PI3K−Akt signaling pathway
ECM−receptor interaction
2%
6%
5%
6%
7%
8%
4%
5%
6%
3%
3%
6%
3%
6%
5%
0
3
6
9
Percent (%)
Class
Amino acid metabolism
Cancer: overview
Cardiovascular disease
Cellular community − eukaryotes
Digestive system
Endocrine and metabolic disease
Energy metabolism
Environmental adaptation
Global and overview maps
Immune system
Infectious disease: viral
Nucleotide metabolism
Signal transduction
Signaling molecules and interaction
