# Supplementary figures and images for: Multi-Omics insights into the molecular mechanisms of trochlear dysplasia: A proteomic and metabolomic study in rats
Source: PLoS One. 2025 Aug 11;20(8):e0325562. doi: 10.1371/journal.pone.0325562 (PMC12338795; doi:10.1371/journal.pone.0325562)

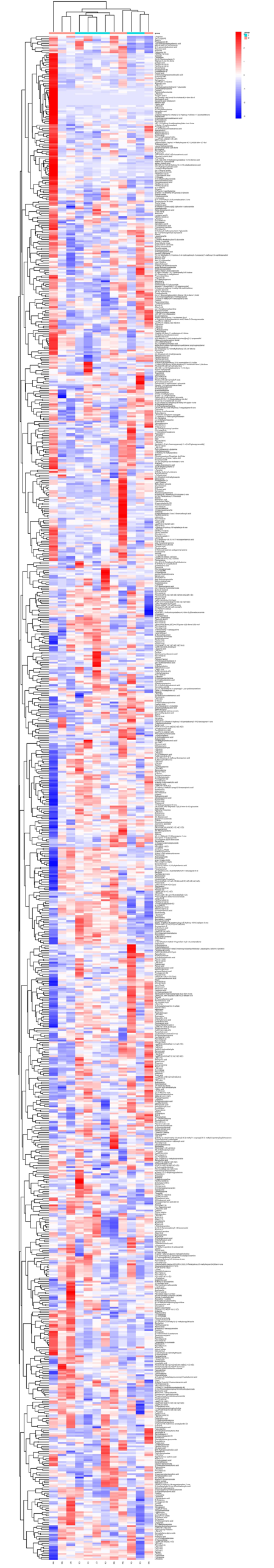

Supplement: S1 File — (ZIP) [file pone.0325562.s001.zip › S1_File/Metabolomic analysis/Hierarchical Clustering Analysis/TOTAL-heatmap.jpg]

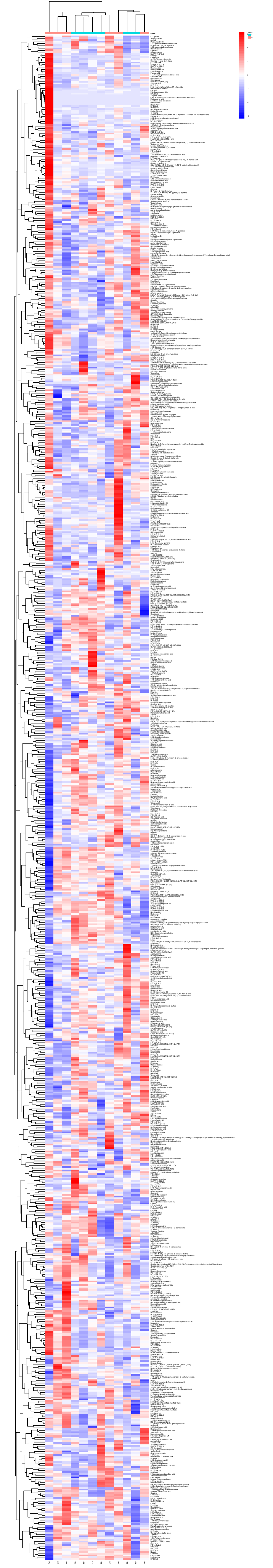

Supplement: S1 File — (ZIP) [file pone.0325562.s001.zip › S1_File/Metabolomic analysis/Hierarchical Clustering Analysis/TOTAL-heatmap.pdf]

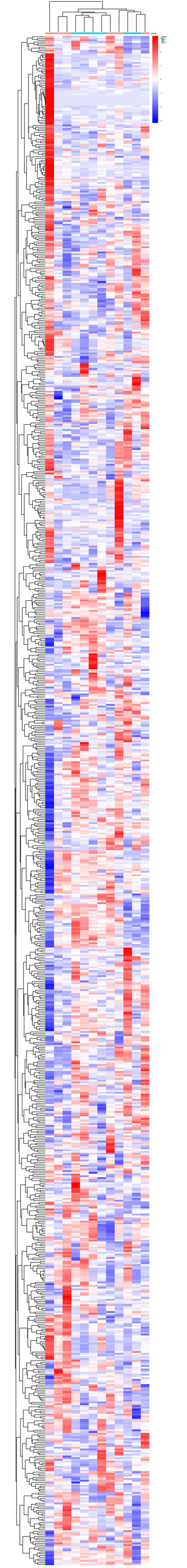

Supplement: S1 File — (ZIP) [file pone.0325562.s001.zip › S1_File/Metabolomic analysis/Hierarchical Clustering Analysis/TOTAL-heatmap no label.jpg]

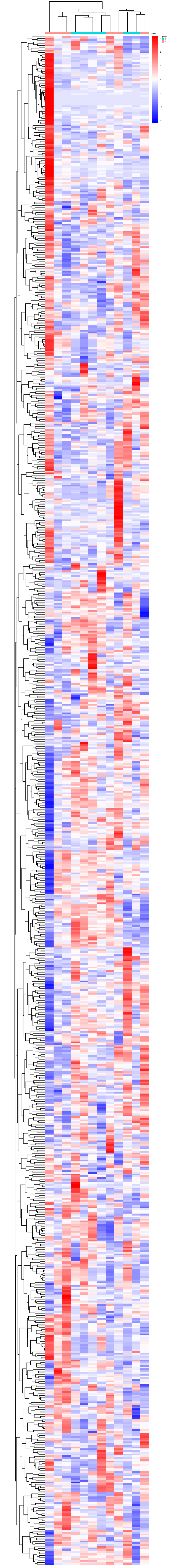

Supplement: S1 File — (ZIP) [file pone.0325562.s001.zip › S1_File/Metabolomic analysis/Hierarchical Clustering Analysis/TOTAL-heatmap no label.pdf]

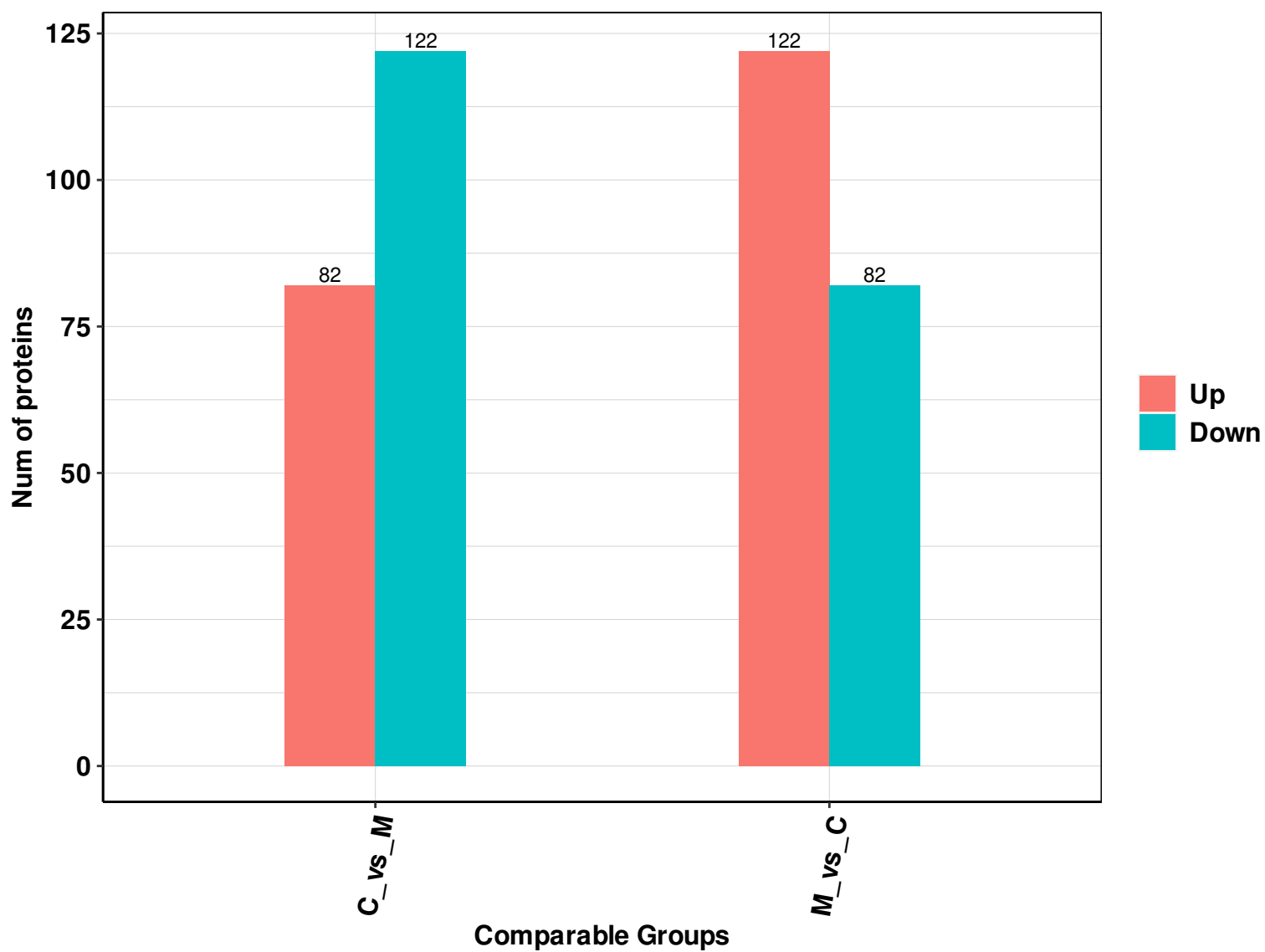

Supplement: S1 File — (ZIP) [file pone.0325562.s001.zip › S1_File/Proteomics analysis/Statistics Analysis/4_Differentially Expressed Proteins.pdf]

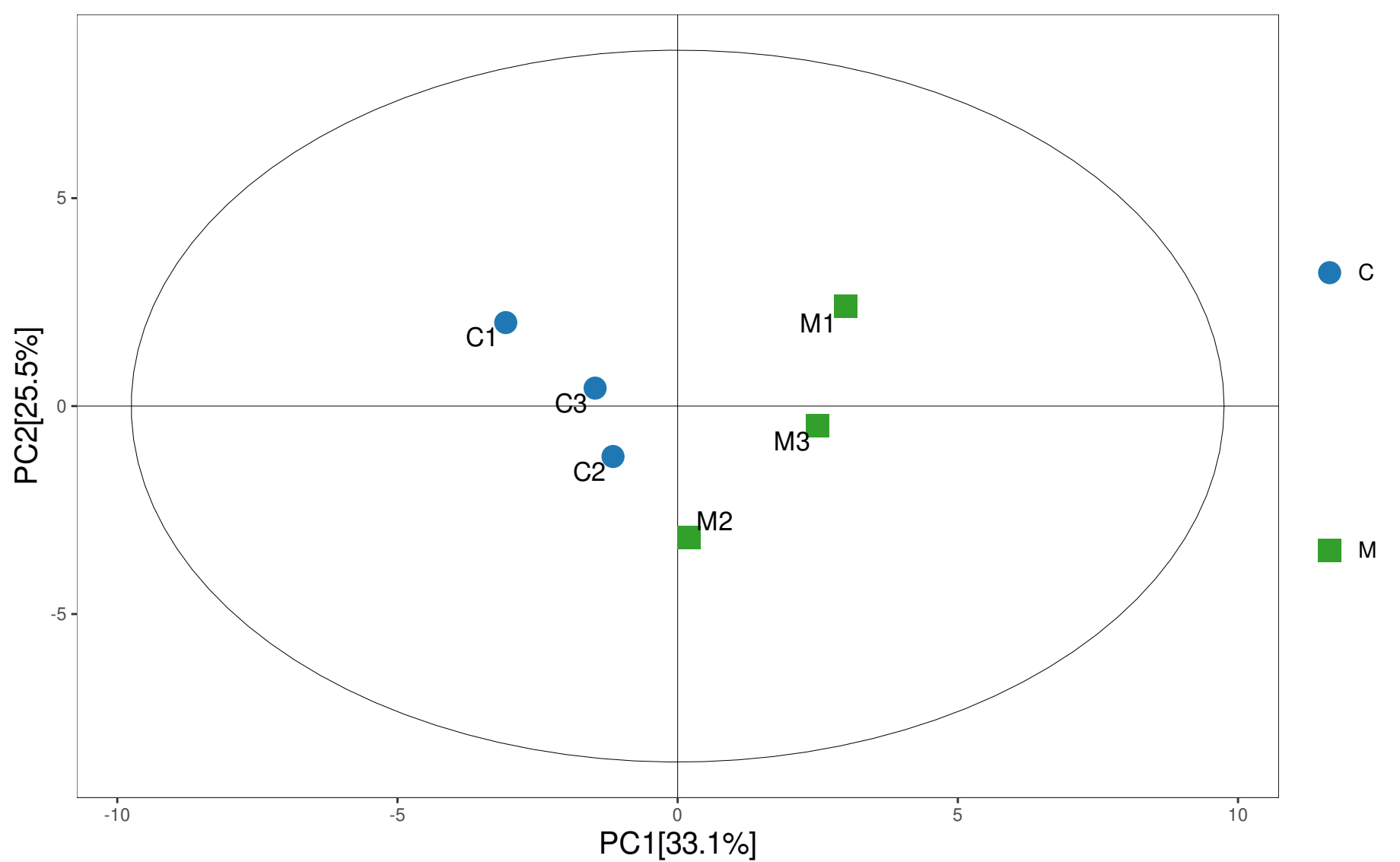

Supplement: S1 File — (ZIP) [file pone.0325562.s001.zip › S1_File/Proteomics analysis/PCA Analysis/score plot.pdf]

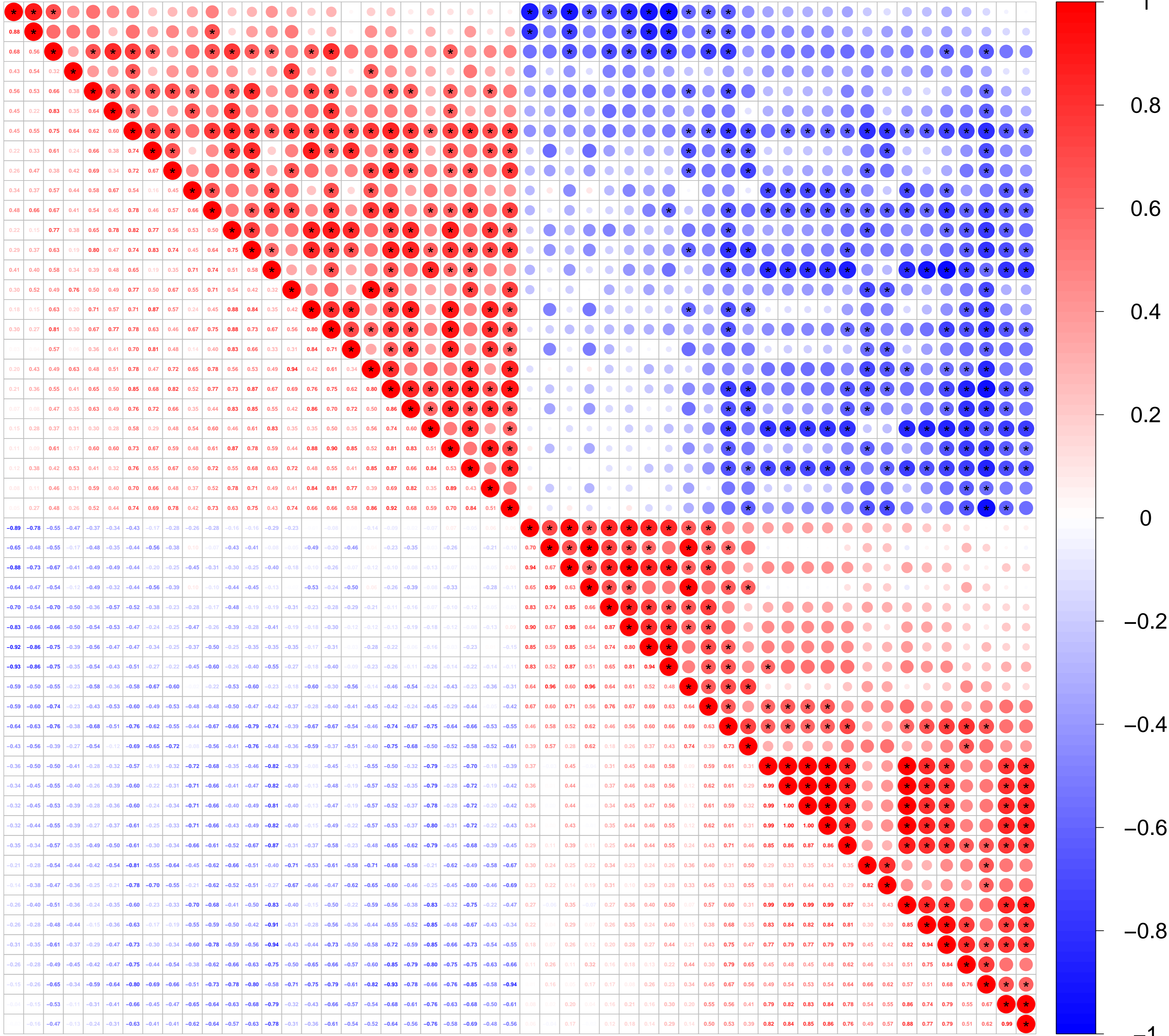

Supplement: S1 File — (ZIP) [file pone.0325562.s001.zip › S1_File/Metabolomic analysis/Correlation Analysis/C-M/Correlation plot no label.pdf]

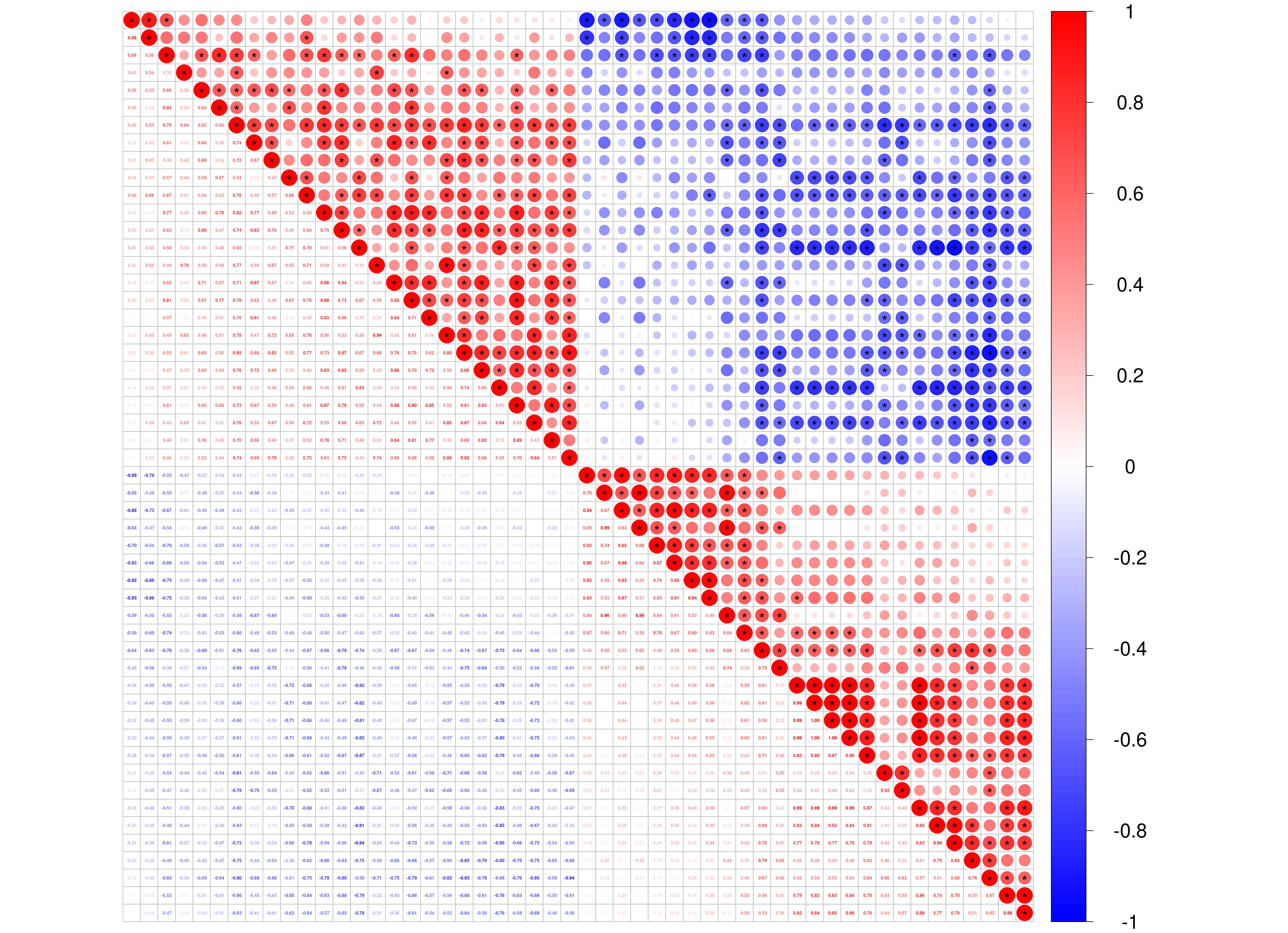

Supplement: S1 File — (ZIP) [file pone.0325562.s001.zip › S1_File/Metabolomic analysis/Correlation Analysis/C-M/Correlation plot no label.jpg]

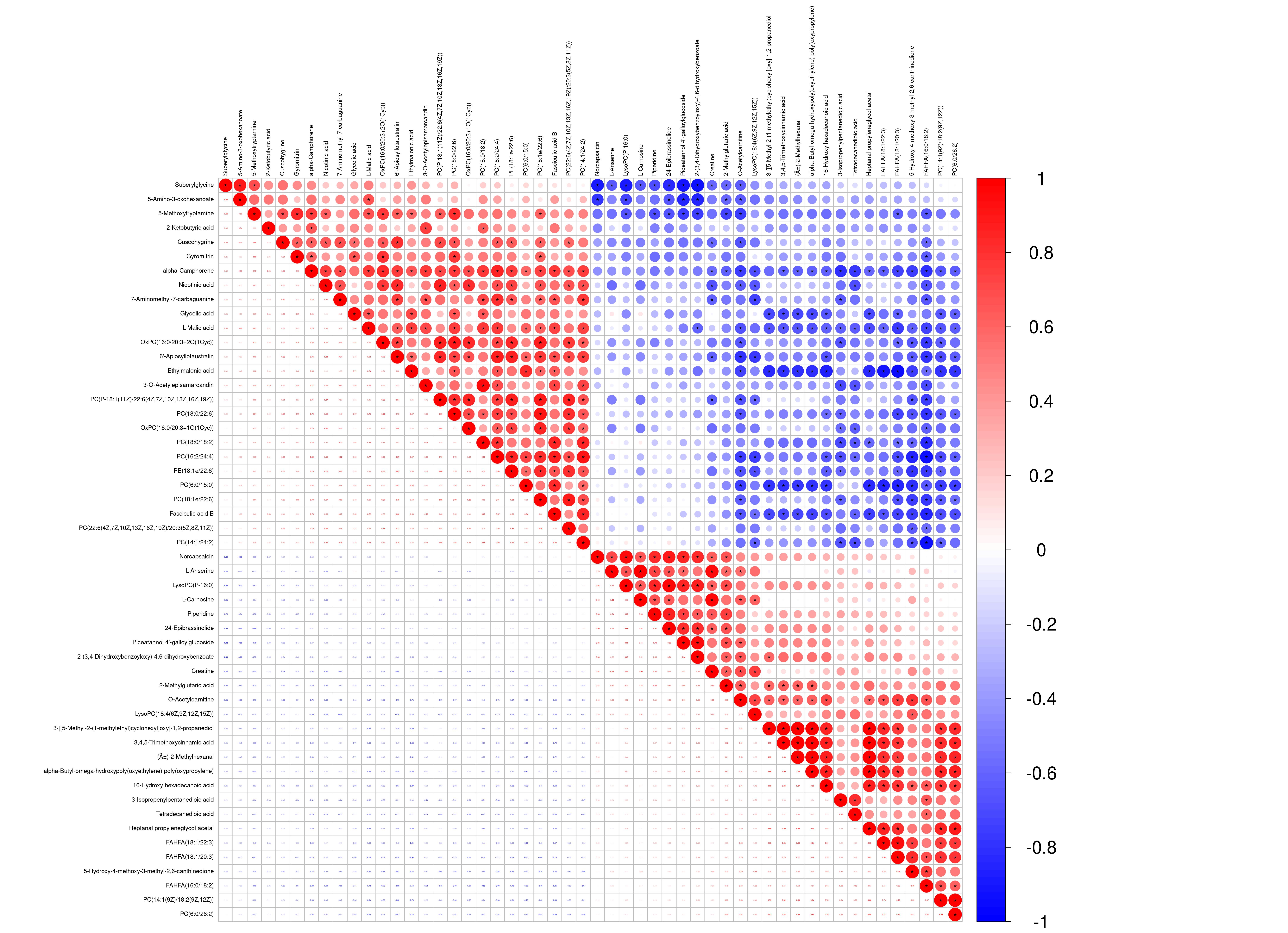

Supplement: S1 File — (ZIP) [file pone.0325562.s001.zip › S1_File/Metabolomic analysis/Correlation Analysis/C-M/Correlation plot.jpg]

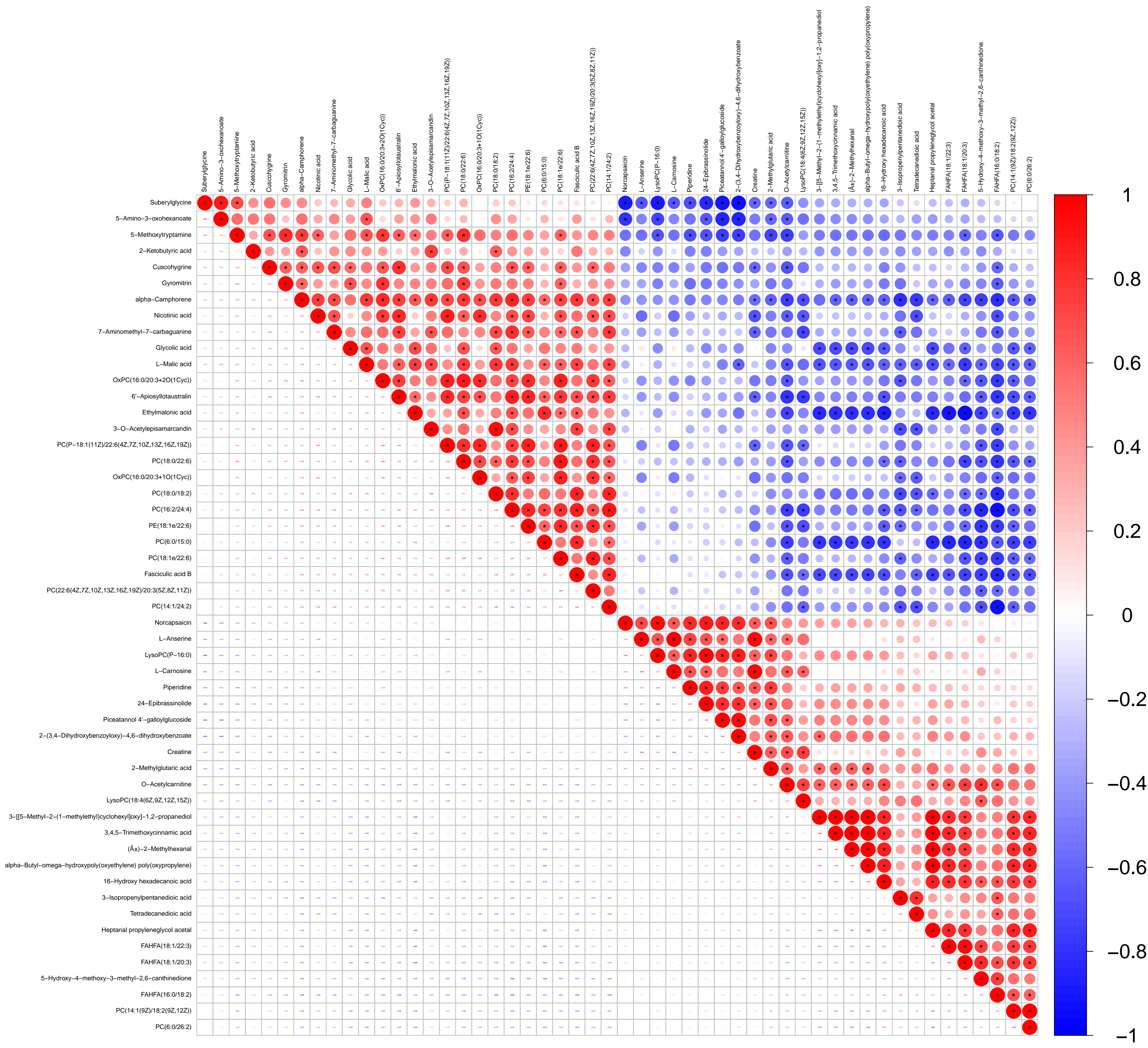

Supplement: S1 File — (ZIP) [file pone.0325562.s001.zip › S1_File/Metabolomic analysis/Correlation Analysis/C-M/Correlation plot.pdf]

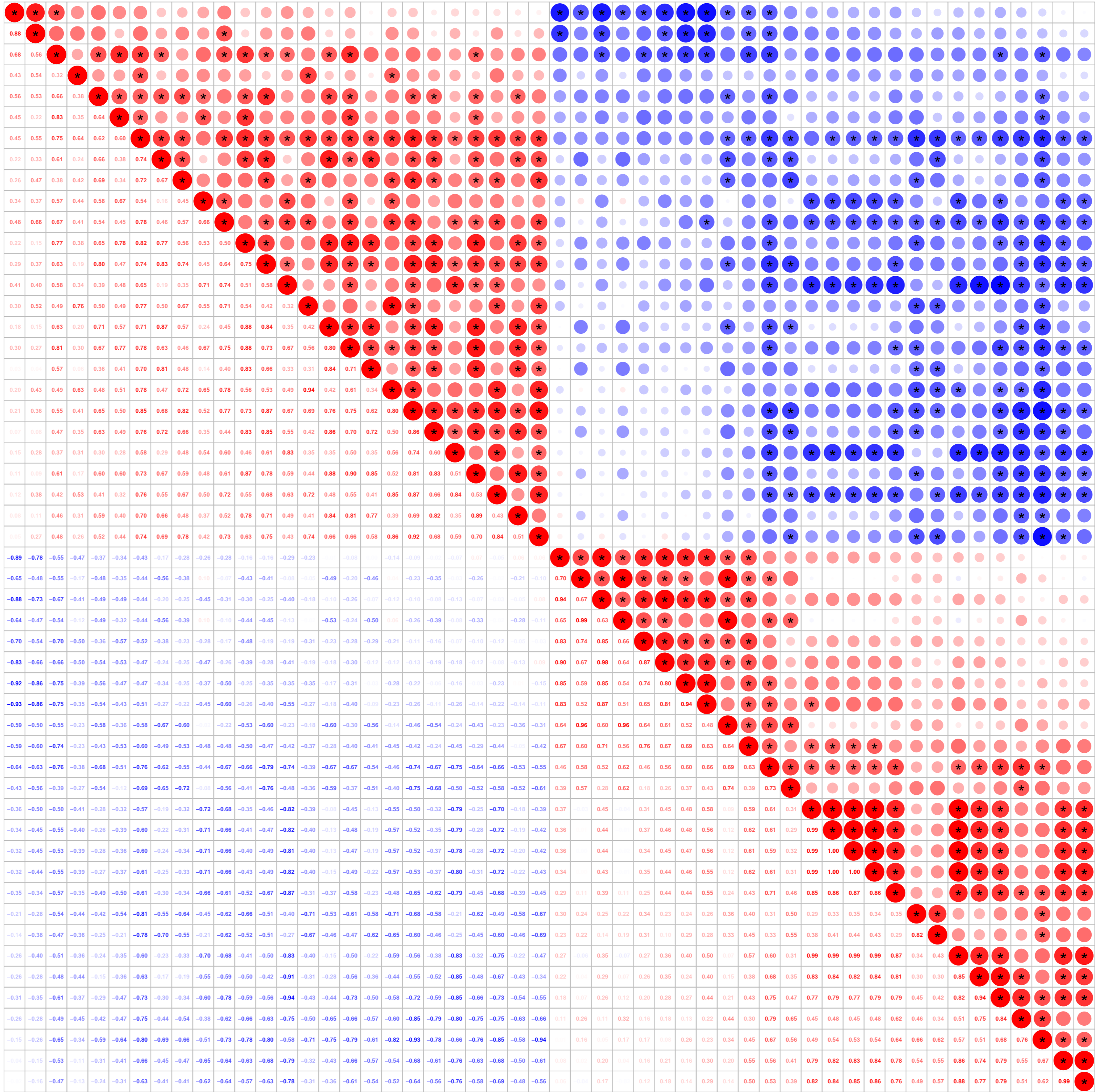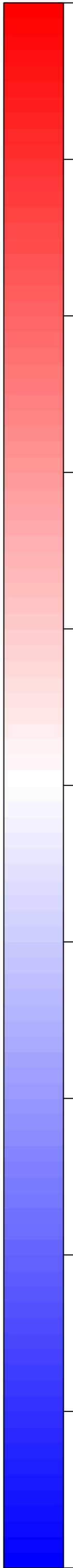

1  
0.8  
0.6  
0.4  
0.2  
0  
-0.2  
-0.4  
-0.6  
-0.8  
-1

Supplement: S1 File — (ZIP) [file pone.0325562.s001.zip › S1_File/Metabolomic analysis/Correlation Analysis/M-C/Correlation plot no label.pdf]

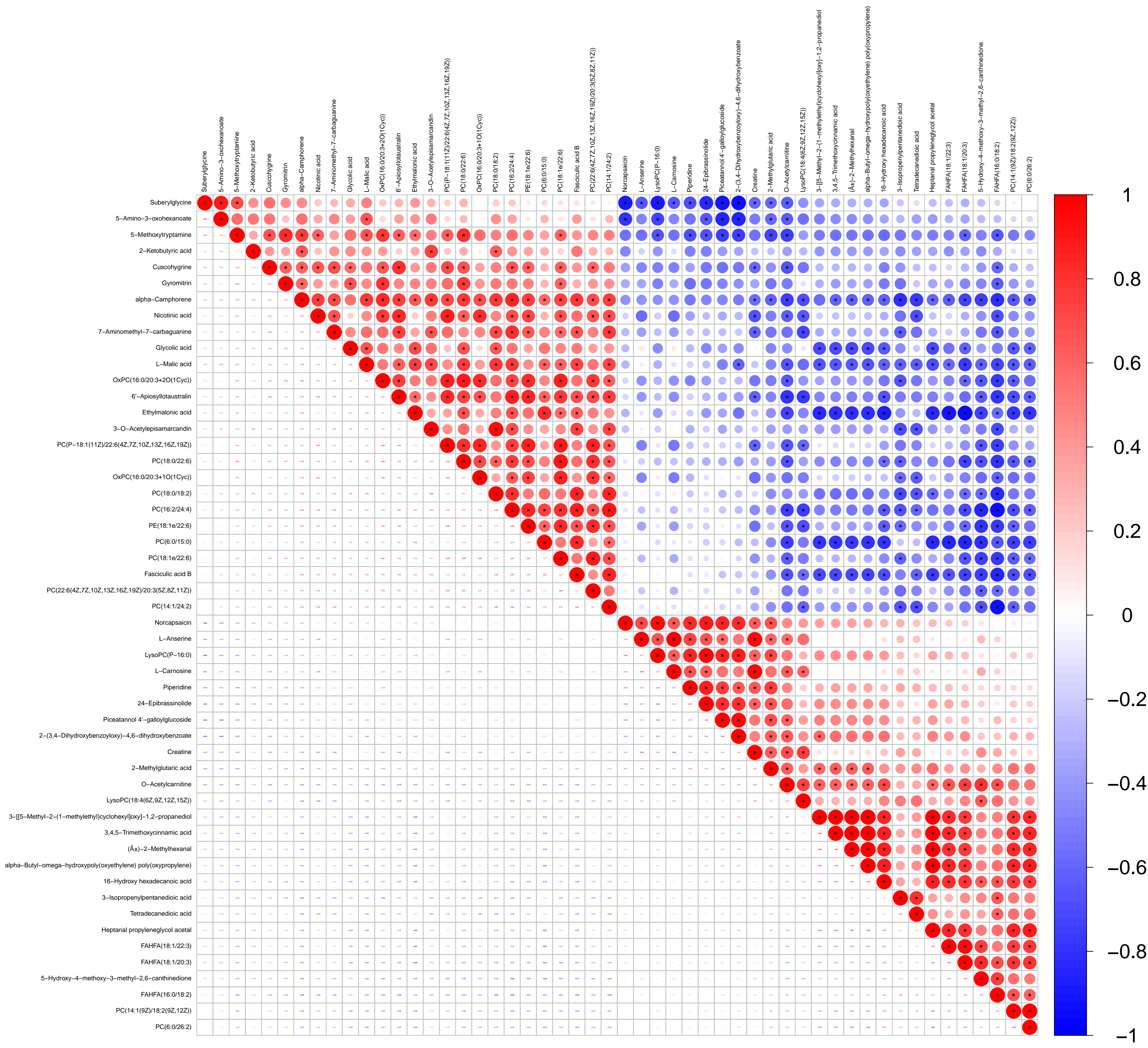

Supplement: S1 File — (ZIP) [file pone.0325562.s001.zip › S1_File/Metabolomic analysis/Correlation Analysis/M-C/Correlation plot.pdf]

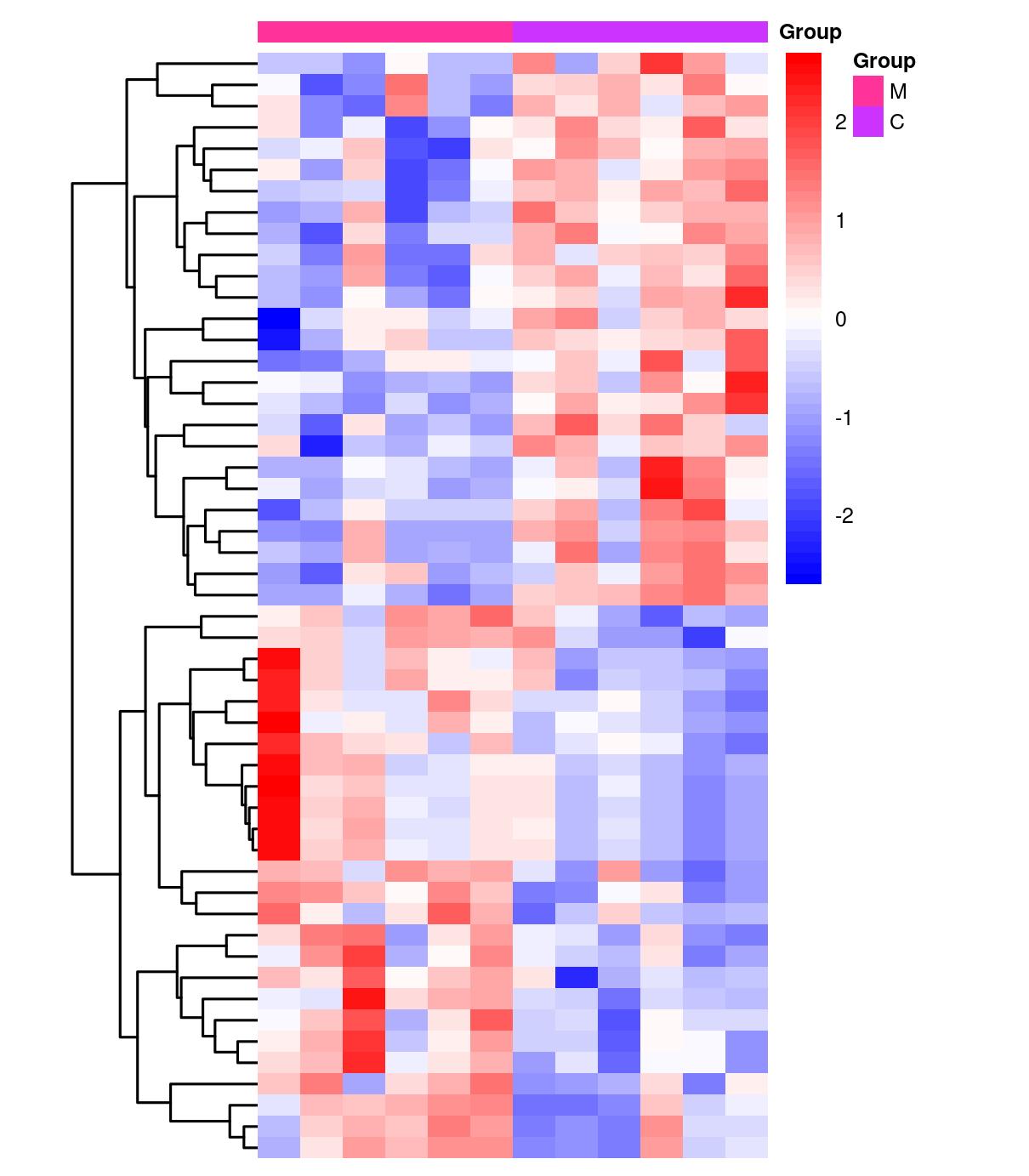

Supplement: S1 File — (ZIP) [file pone.0325562.s001.zip › S1_File/Metabolomic analysis/Hierarchical Clustering Analysis/C-M/heatmap no label.jpg]

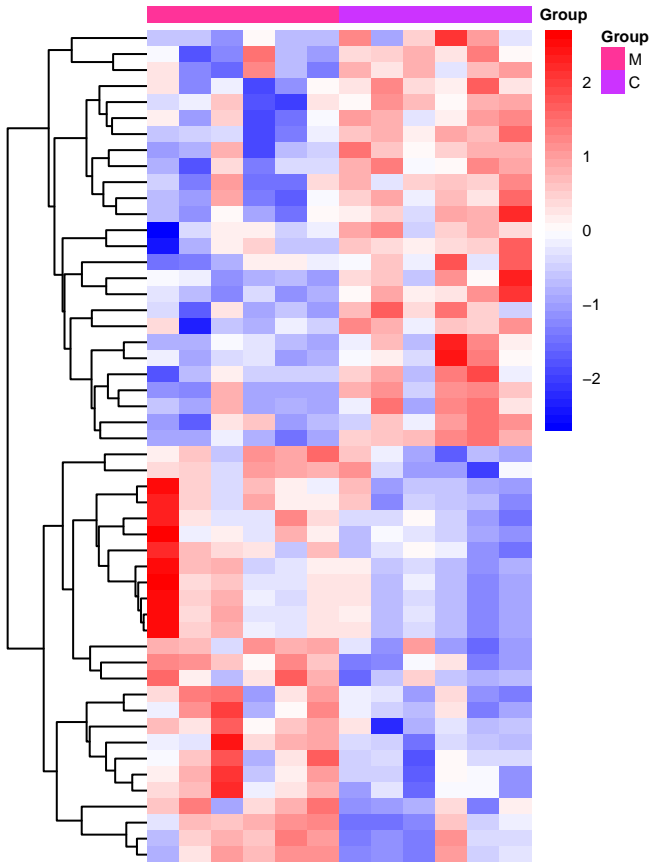

Supplement: S1 File — (ZIP) [file pone.0325562.s001.zip › S1_File/Metabolomic analysis/Hierarchical Clustering Analysis/C-M/heatmap no label.pdf]

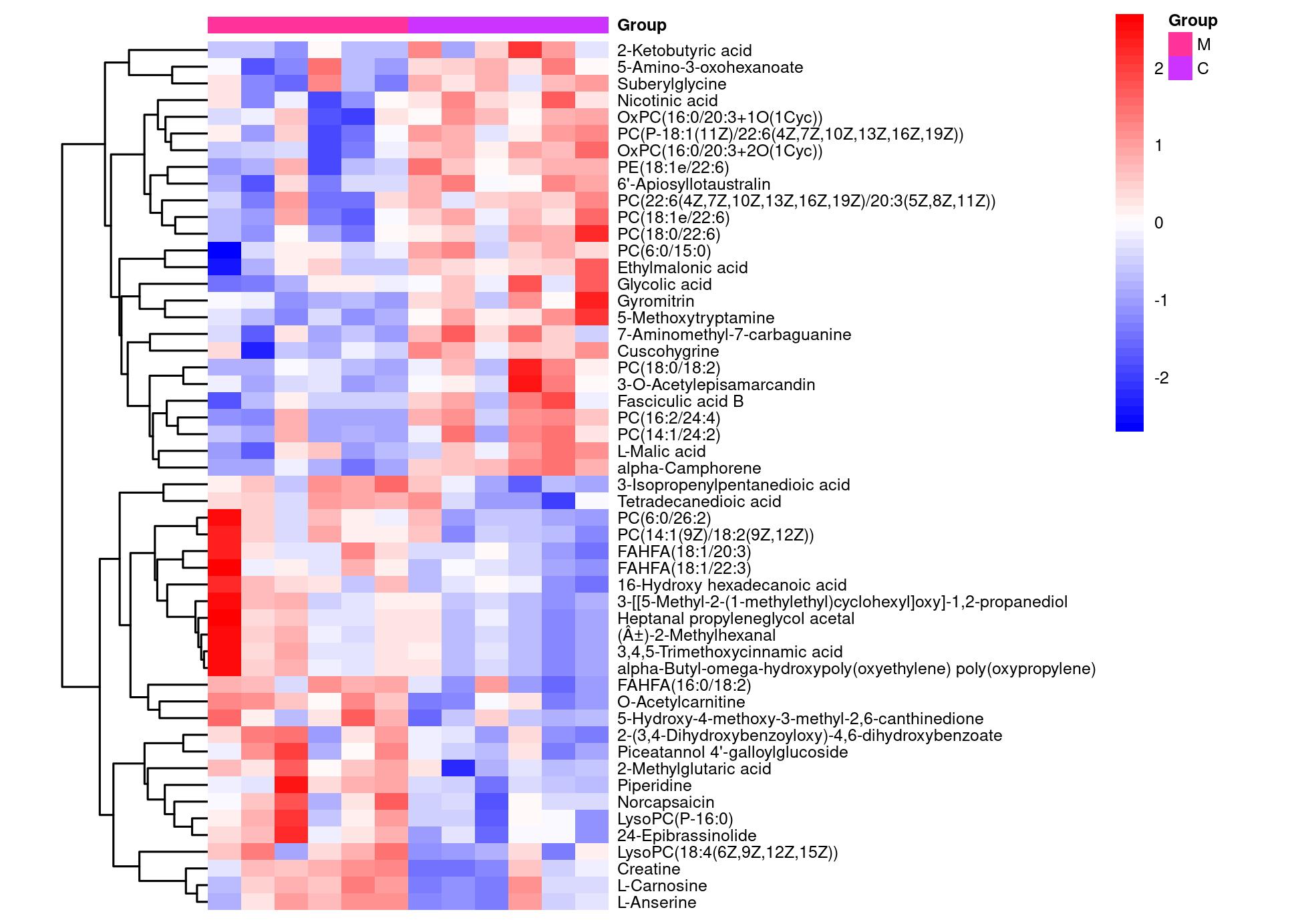

Supplement: S1 File — (ZIP) [file pone.0325562.s001.zip › S1_File/Metabolomic analysis/Hierarchical Clustering Analysis/C-M/heatmap.jpg]

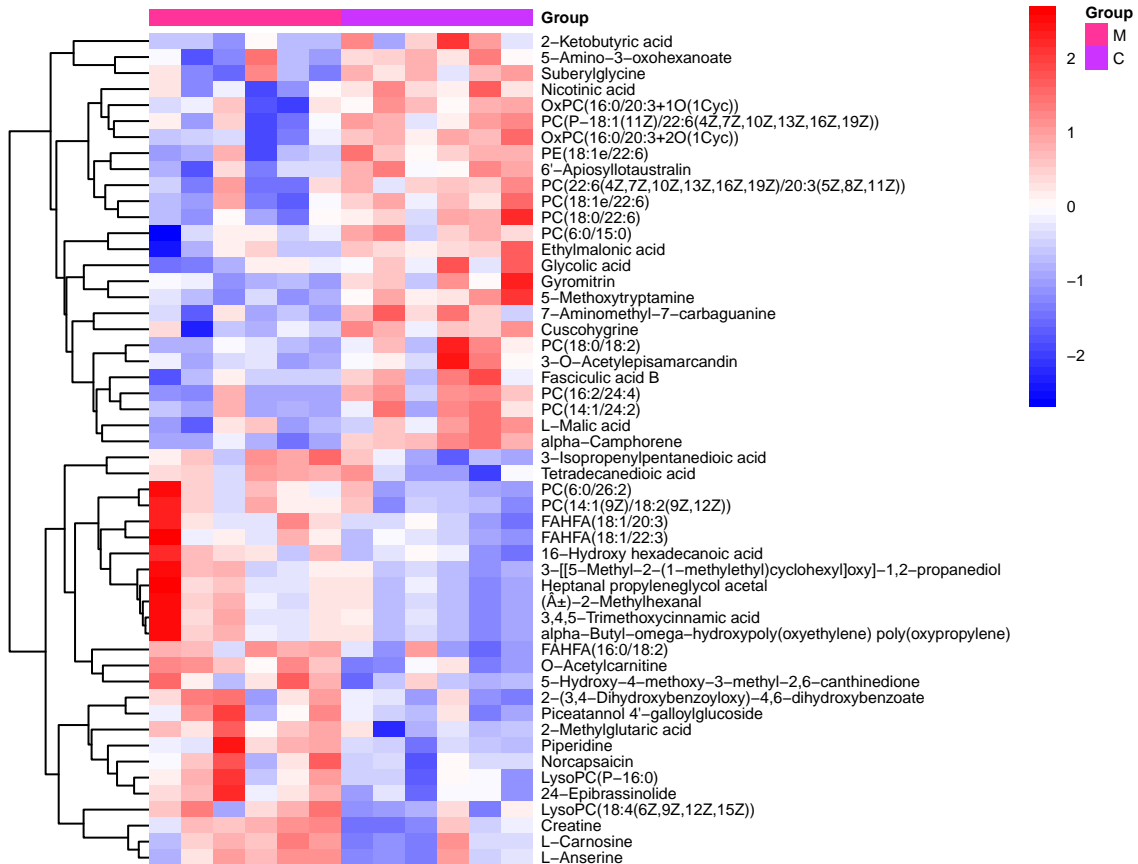

Supplement: S1 File — (ZIP) [file pone.0325562.s001.zip › S1_File/Metabolomic analysis/Hierarchical Clustering Analysis/C-M/heatmap.pdf]

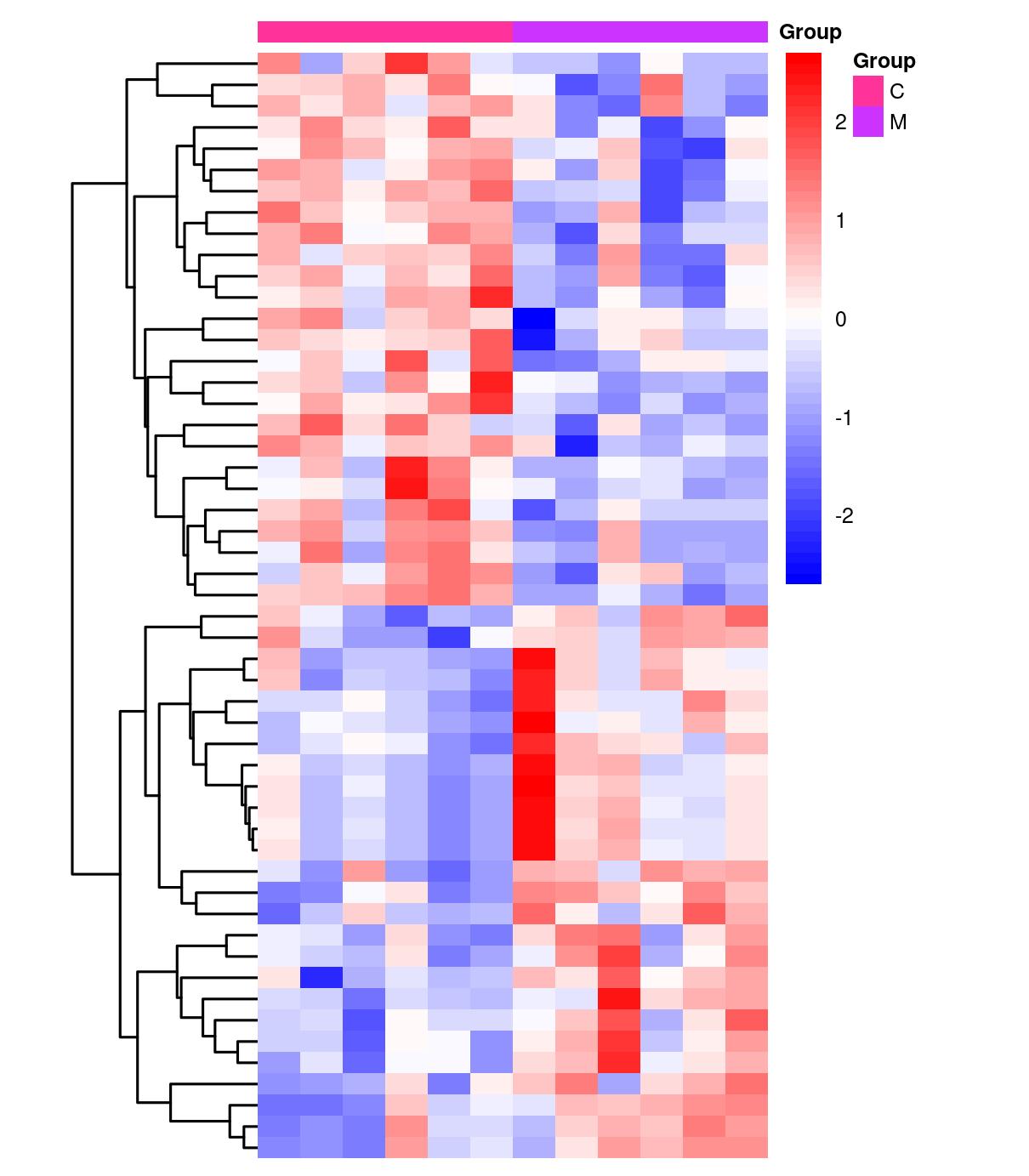

Supplement: S1 File — (ZIP) [file pone.0325562.s001.zip › S1_File/Metabolomic analysis/Hierarchical Clustering Analysis/M-C/heatmap no label.jpg]

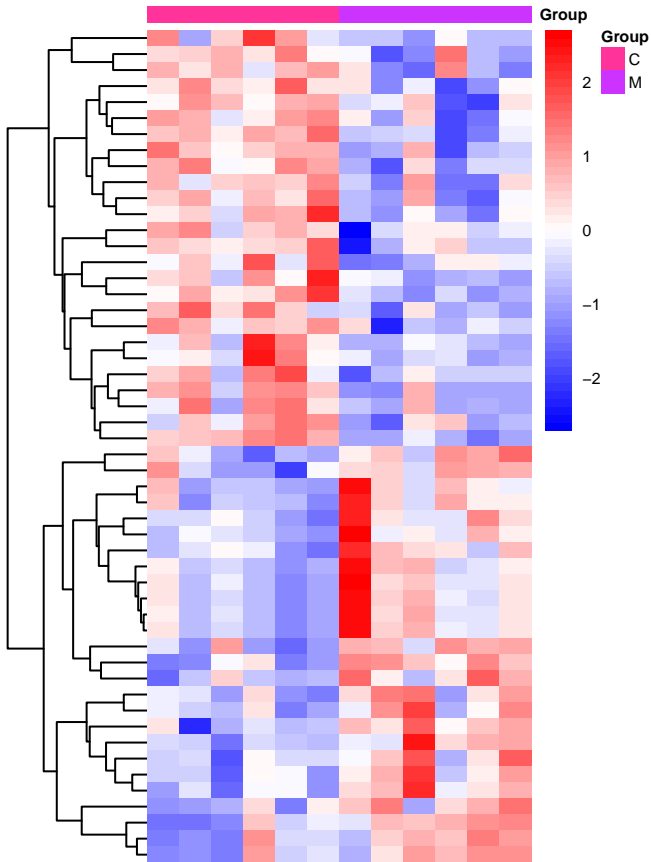

Supplement: S1 File — (ZIP) [file pone.0325562.s001.zip › S1_File/Metabolomic analysis/Hierarchical Clustering Analysis/M-C/heatmap no label.pdf]

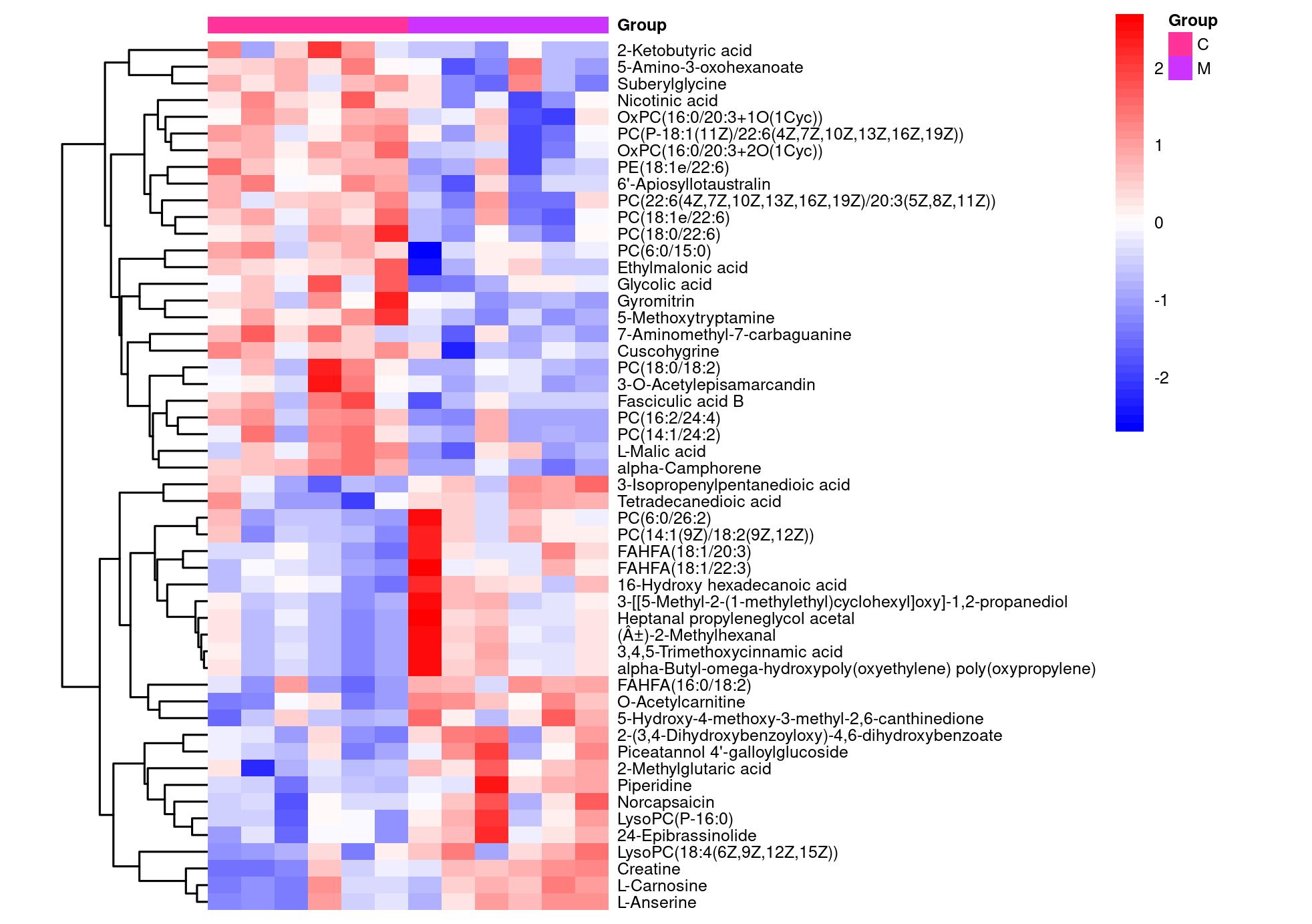

Supplement: S1 File — (ZIP) [file pone.0325562.s001.zip › S1_File/Metabolomic analysis/Hierarchical Clustering Analysis/M-C/heatmap.jpg]

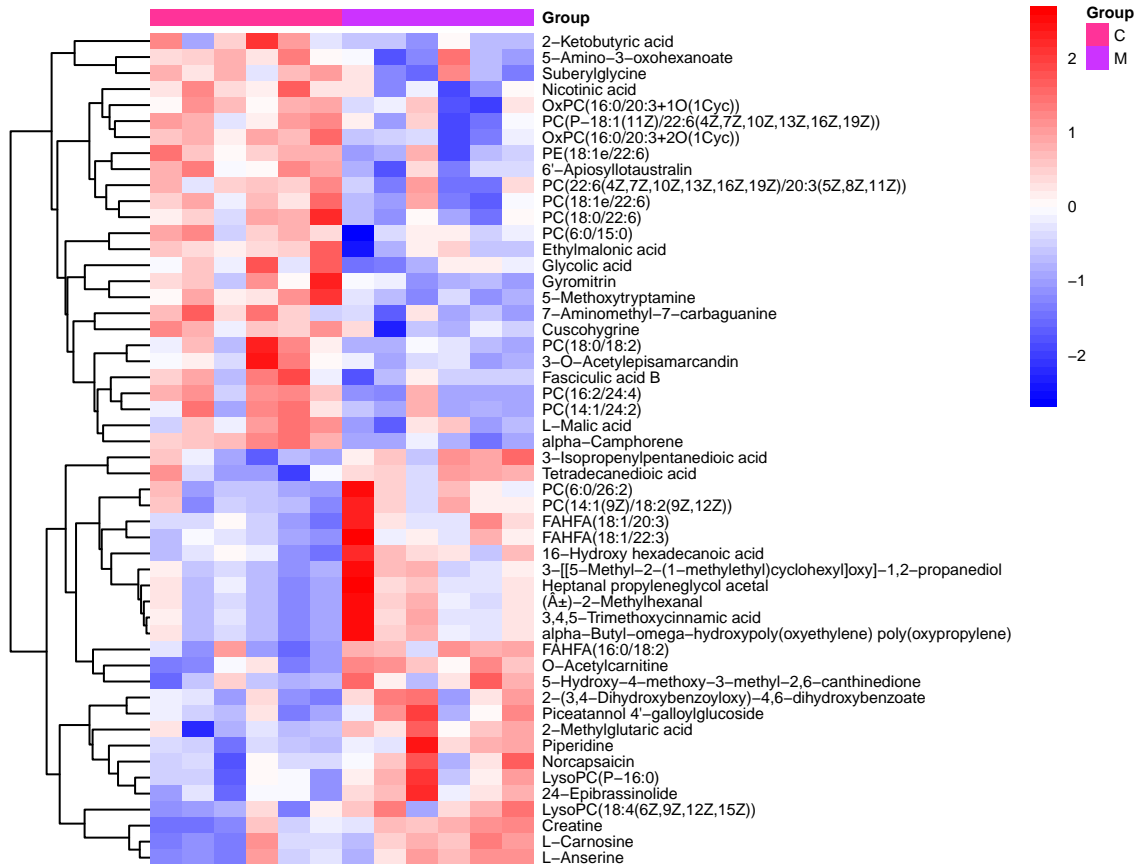

Supplement: S1 File — (ZIP) [file pone.0325562.s001.zip › S1_File/Metabolomic analysis/Hierarchical Clustering Analysis/M-C/heatmap.pdf]

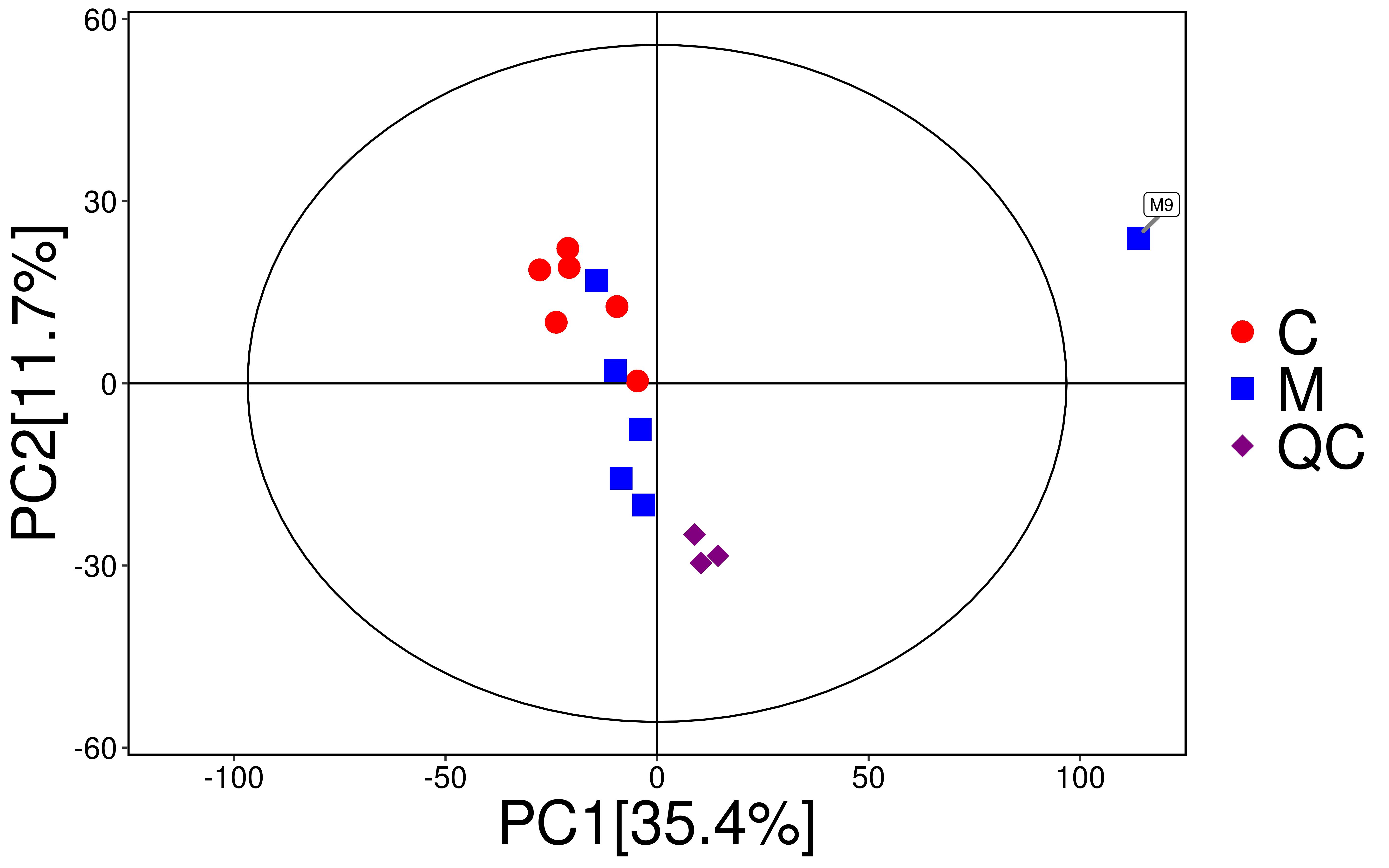

Supplement: S1 File — (ZIP) [file pone.0325562.s001.zip › S1_File/Metabolomic analysis/Statistical Analysis/TOTAL with QC/PCA score label plot.jpg]

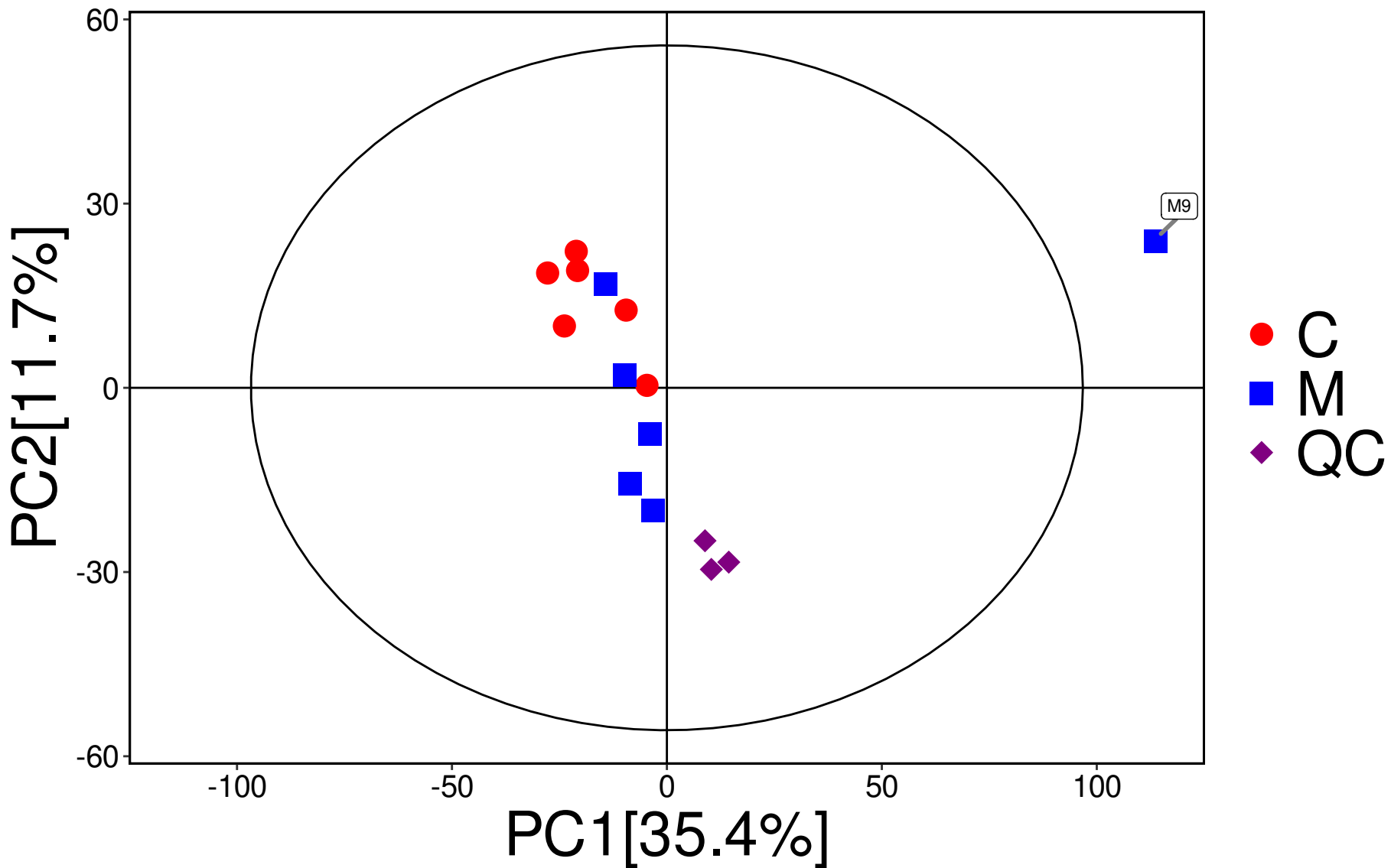

Supplement: S1 File — (ZIP) [file pone.0325562.s001.zip › S1_File/Metabolomic analysis/Statistical Analysis/TOTAL with QC/PCA score label plot.pdf]

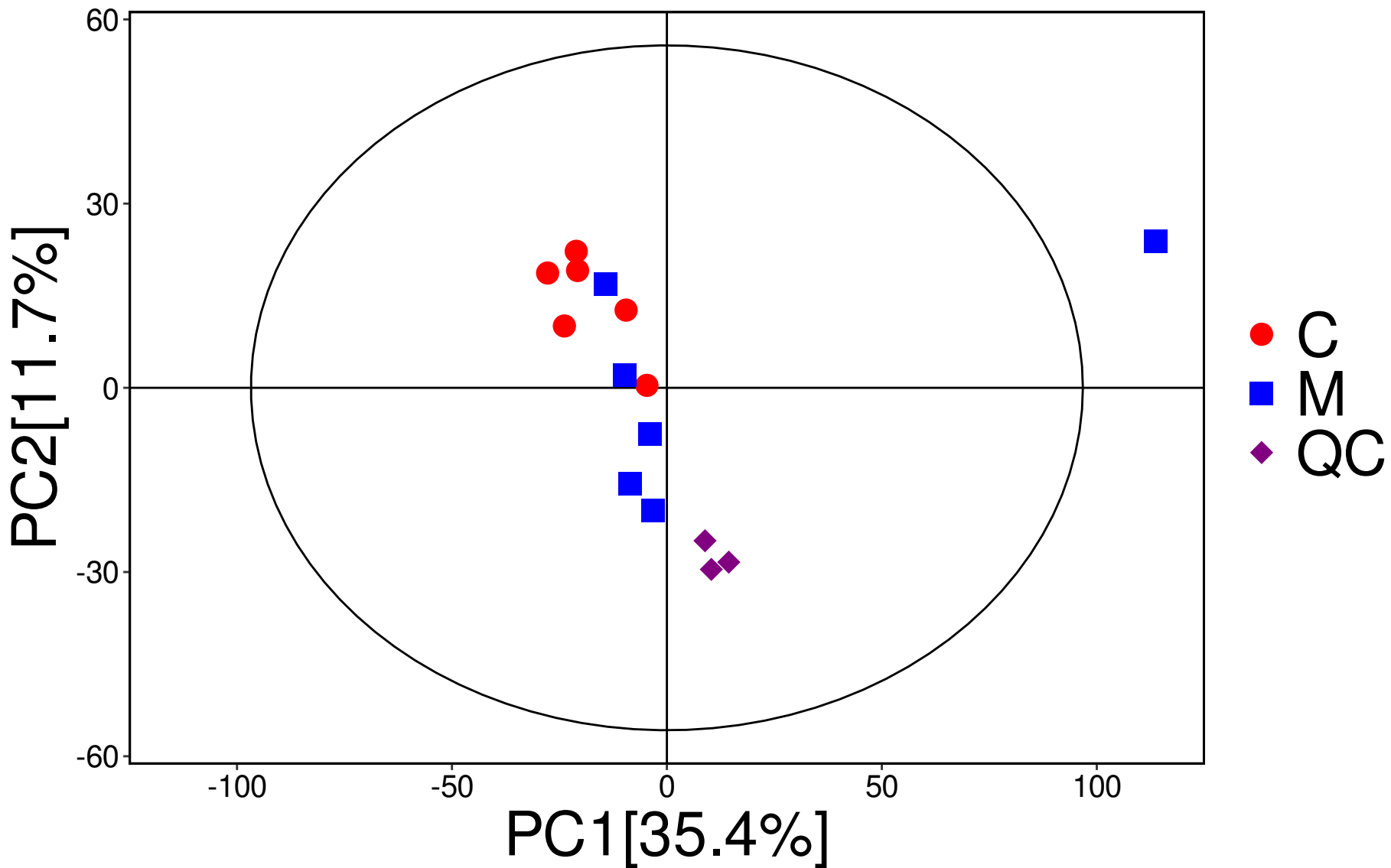

Supplement: S1 File — (ZIP) [file pone.0325562.s001.zip › S1_File/Metabolomic analysis/Statistical Analysis/TOTAL with QC/PCA score plot.pdf]

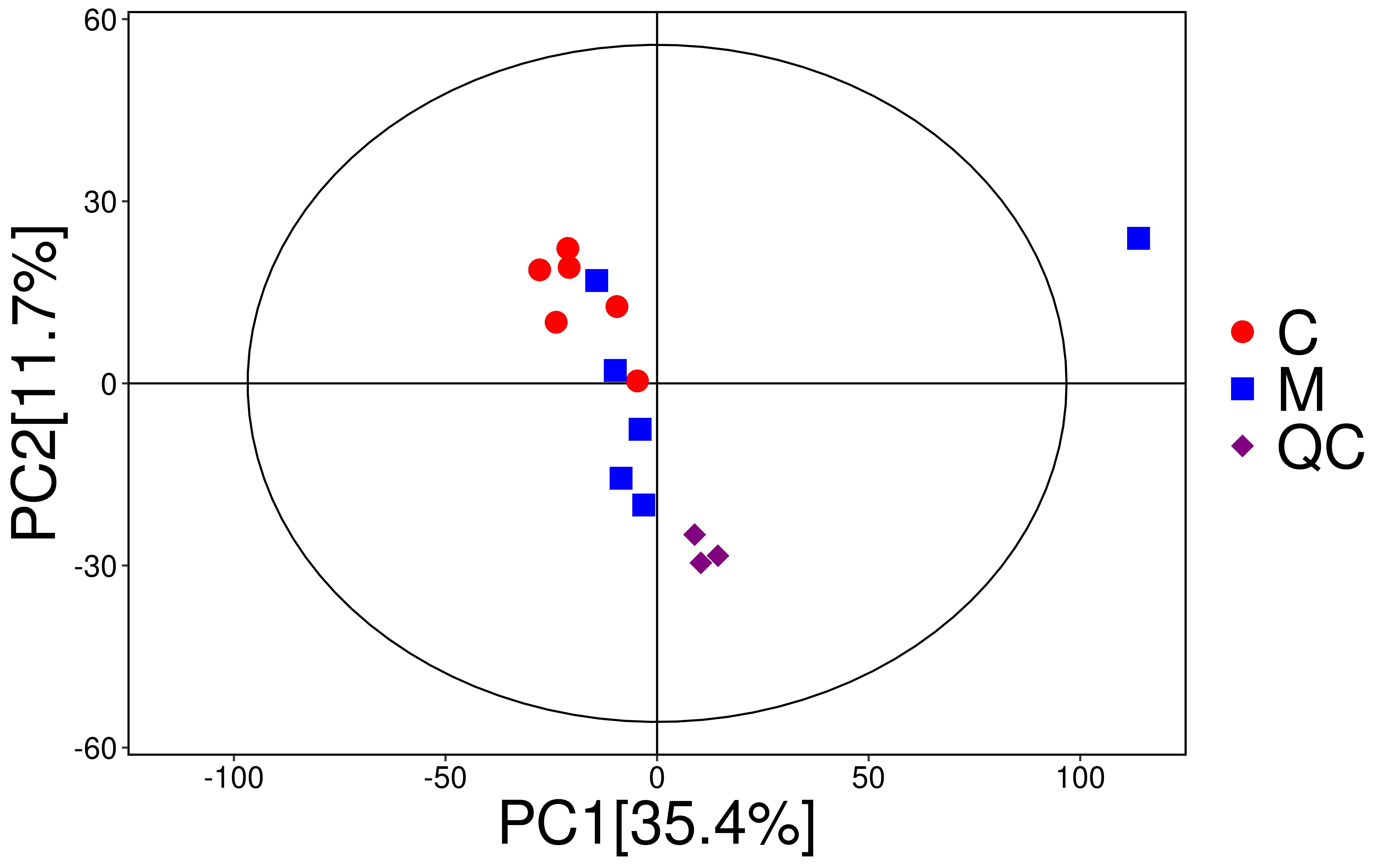

Supplement: S1 File — (ZIP) [file pone.0325562.s001.zip › S1_File/Metabolomic analysis/Statistical Analysis/TOTAL with QC/PCA score plot.jpg]

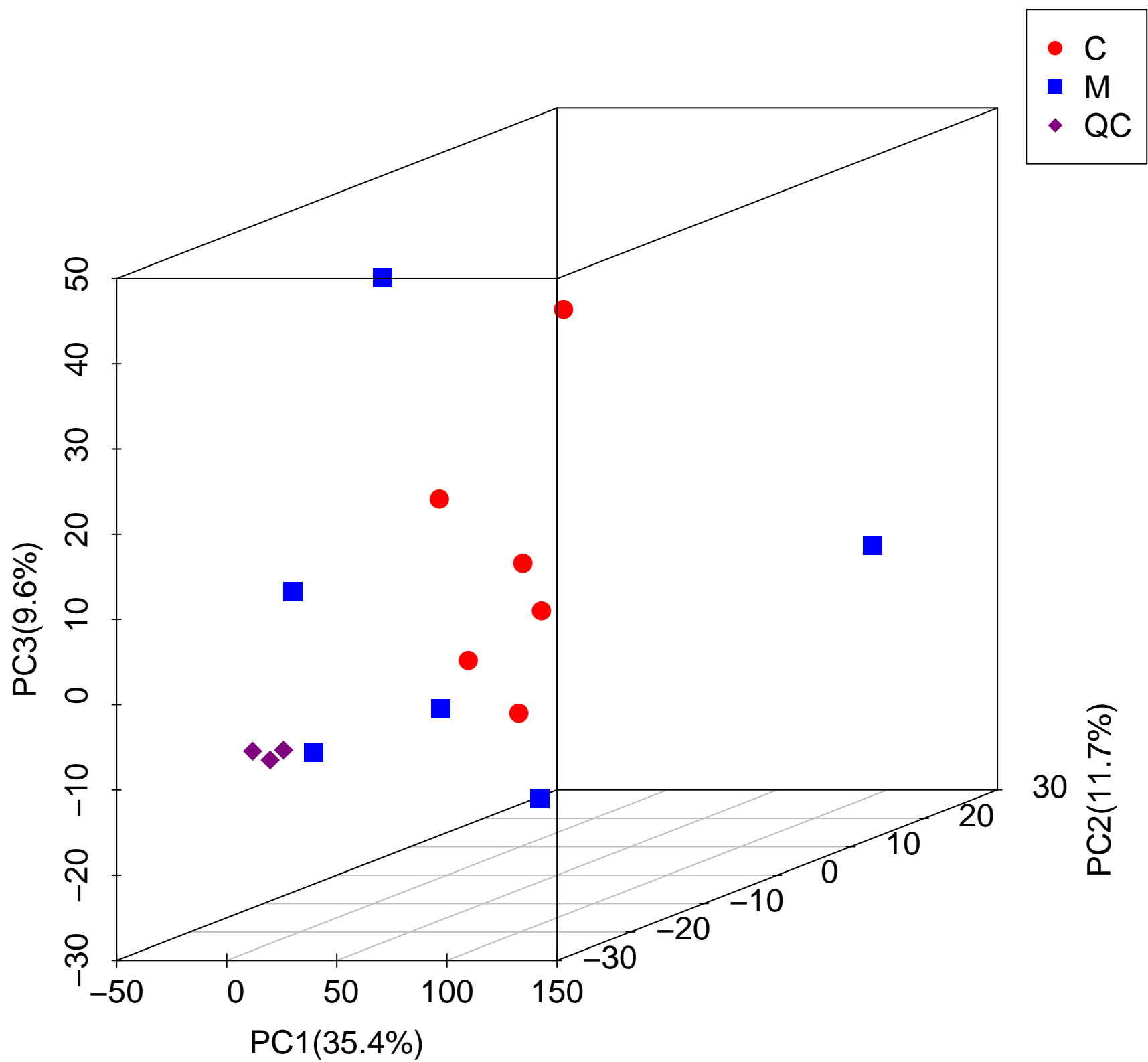

Supplement: S1 File — (ZIP) [file pone.0325562.s001.zip › S1_File/Metabolomic analysis/Statistical Analysis/TOTAL with QC/PCA score plot 3D.pdf]

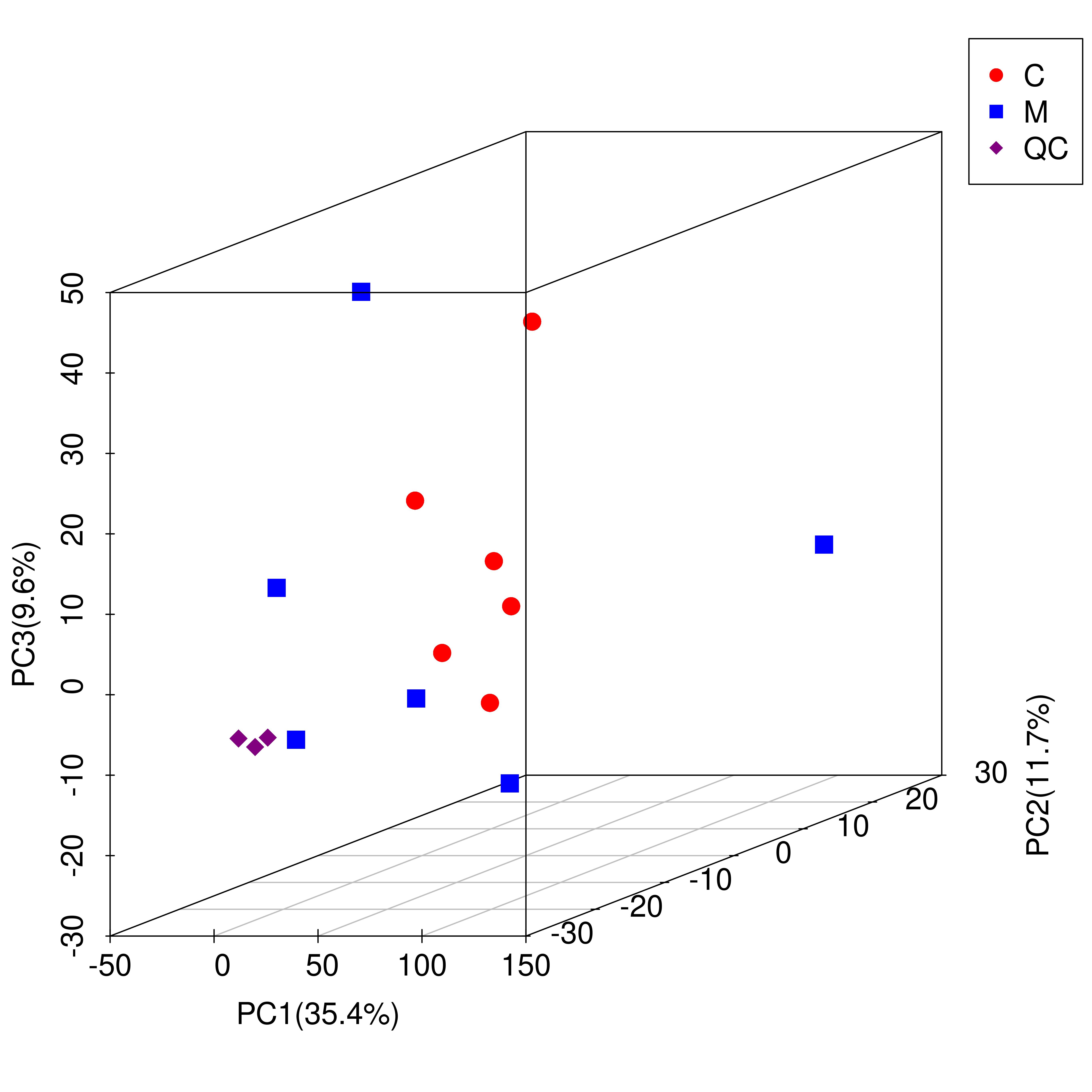

Supplement: S1 File — (ZIP) [file pone.0325562.s001.zip › S1_File/Metabolomic analysis/Statistical Analysis/TOTAL with QC/PCA score plot 3D.jpg]

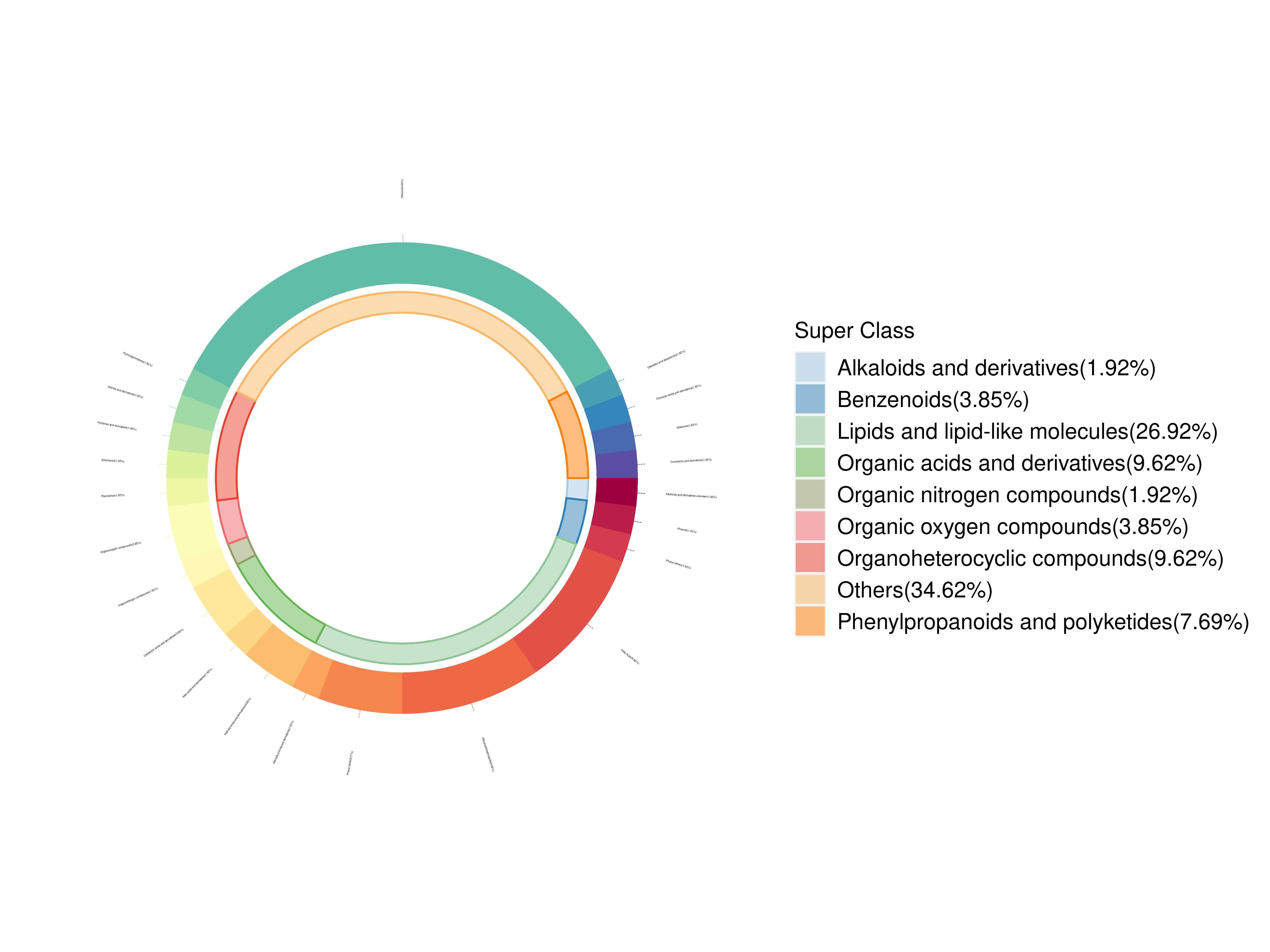

Supplement: S1 File — (ZIP) [file pone.0325562.s001.zip › S1_File/Metabolomic analysis/Statistical Analysis/C-M/DonutPlot.jpg]

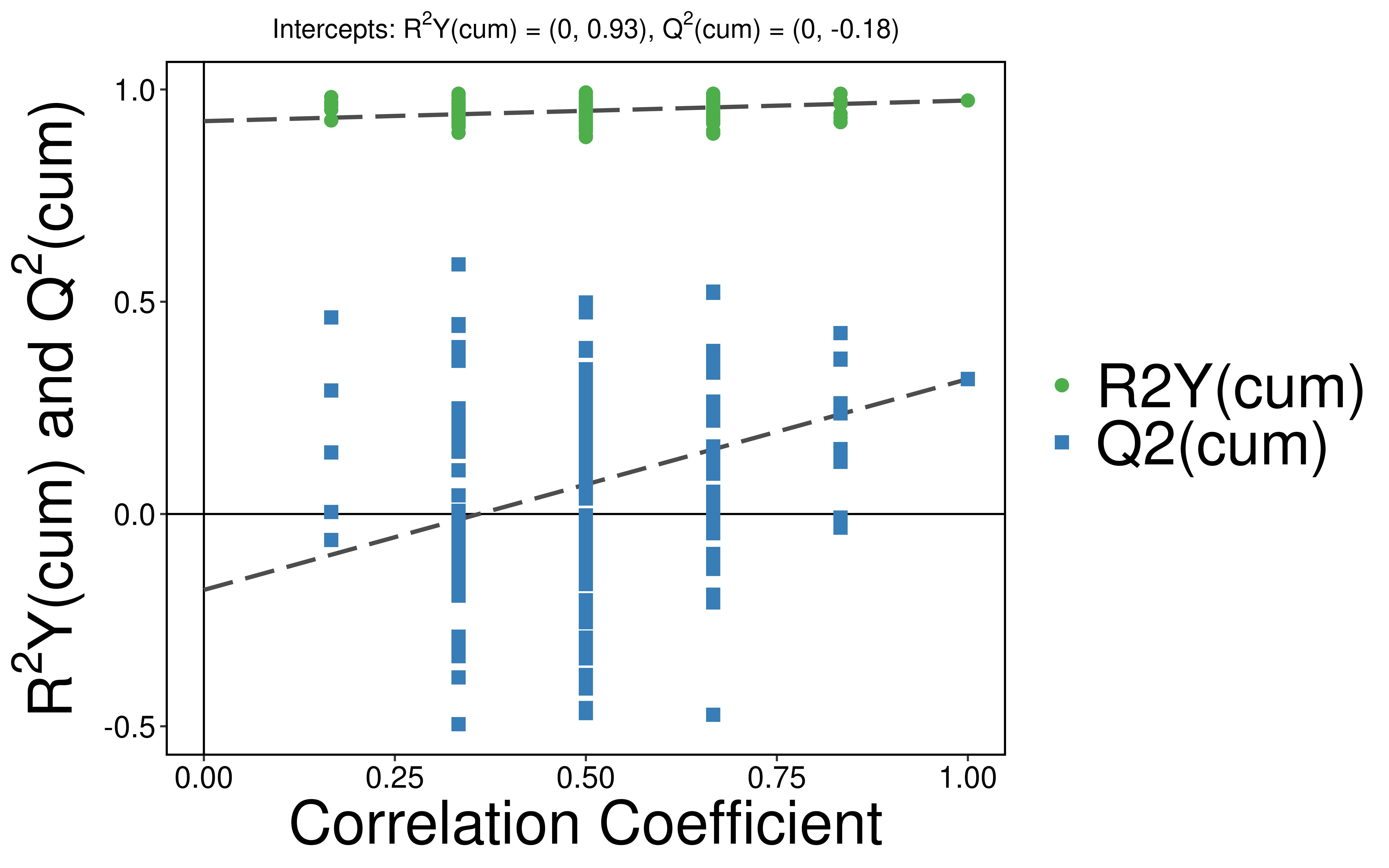

Supplement: S1 File — (ZIP) [file pone.0325562.s001.zip › S1_File/Metabolomic analysis/Statistical Analysis/C-M/OPLS-DA permutation plot.jpg]

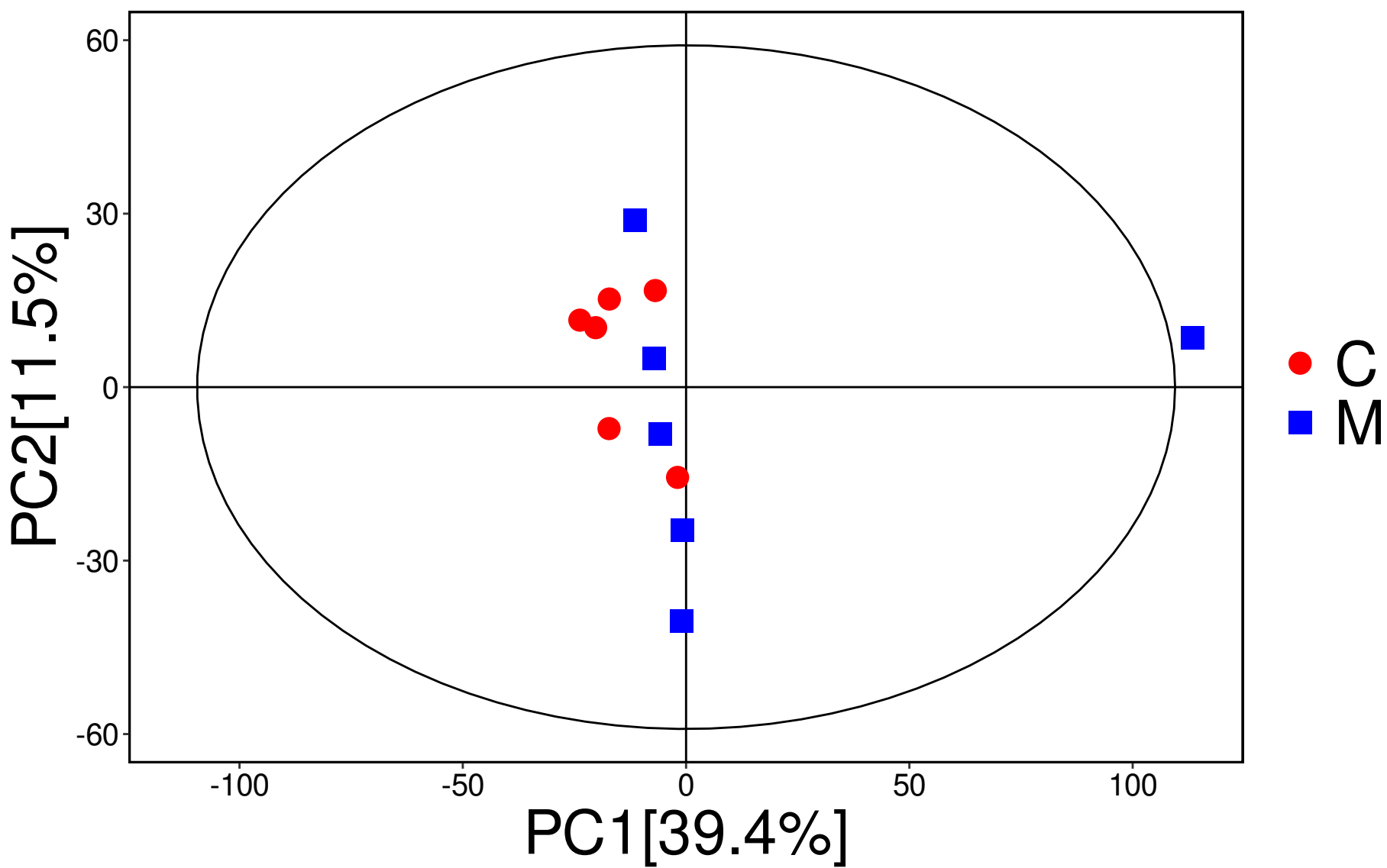

Supplement: S1 File — (ZIP) [file pone.0325562.s001.zip › S1_File/Metabolomic analysis/Statistical Analysis/C-M/PCA score plot.pdf]

Intercepts:  $R^2Y(\text{cum}) = (0, 0.93)$ ,  $Q^2(\text{cum}) = (0, -0.18)$

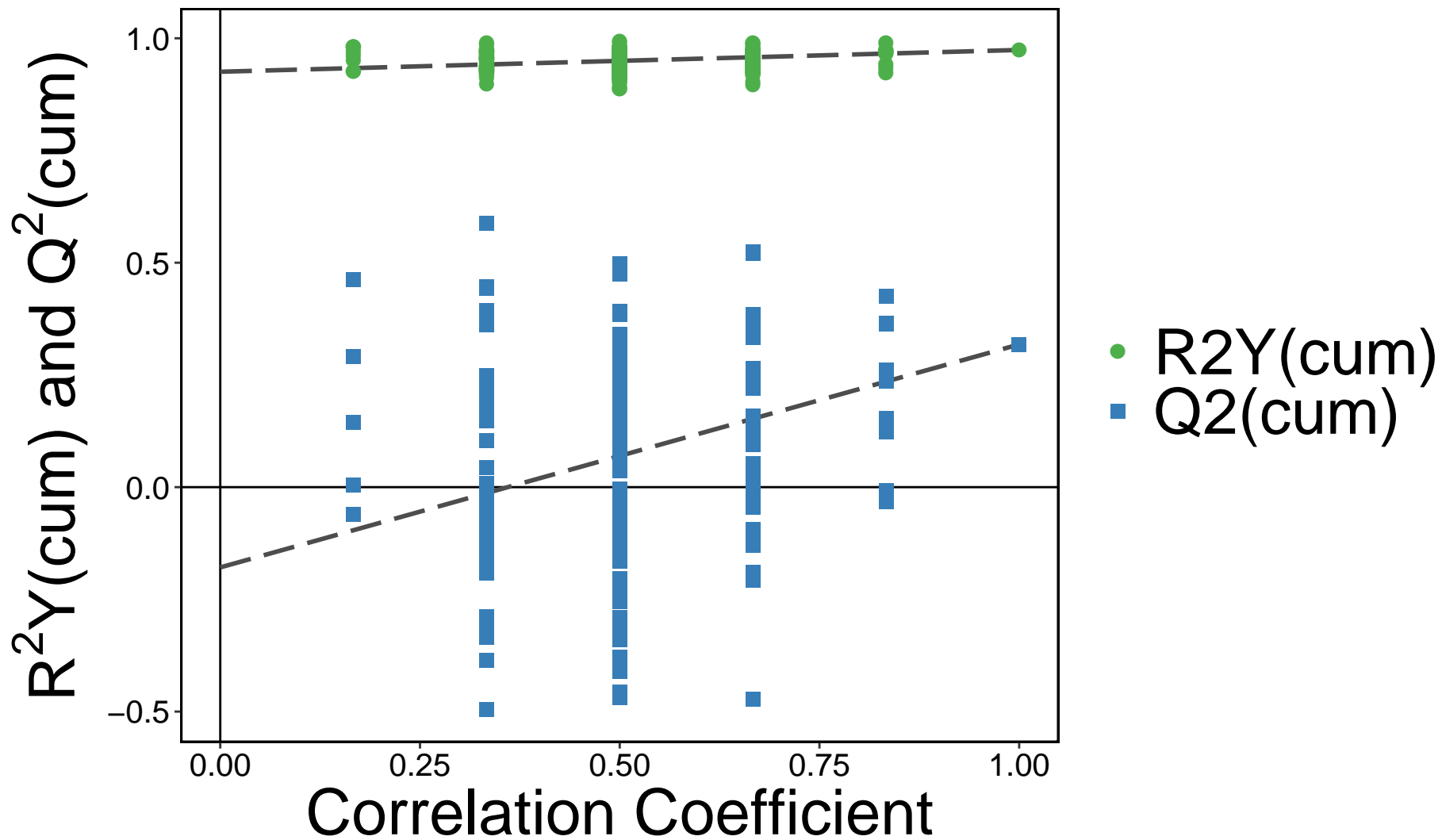

Supplement: S1 File — (ZIP) [file pone.0325562.s001.zip › S1_File/Metabolomic analysis/Statistical Analysis/C-M/OPLS-DA permutation plot.pdf]

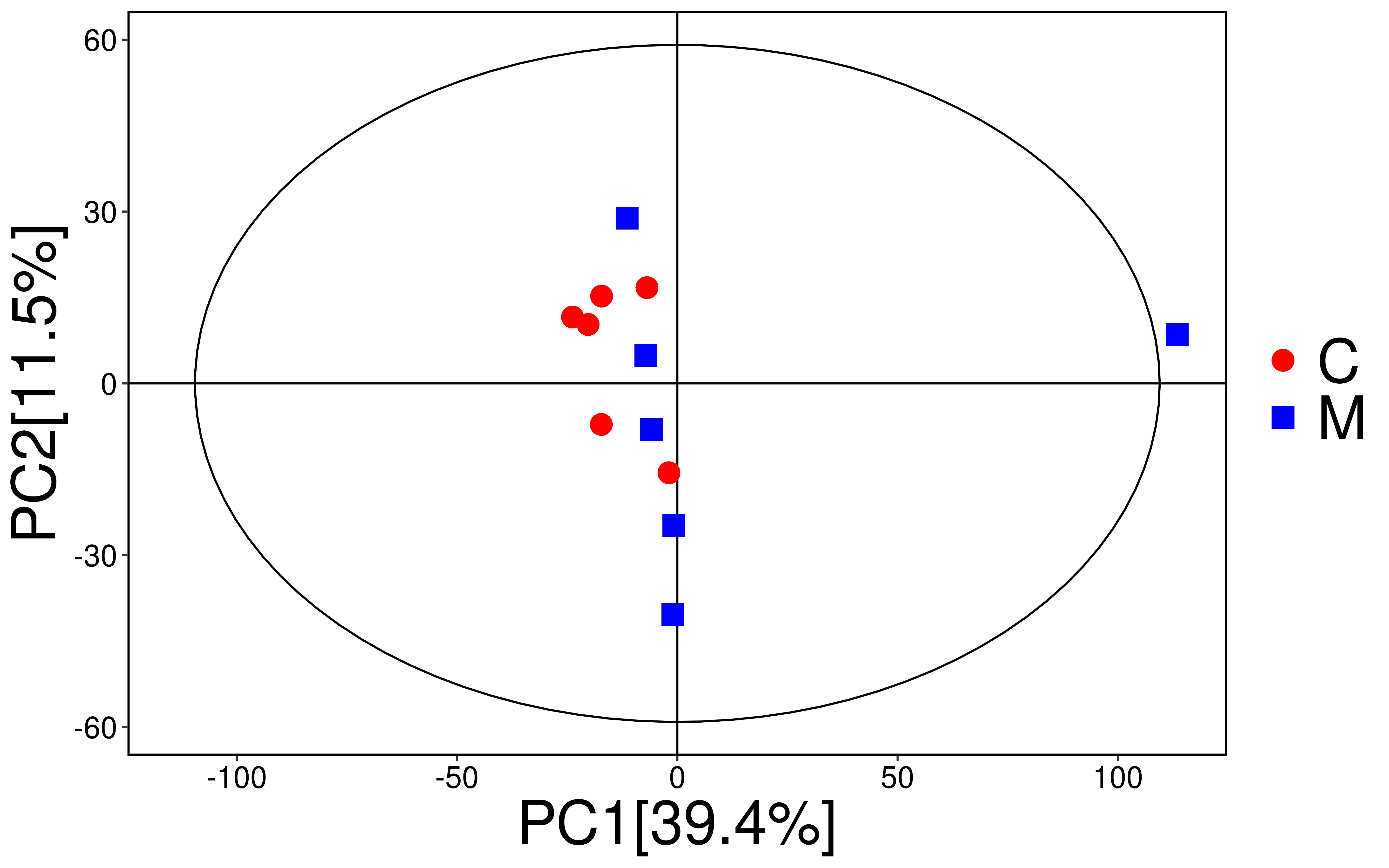

Supplement: S1 File — (ZIP) [file pone.0325562.s001.zip › S1_File/Metabolomic analysis/Statistical Analysis/C-M/PCA score plot.jpg]

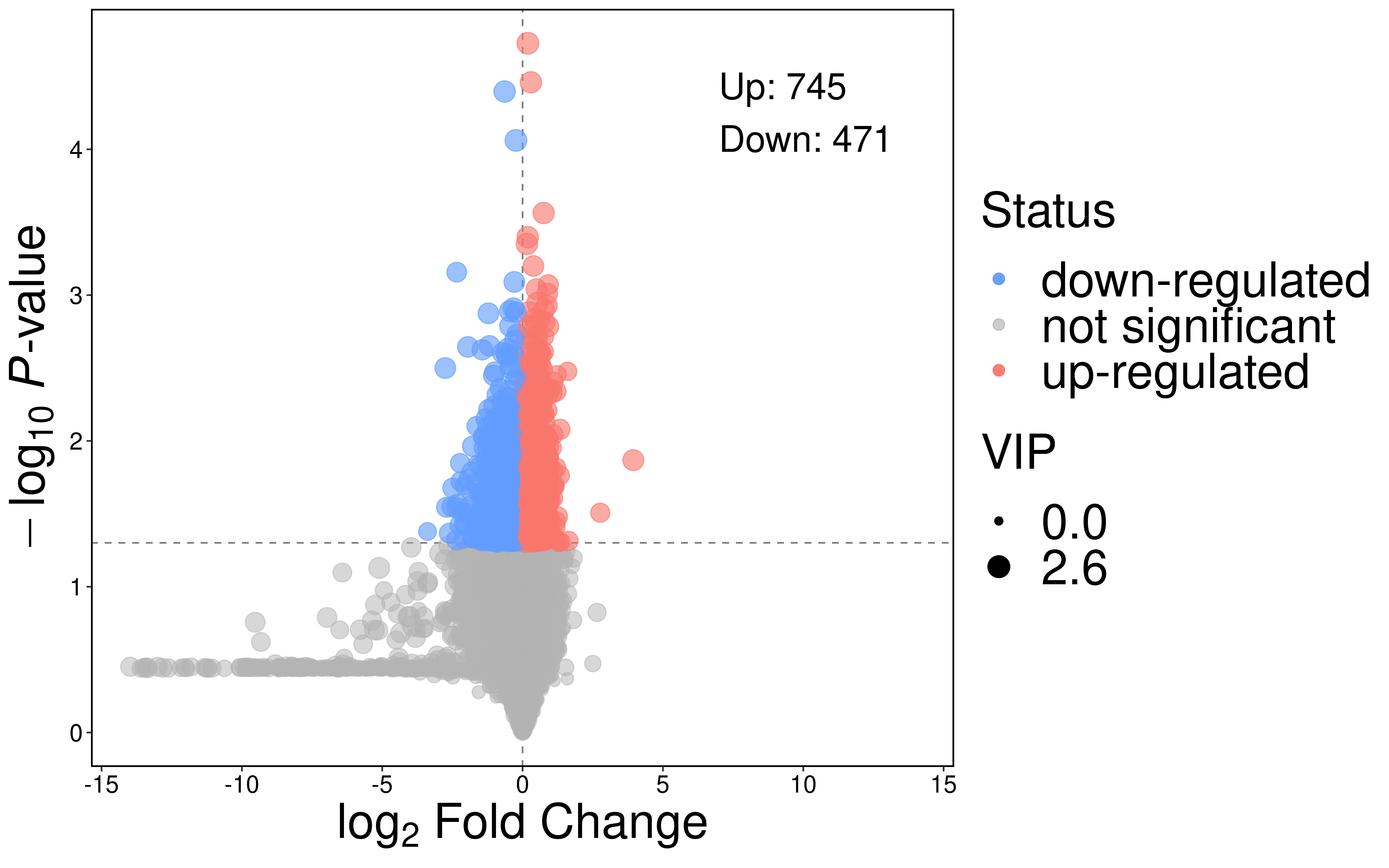

Supplement: S1 File — (ZIP) [file pone.0325562.s001.zip › S1_File/Metabolomic analysis/Statistical Analysis/C-M/volcano plot.jpg]

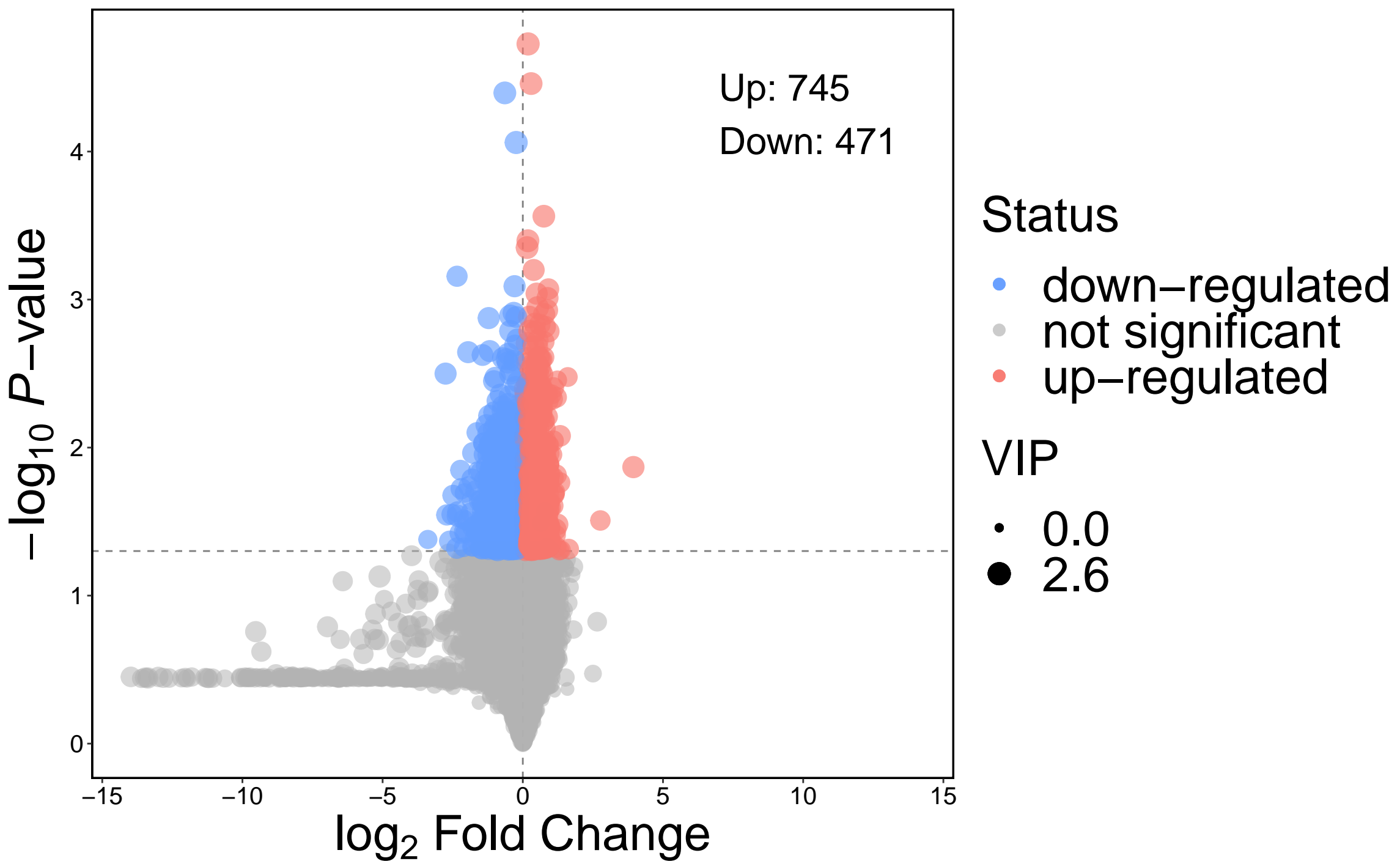

Supplement: S1 File — (ZIP) [file pone.0325562.s001.zip › S1_File/Metabolomic analysis/Statistical Analysis/C-M/volcano plot.pdf]

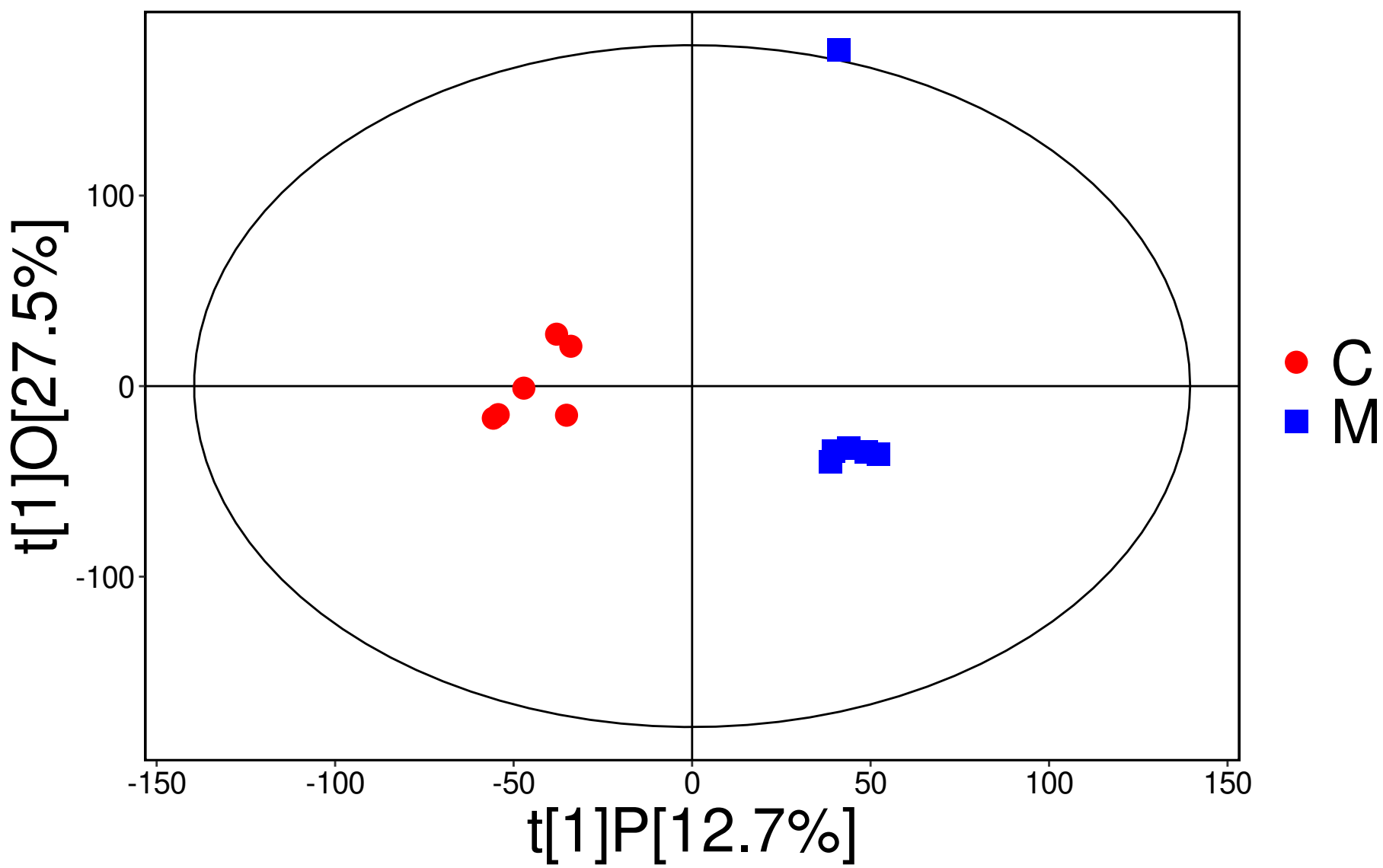

Supplement: S1 File — (ZIP) [file pone.0325562.s001.zip › S1_File/Metabolomic analysis/Statistical Analysis/C-M/OPLS-DA score plot.pdf]

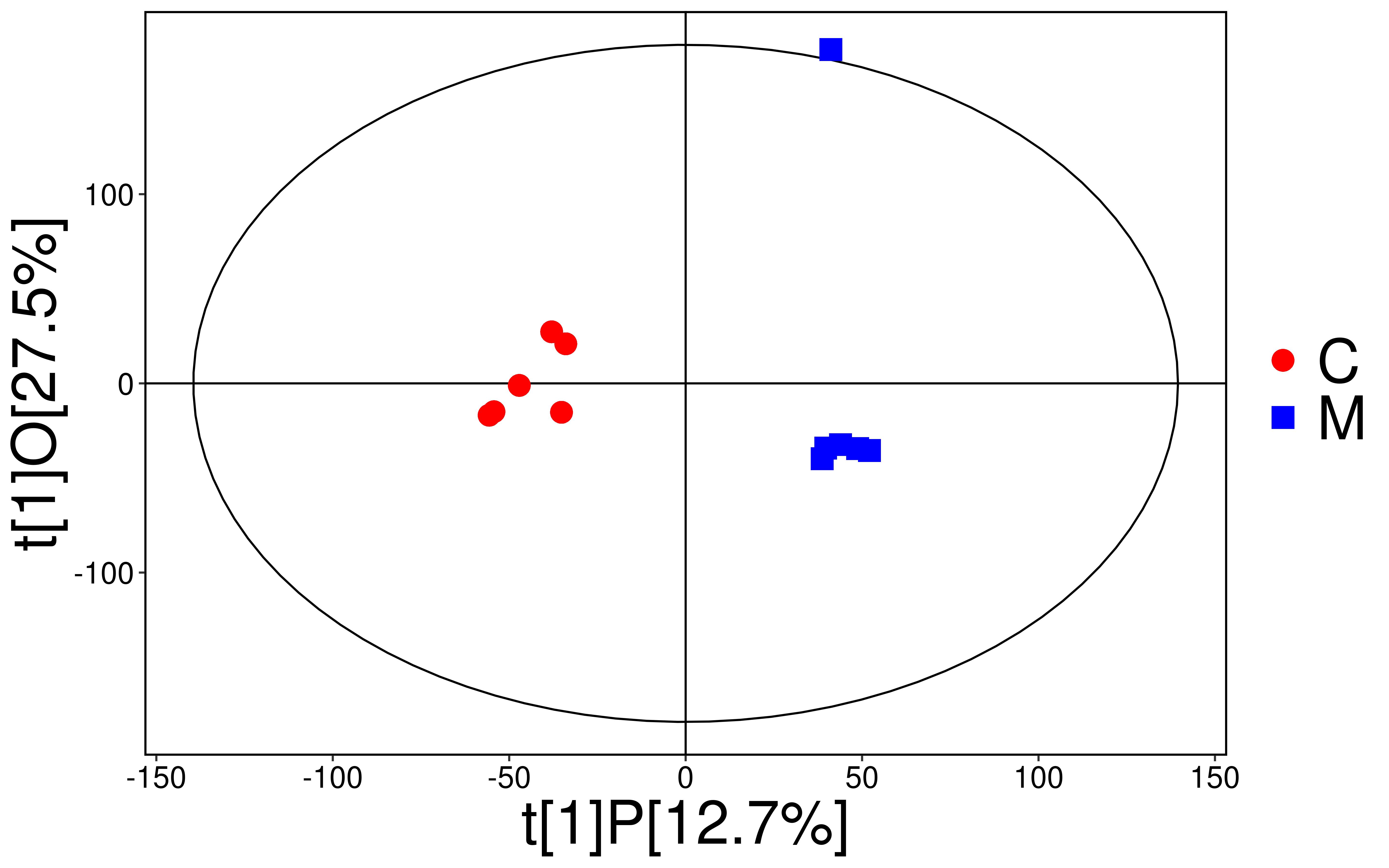

Supplement: S1 File — (ZIP) [file pone.0325562.s001.zip › S1_File/Metabolomic analysis/Statistical Analysis/C-M/OPLS-DA score plot.jpg]

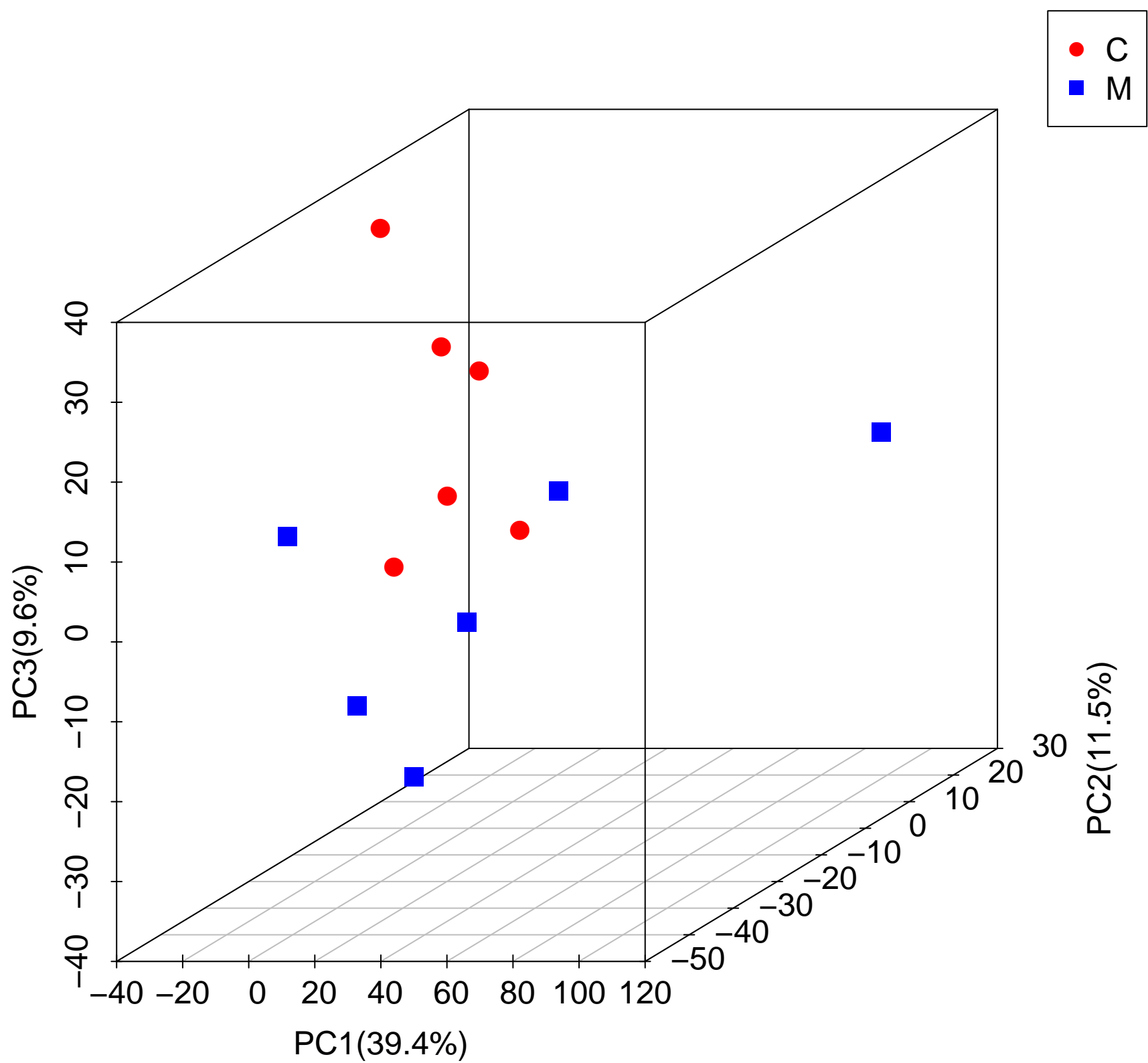

Supplement: S1 File — (ZIP) [file pone.0325562.s001.zip › S1_File/Metabolomic analysis/Statistical Analysis/C-M/PCA score plot 3D.pdf]

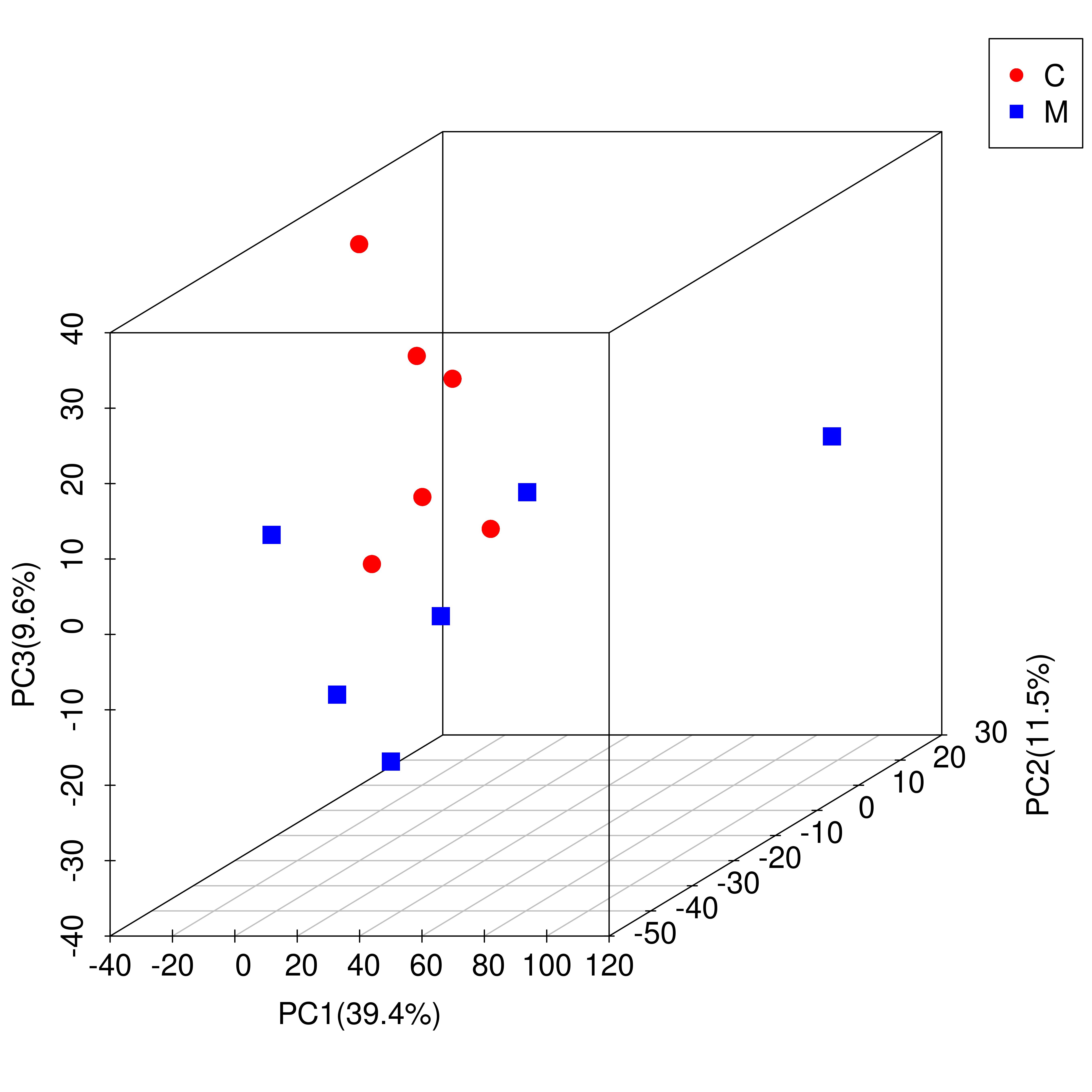

Supplement: S1 File — (ZIP) [file pone.0325562.s001.zip › S1_File/Metabolomic analysis/Statistical Analysis/C-M/PCA score plot 3D.jpg]

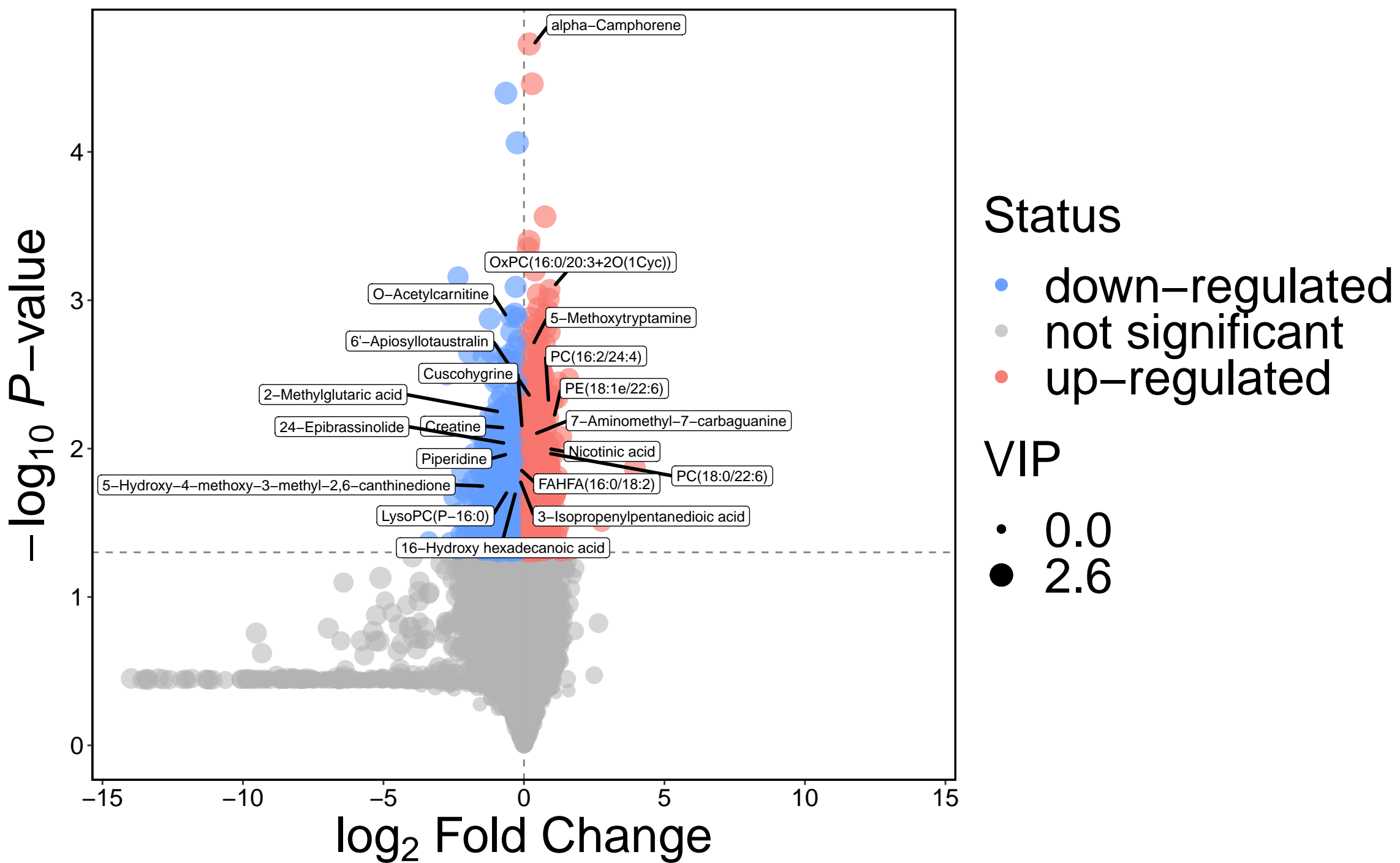

Supplement: S1 File — (ZIP) [file pone.0325562.s001.zip › S1_File/Metabolomic analysis/Statistical Analysis/C-M/volcano plot with label.pdf]

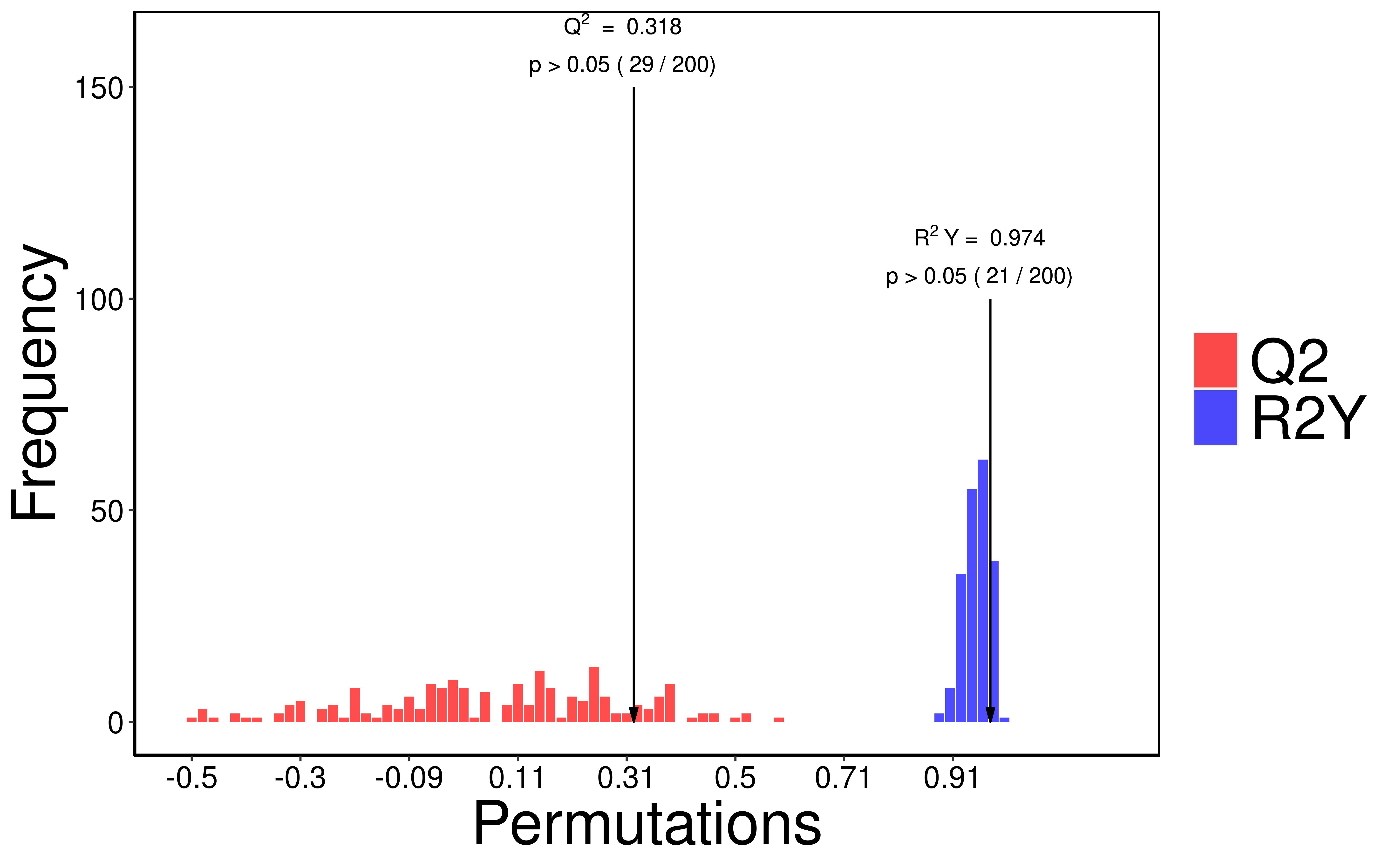

Supplement: S1 File — (ZIP) [file pone.0325562.s001.zip › S1_File/Metabolomic analysis/Statistical Analysis/C-M/OPLS-DA permutation histogram.jpg]

Frequency

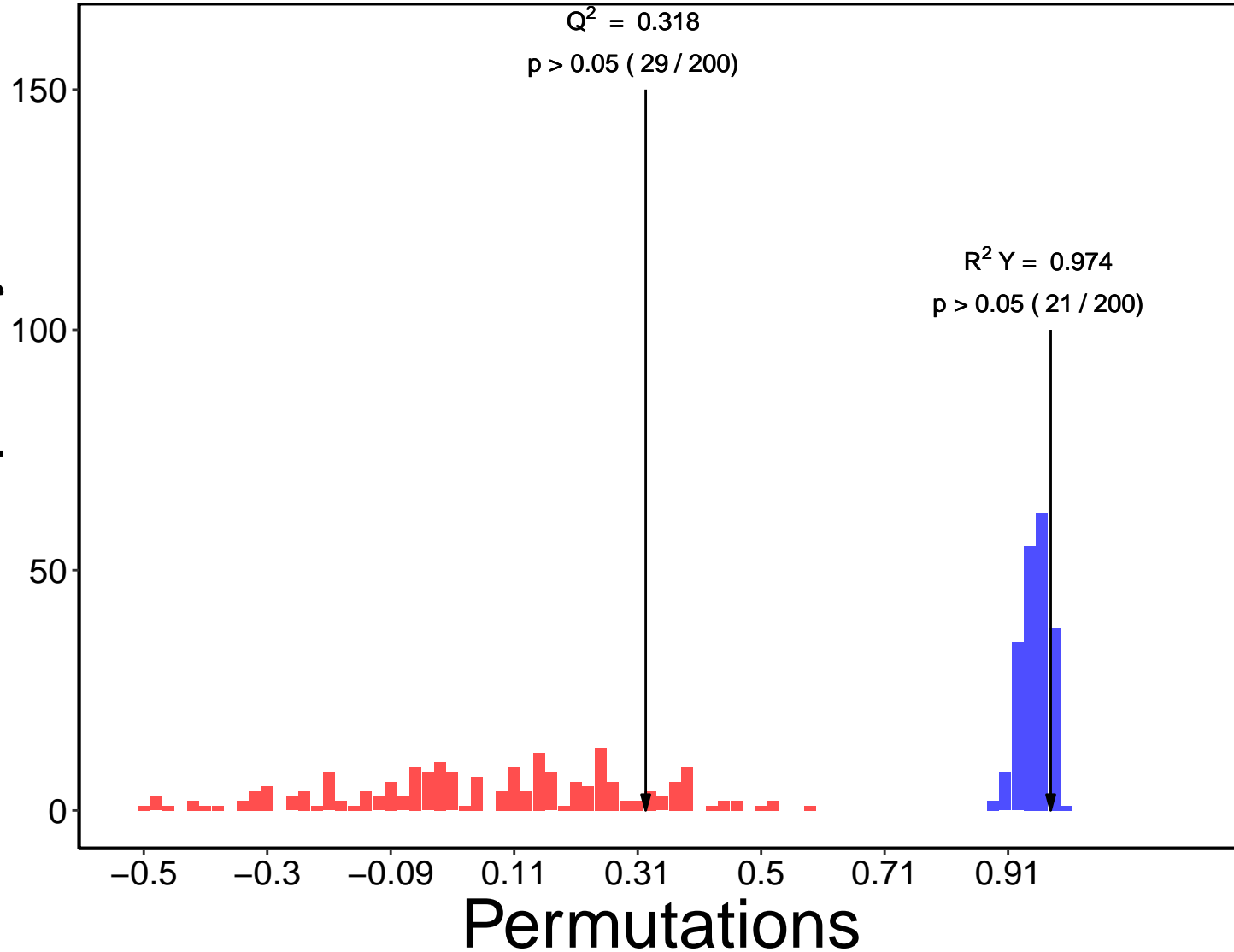

Q2  
R2Y

Supplement: S1 File — (ZIP) [file pone.0325562.s001.zip › S1_File/Metabolomic analysis/Statistical Analysis/C-M/OPLS-DA permutation histogram.pdf]

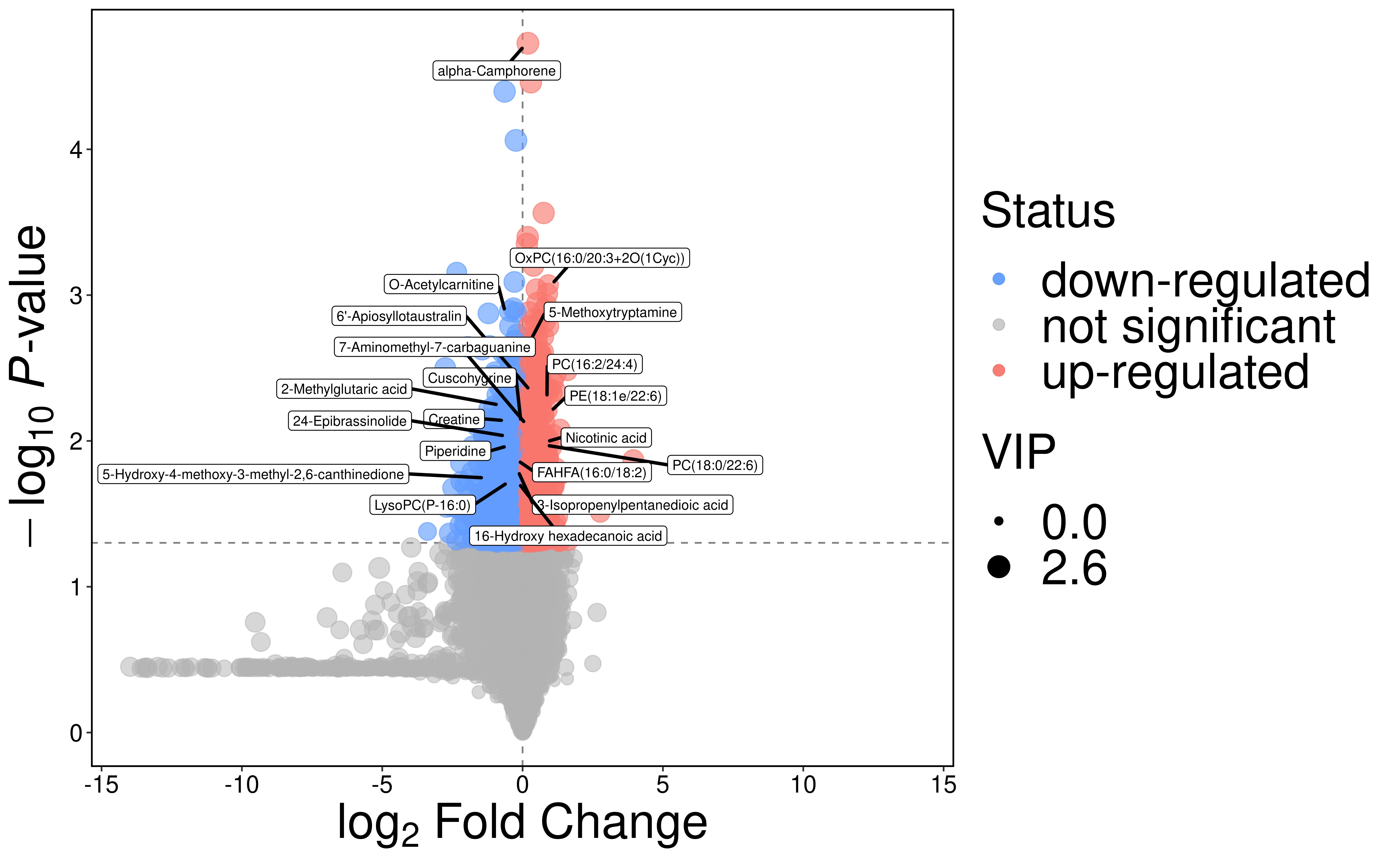

Supplement: S1 File — (ZIP) [file pone.0325562.s001.zip › S1_File/Metabolomic analysis/Statistical Analysis/C-M/volcano plot with label.jpg]

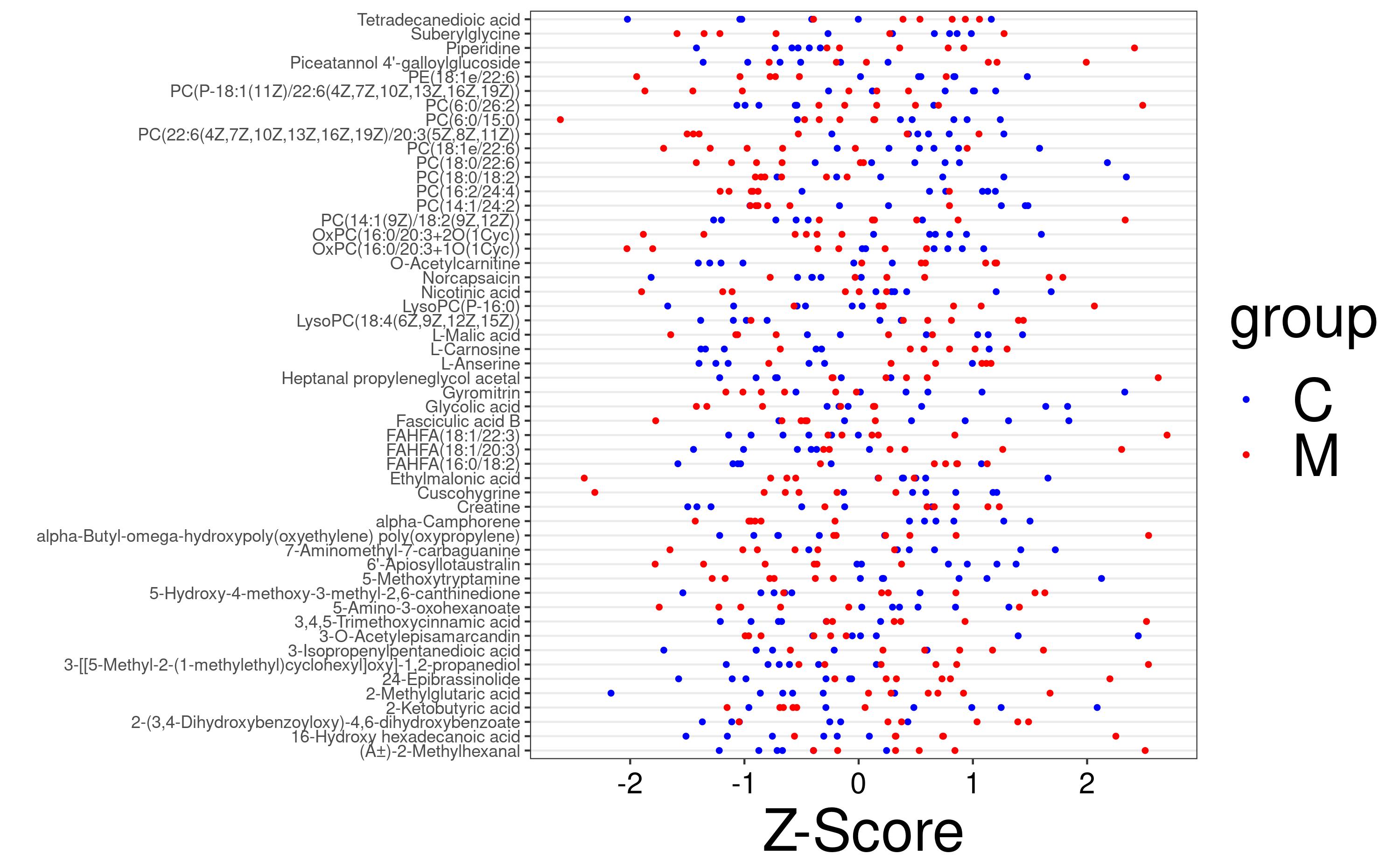

Supplement: S1 File — (ZIP) [file pone.0325562.s001.zip › S1_File/Metabolomic analysis/Statistical Analysis/C-M/Z-score Plot.jpg]

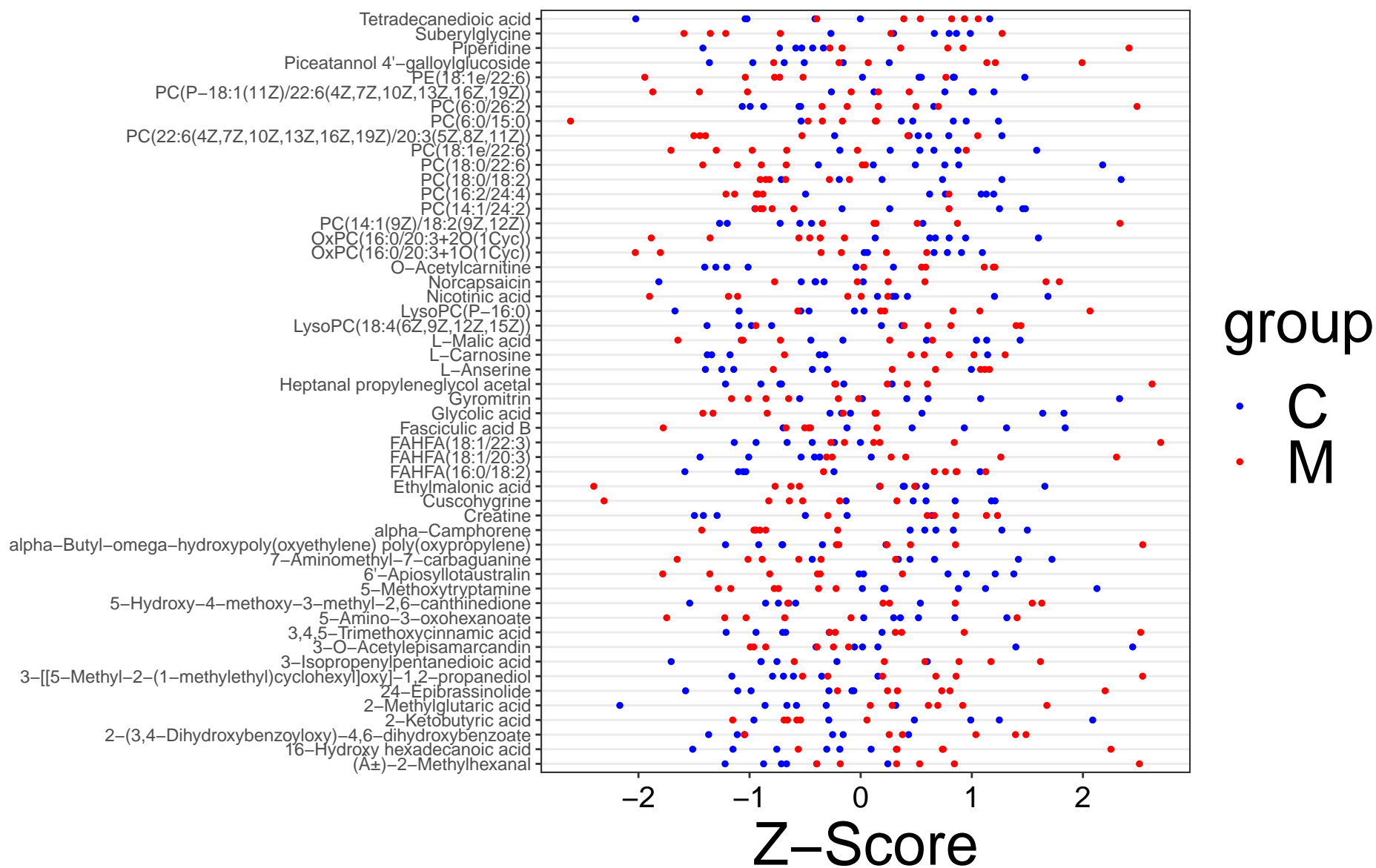

Supplement: S1 File — (ZIP) [file pone.0325562.s001.zip › S1_File/Metabolomic analysis/Statistical Analysis/C-M/Z-Score Plot.pdf]

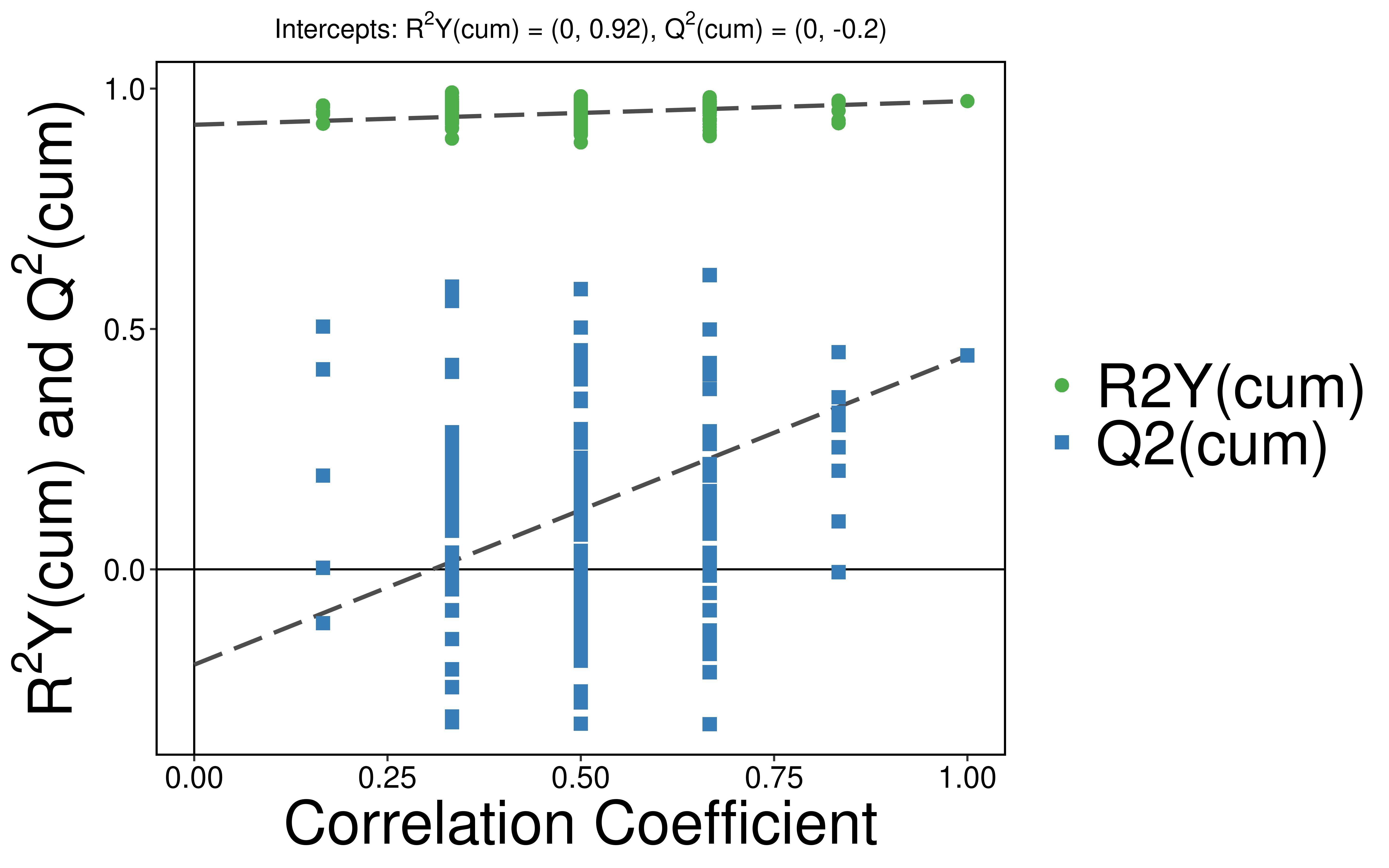

Supplement: S1 File — (ZIP) [file pone.0325562.s001.zip › S1_File/Metabolomic analysis/Statistical Analysis/M-C/OPLS-DA permutation plot.jpg]

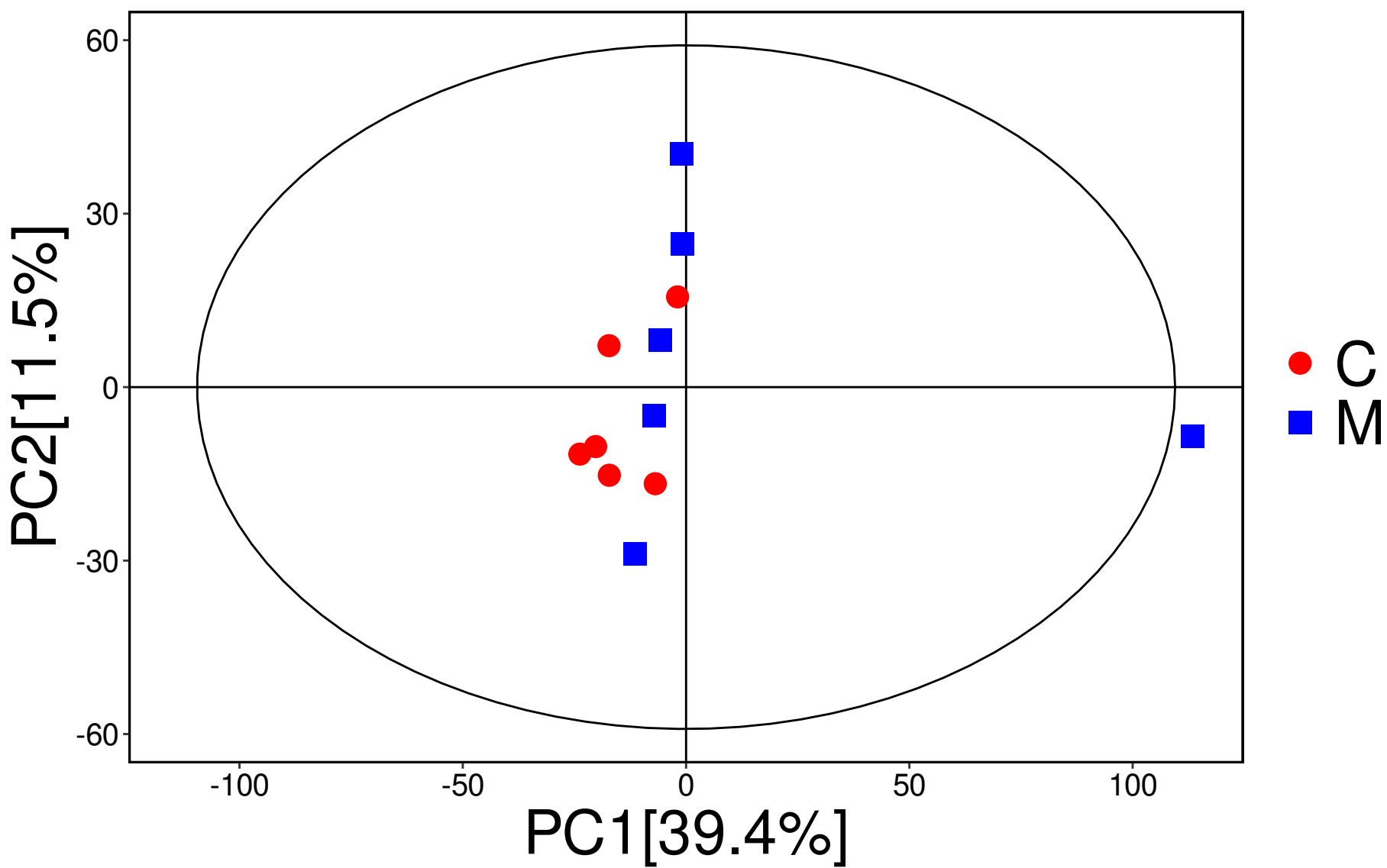

Supplement: S1 File — (ZIP) [file pone.0325562.s001.zip › S1_File/Metabolomic analysis/Statistical Analysis/M-C/PCA score plot.pdf]

Intercepts:  $R^2Y(\text{cum}) = (0, 0.92)$ ,  $Q^2(\text{cum}) = (0, -0.2)$

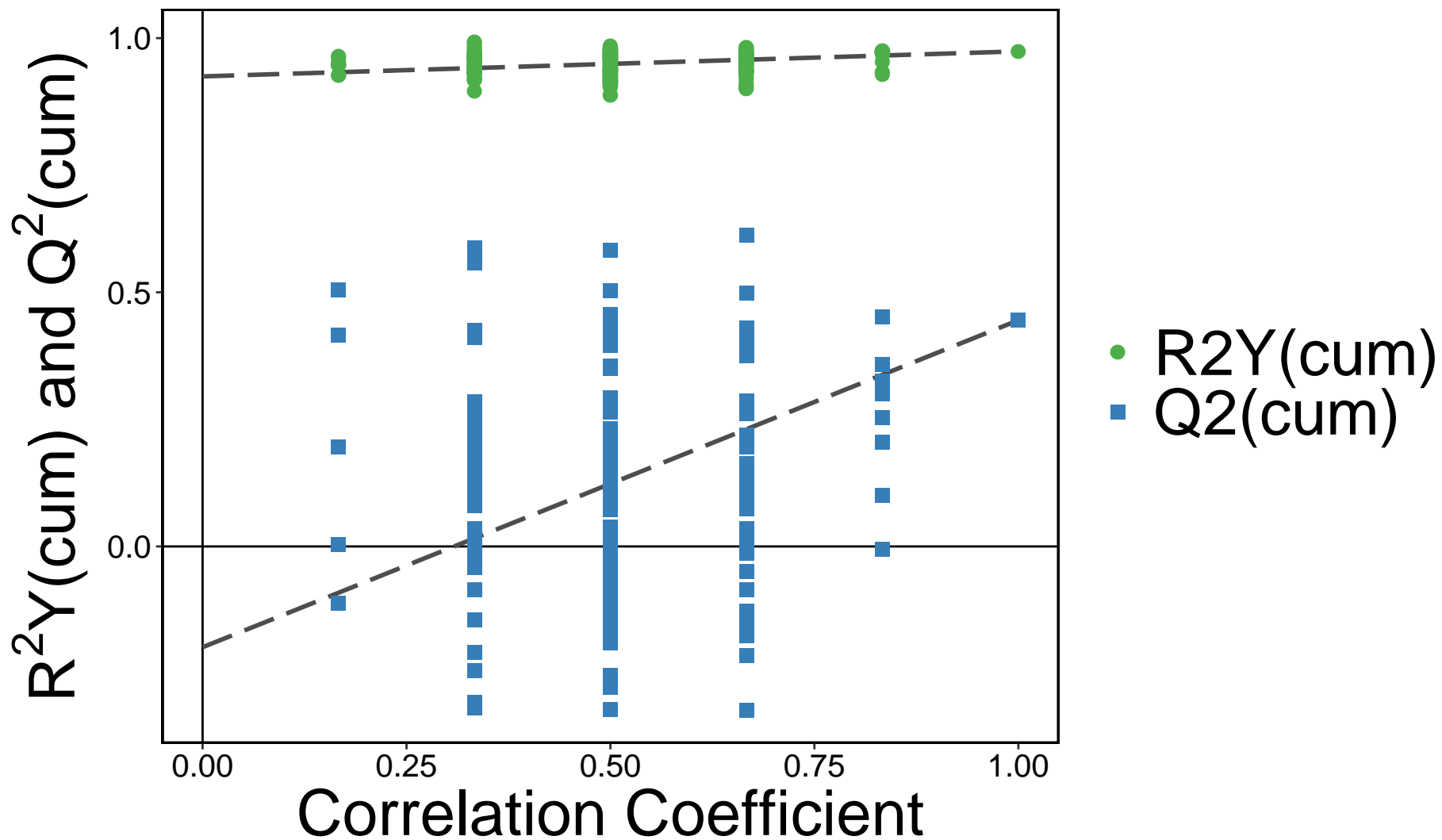

Supplement: S1 File — (ZIP) [file pone.0325562.s001.zip › S1_File/Metabolomic analysis/Statistical Analysis/M-C/OPLS-DA permutation plot.pdf]

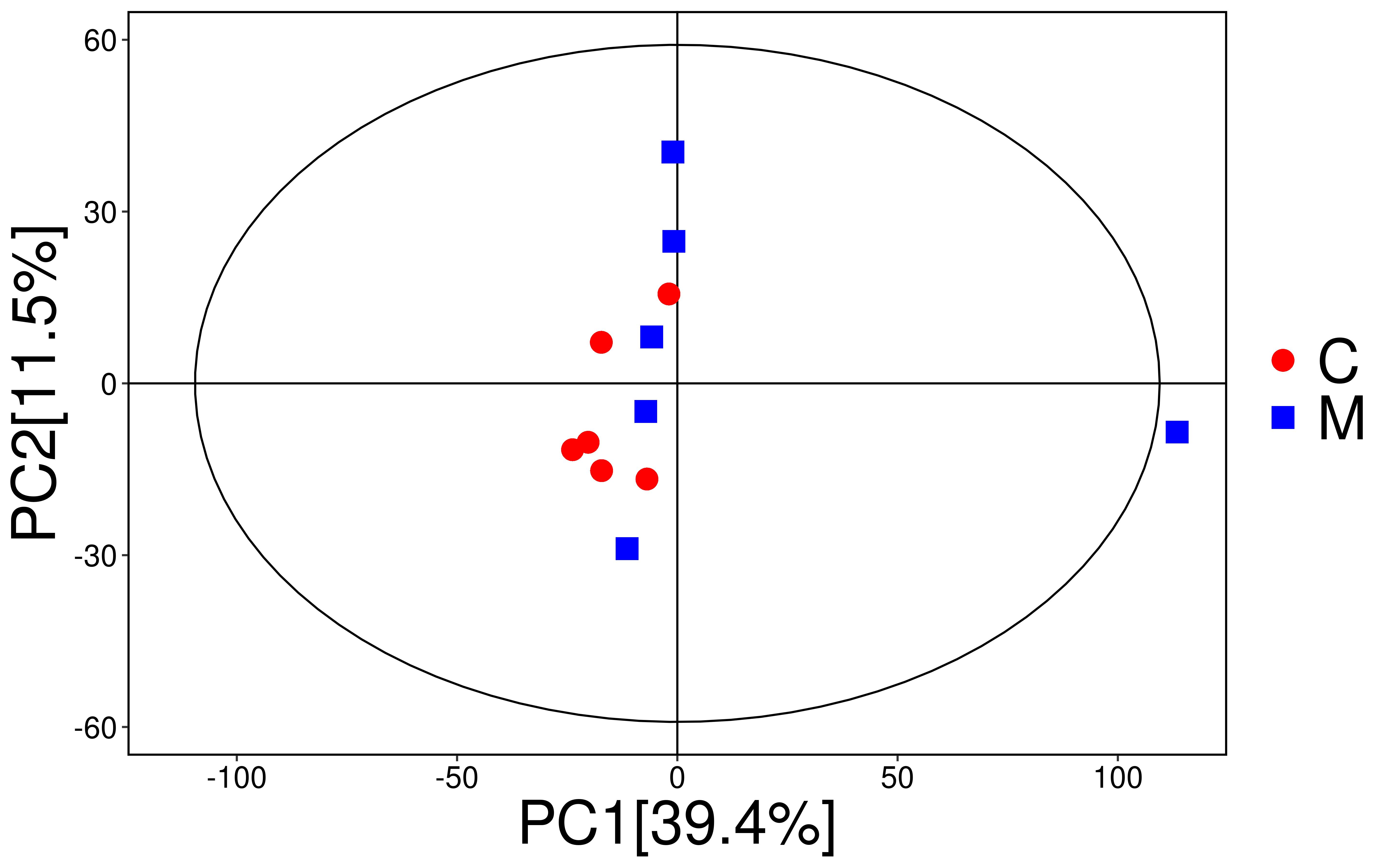

Supplement: S1 File — (ZIP) [file pone.0325562.s001.zip › S1_File/Metabolomic analysis/Statistical Analysis/M-C/PCA score plot.jpg]

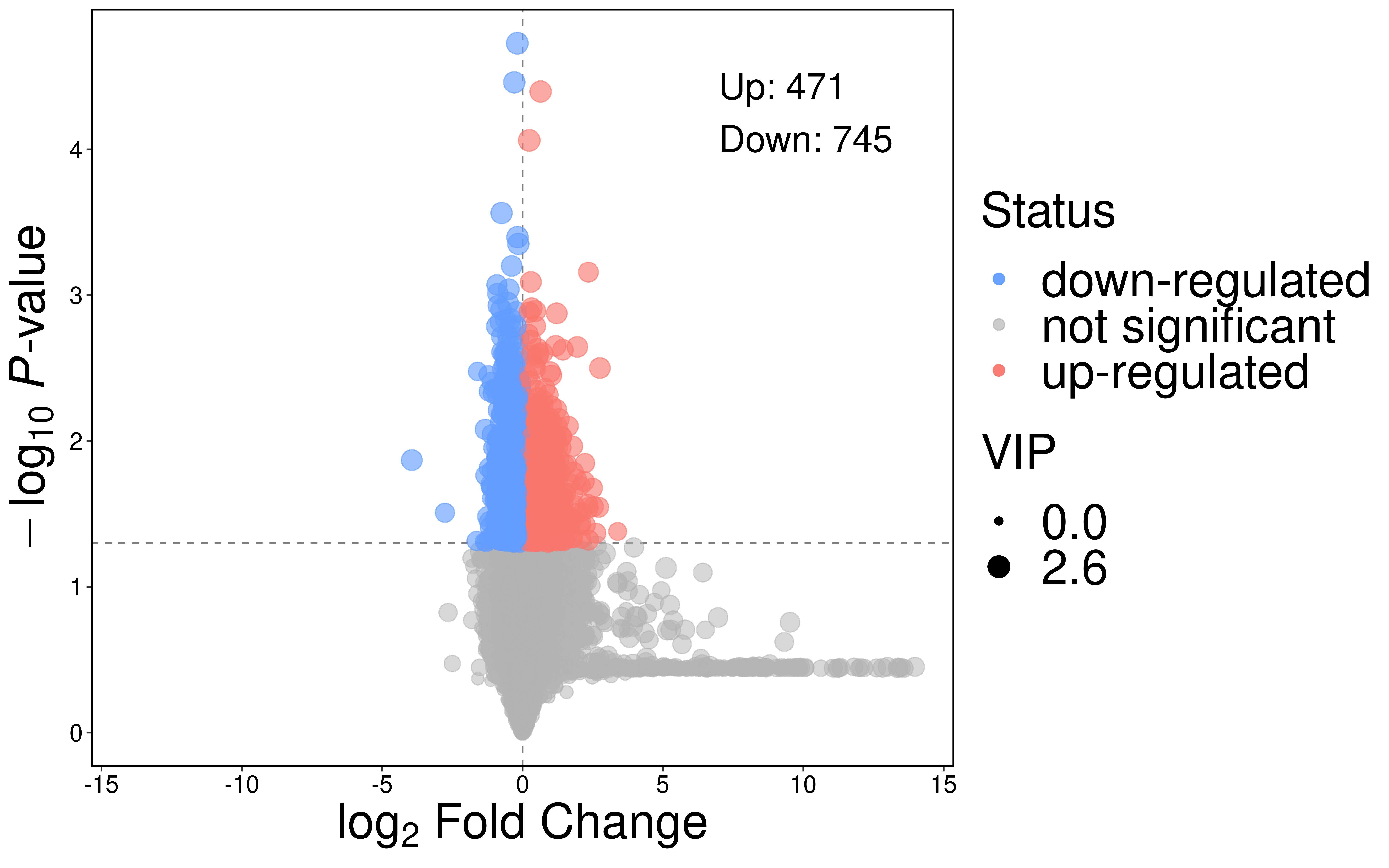

Supplement: S1 File — (ZIP) [file pone.0325562.s001.zip › S1_File/Metabolomic analysis/Statistical Analysis/M-C/volcano plot.jpg]

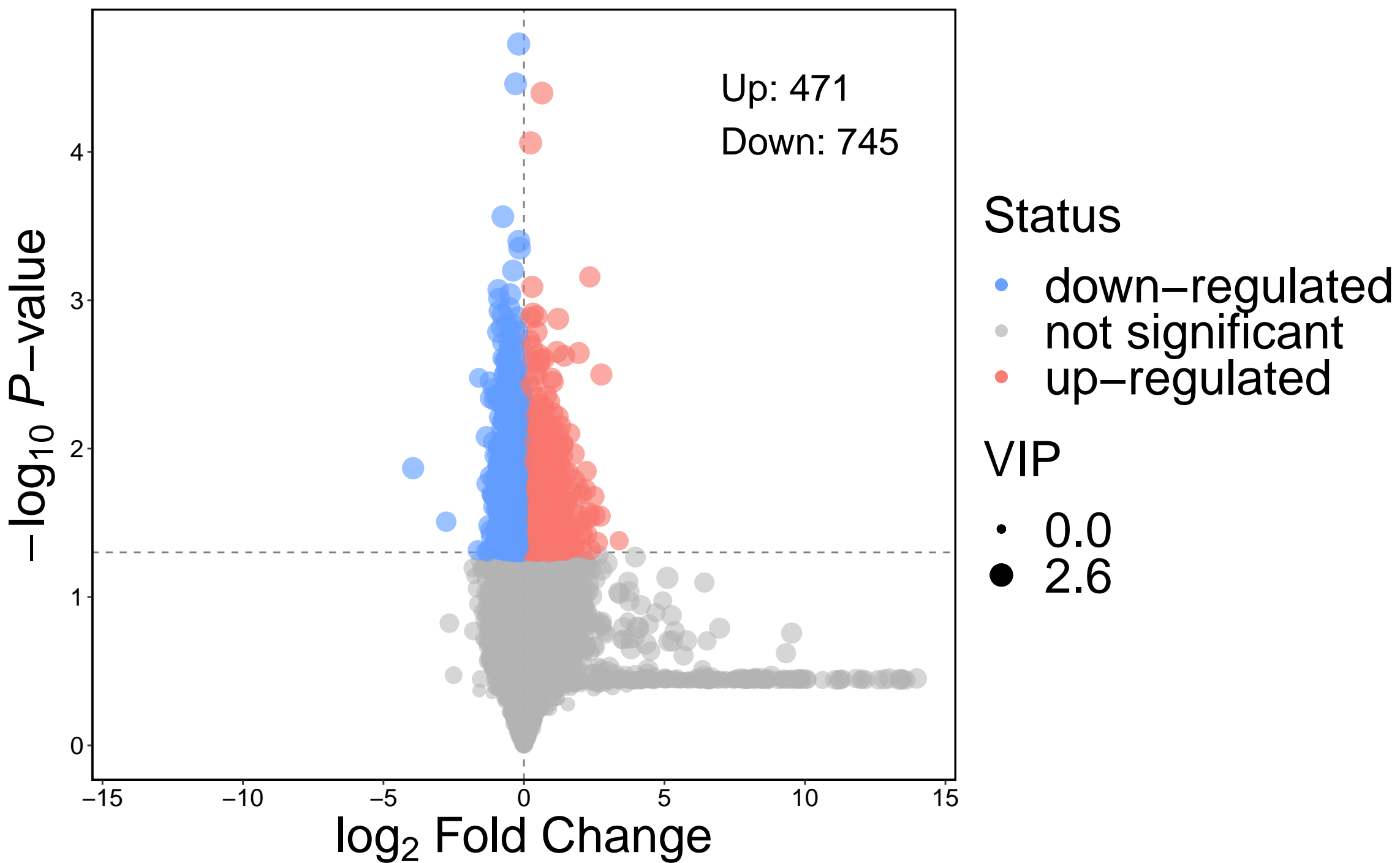

Supplement: S1 File — (ZIP) [file pone.0325562.s001.zip › S1_File/Metabolomic analysis/Statistical Analysis/M-C/volcano plot.pdf]

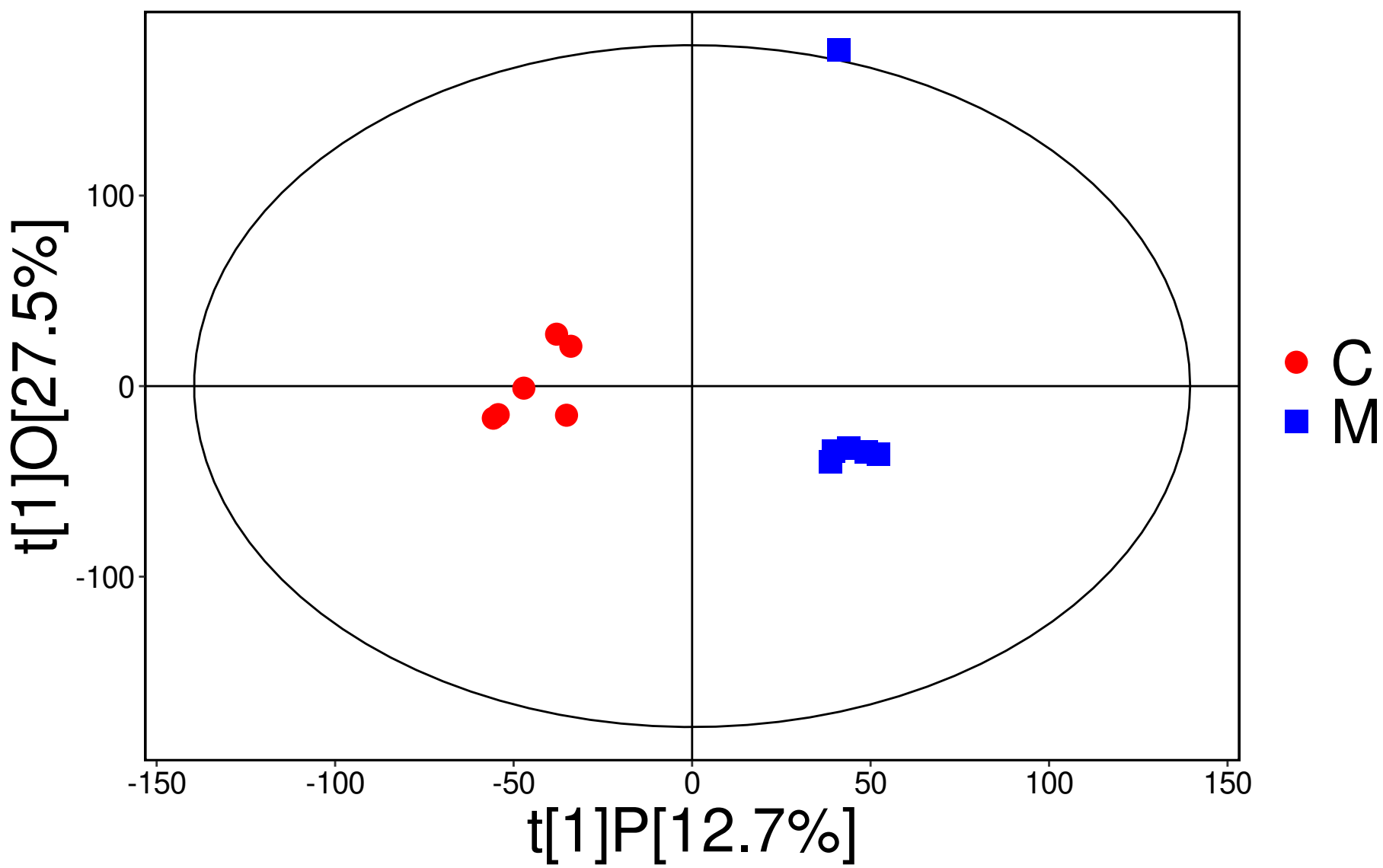

Supplement: S1 File — (ZIP) [file pone.0325562.s001.zip › S1_File/Metabolomic analysis/Statistical Analysis/M-C/OPLS-DA score plot.pdf]

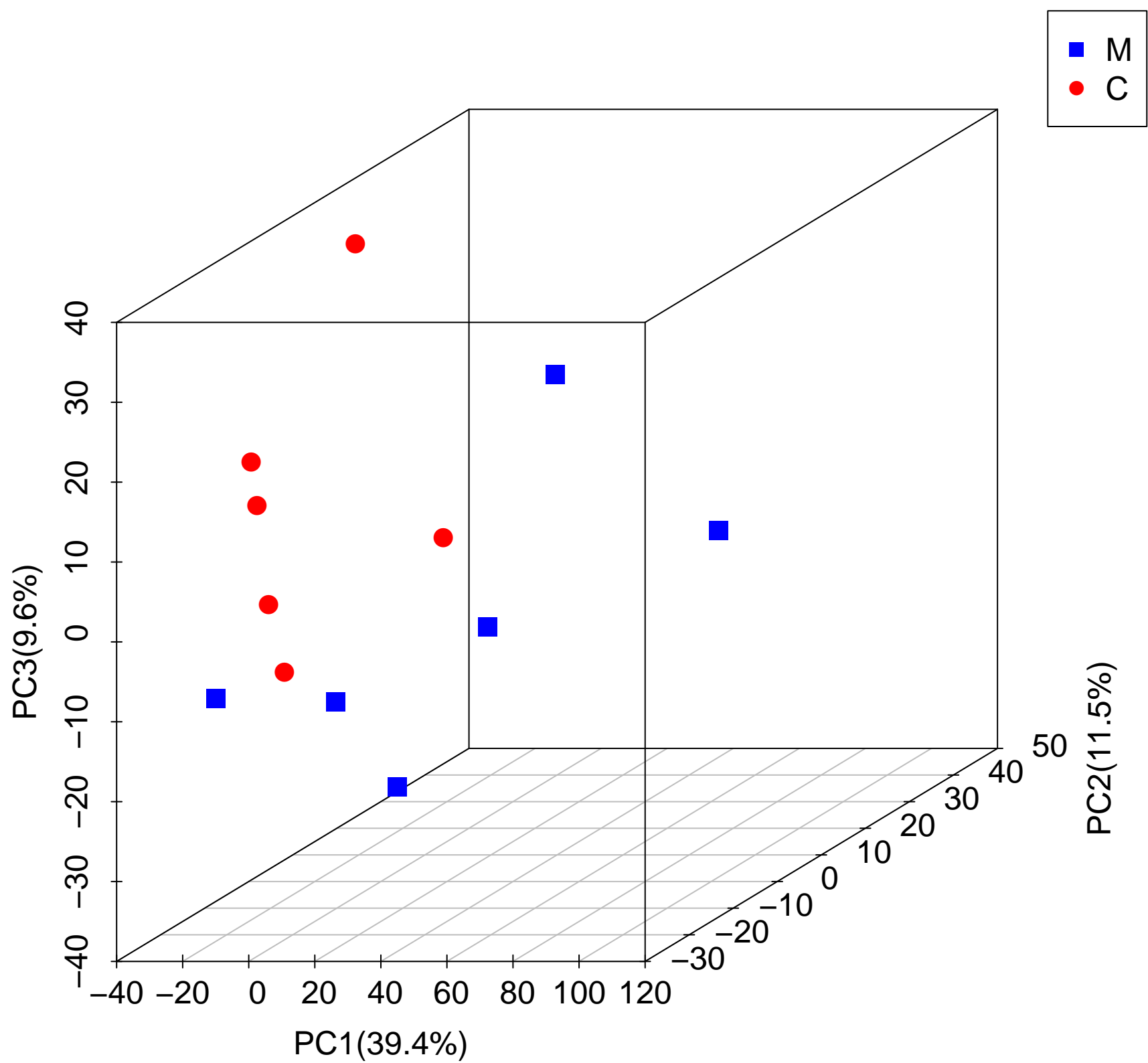

Supplement: S1 File — (ZIP) [file pone.0325562.s001.zip › S1_File/Metabolomic analysis/Statistical Analysis/M-C/PCA score plot 3D.pdf]

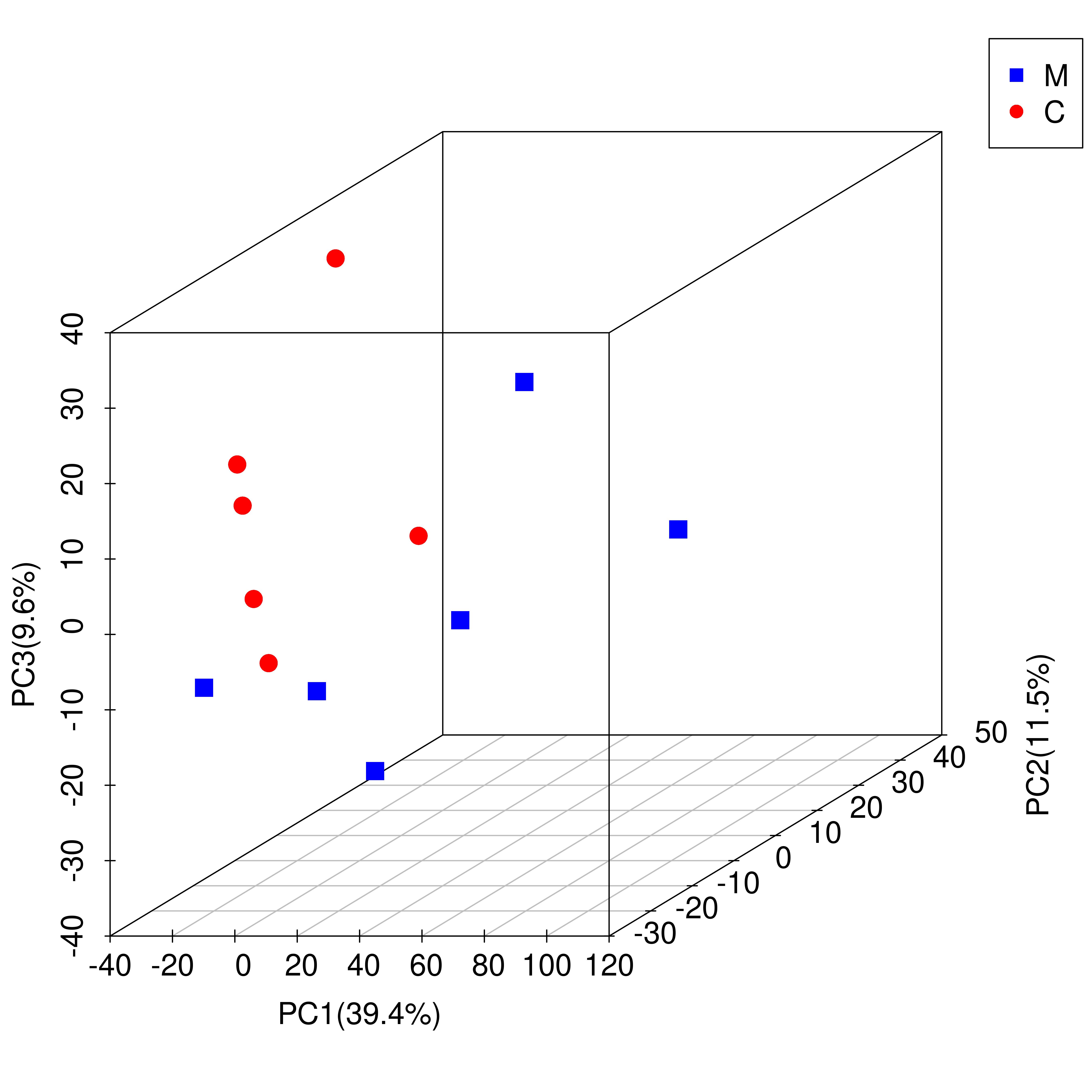

Supplement: S1 File — (ZIP) [file pone.0325562.s001.zip › S1_File/Metabolomic analysis/Statistical Analysis/M-C/PCA score plot 3D.jpg]

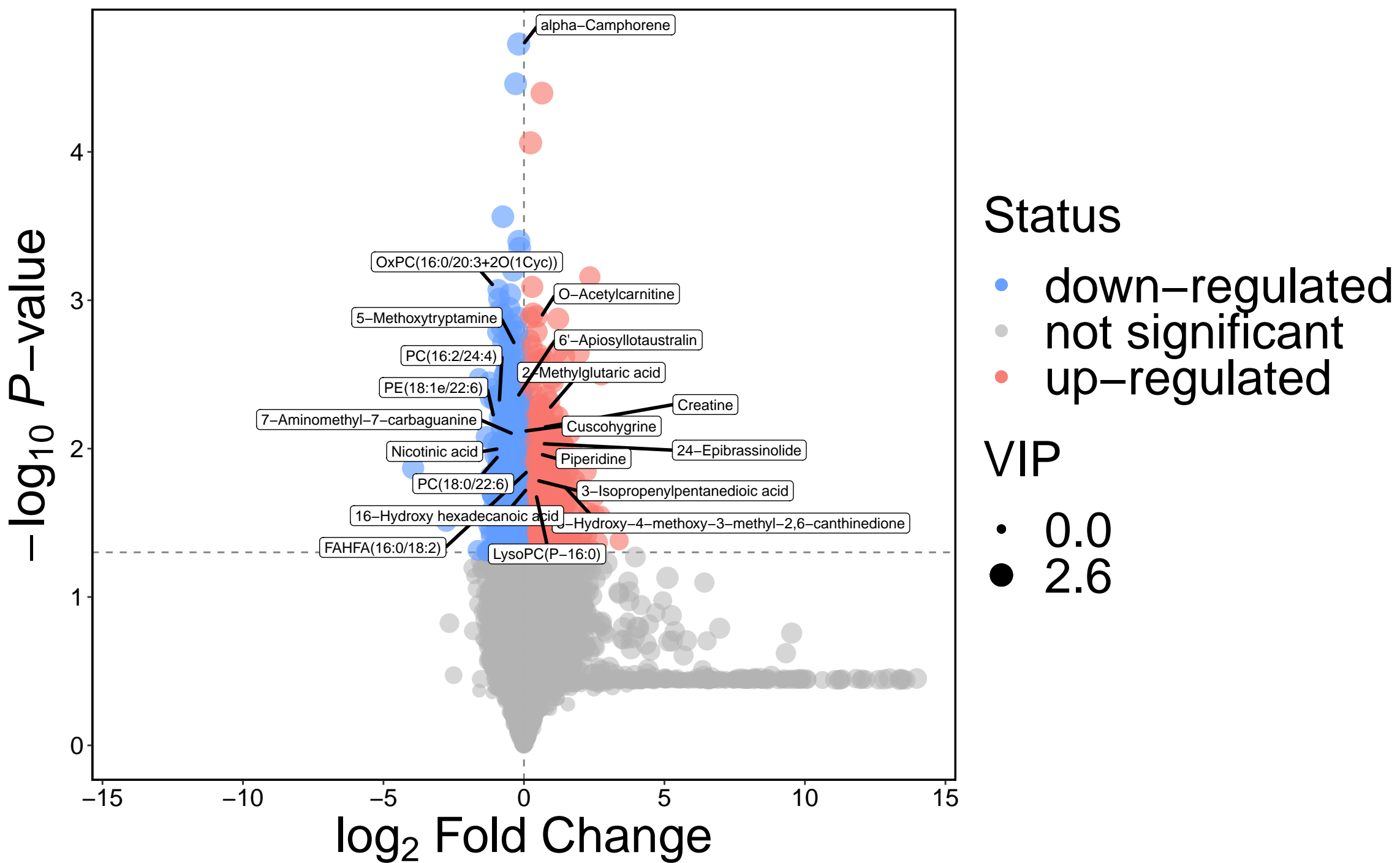

Supplement: S1 File — (ZIP) [file pone.0325562.s001.zip › S1_File/Metabolomic analysis/Statistical Analysis/M-C/volcano plot with label.pdf]

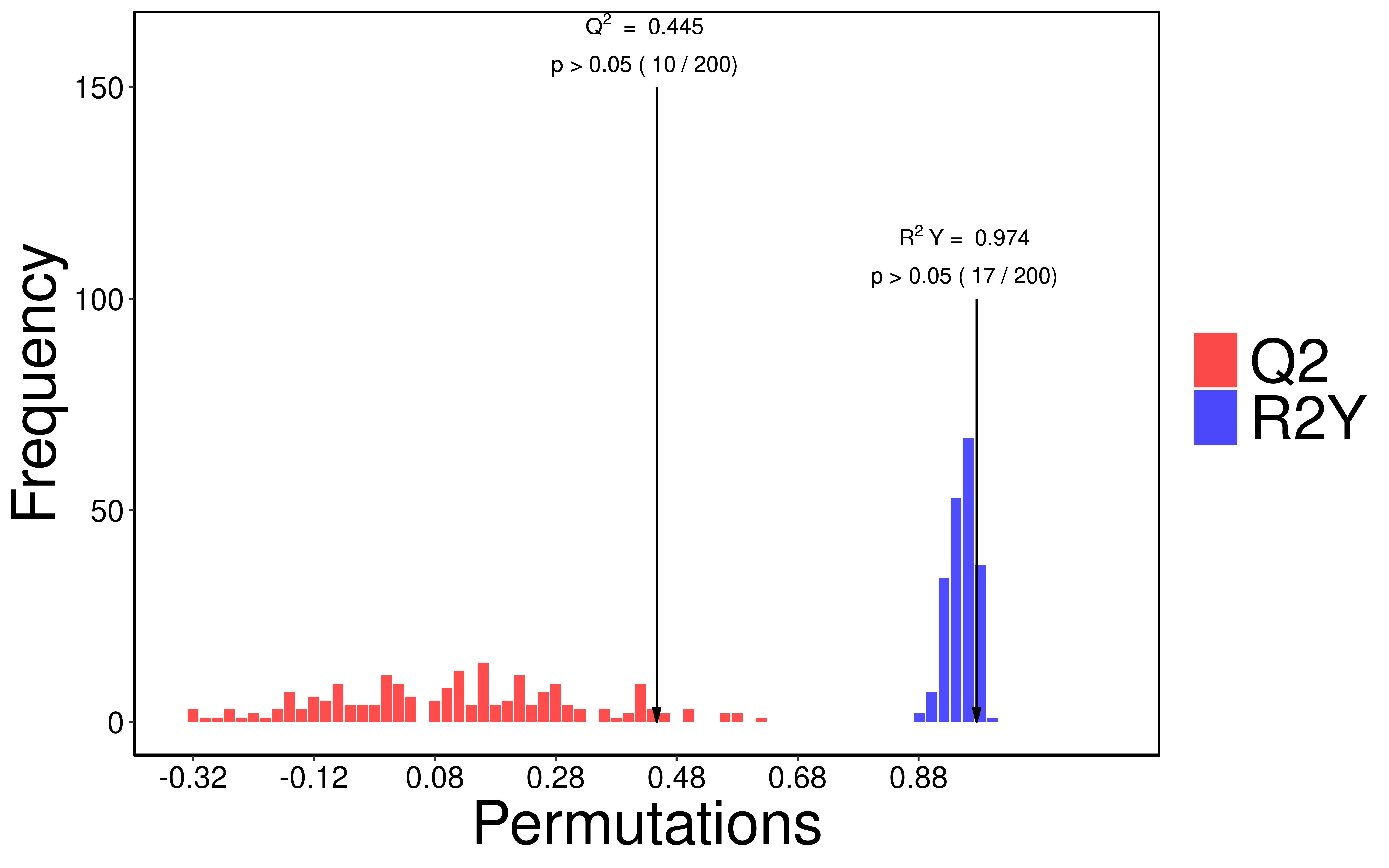

Supplement: S1 File — (ZIP) [file pone.0325562.s001.zip › S1_File/Metabolomic analysis/Statistical Analysis/M-C/OPLS-DA permutation histogram.jpg]

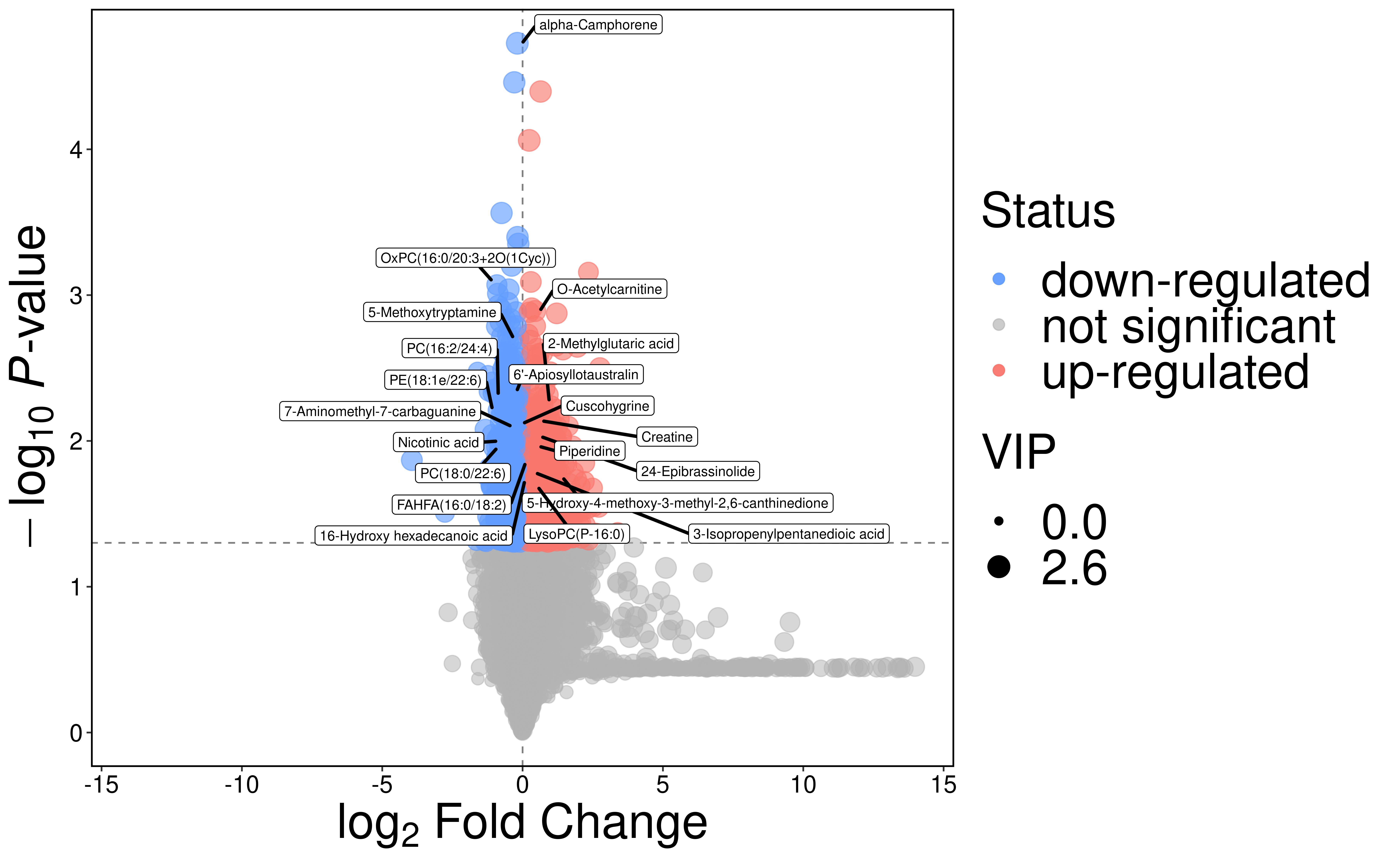

Supplement: S1 File — (ZIP) [file pone.0325562.s001.zip › S1_File/Metabolomic analysis/Statistical Analysis/M-C/volcano plot with label.jpg]

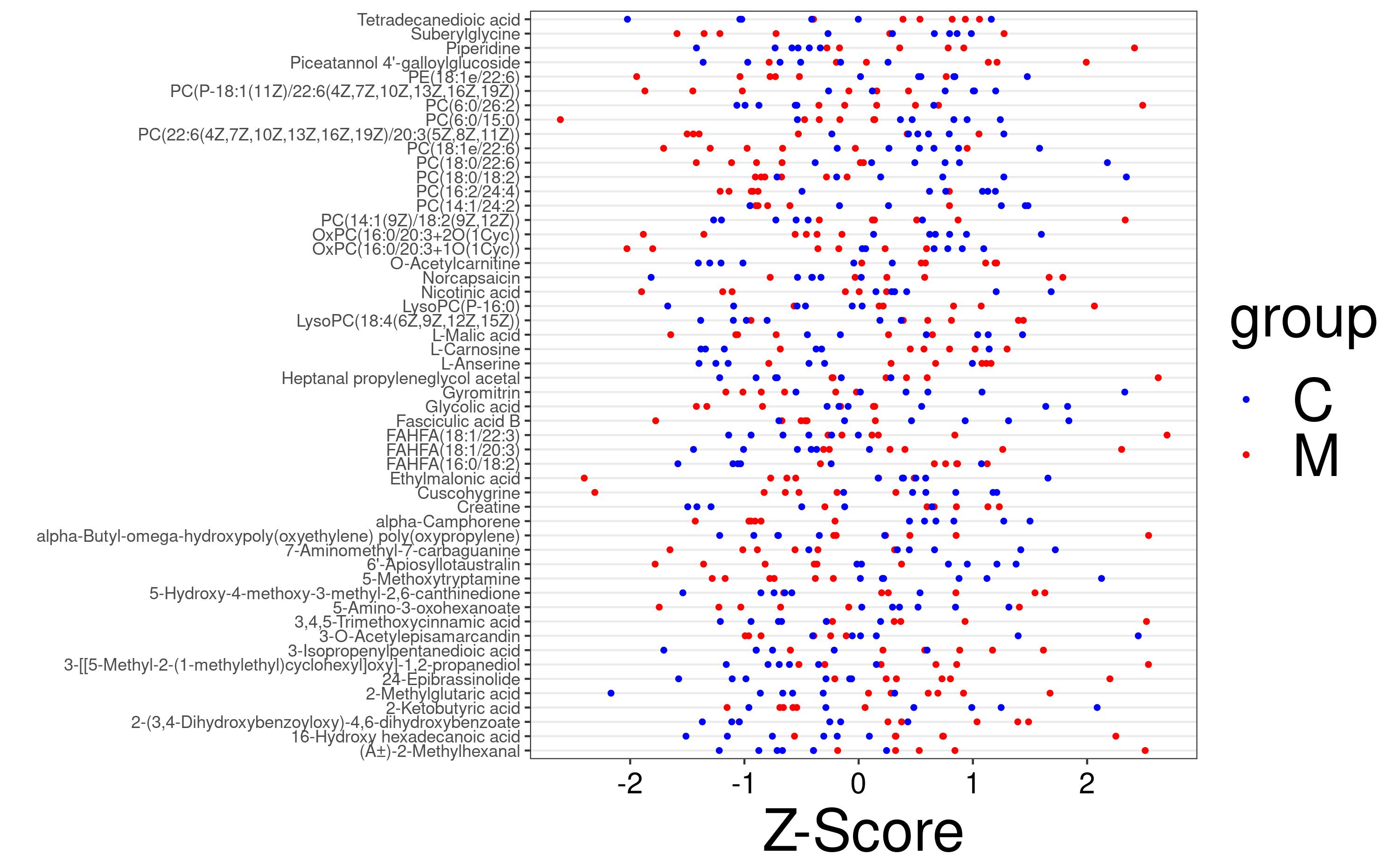

Supplement: S1 File — (ZIP) [file pone.0325562.s001.zip › S1_File/Metabolomic analysis/Statistical Analysis/M-C/Z-score Plot.jpg]

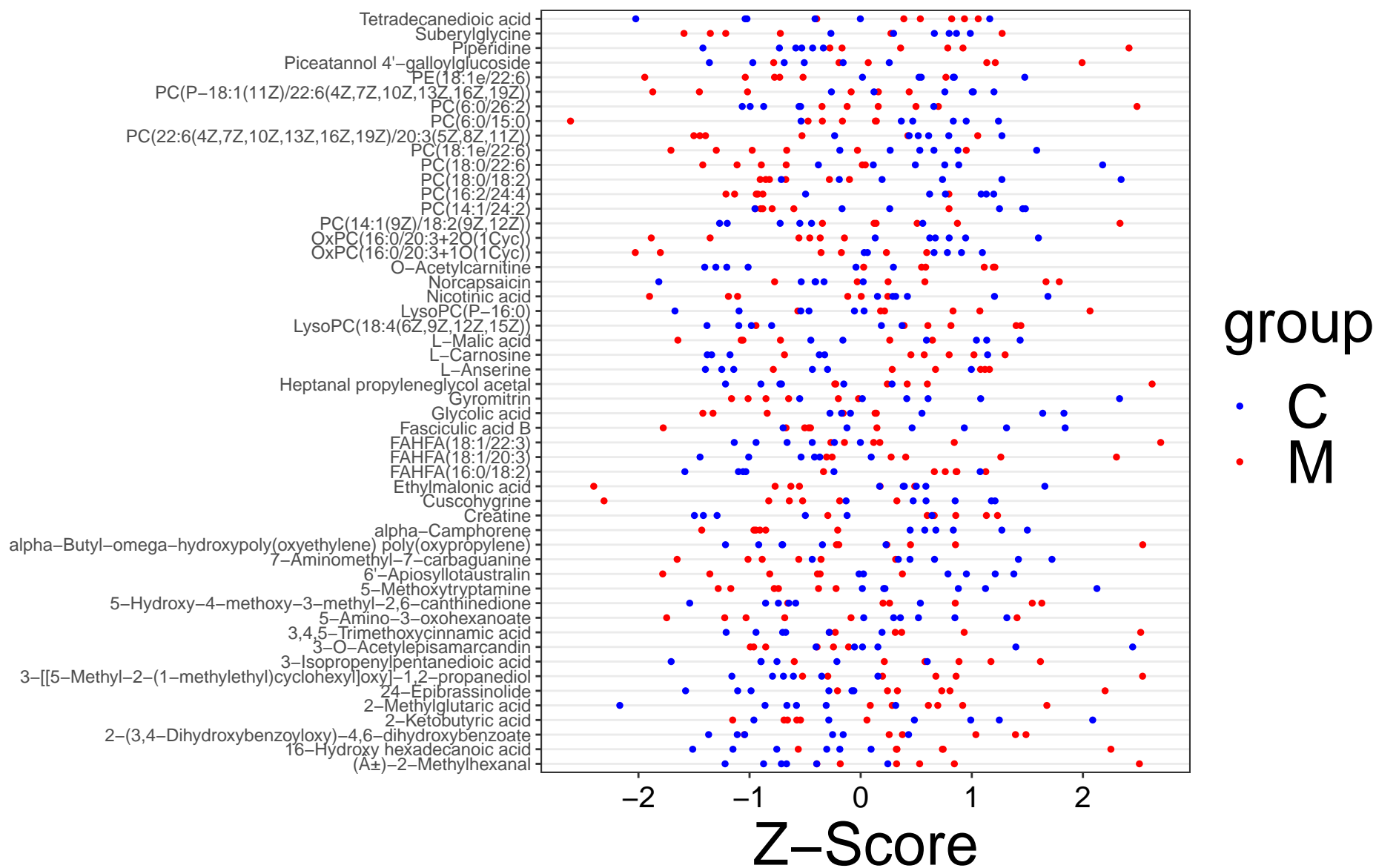

Supplement: S1 File — (ZIP) [file pone.0325562.s001.zip › S1_File/Metabolomic analysis/Statistical Analysis/M-C/Z-Score Plot.pdf]

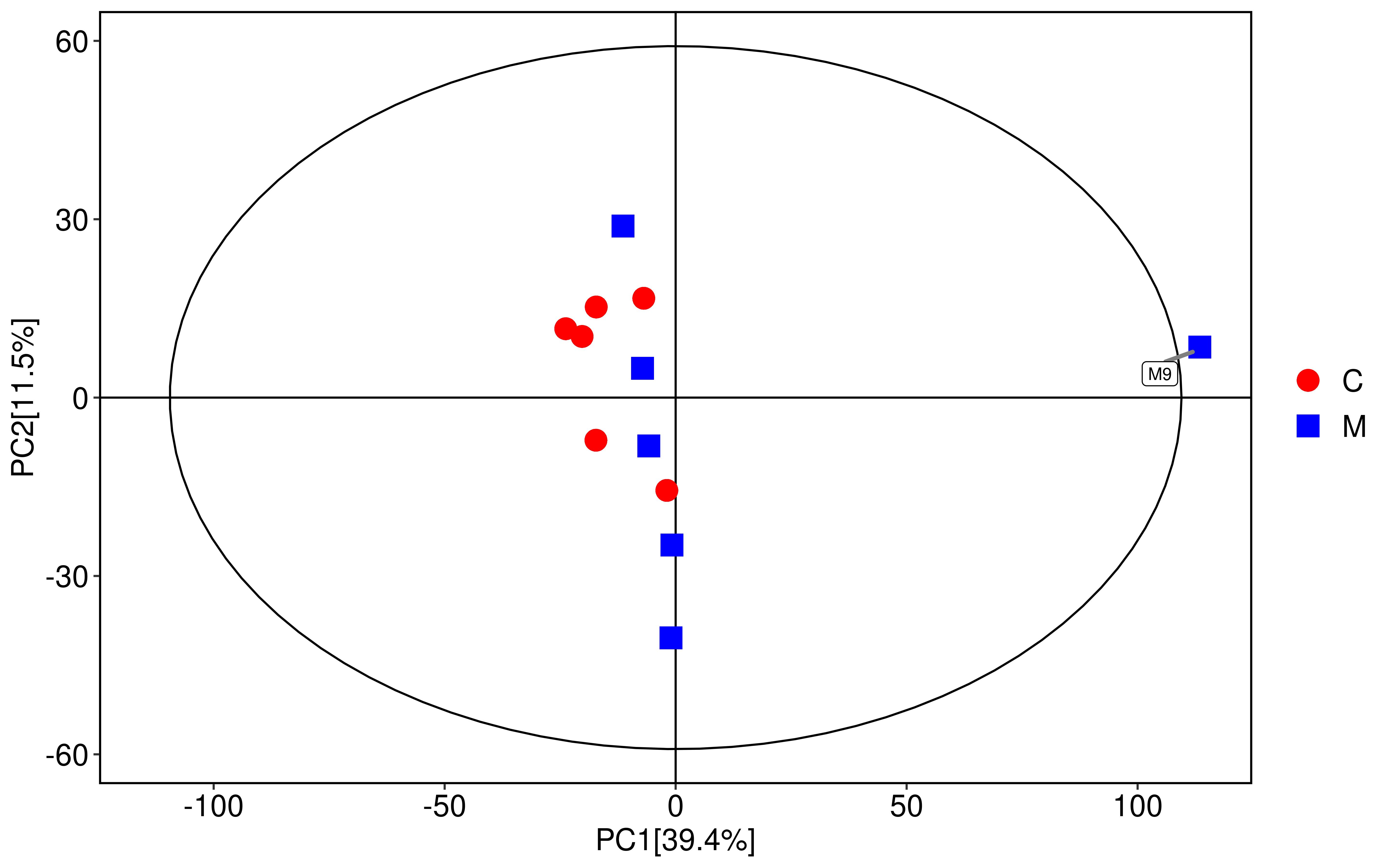

Supplement: S1 File — (ZIP) [file pone.0325562.s001.zip › S1_File/Metabolomic analysis/Statistical Analysis/TOTAL/PCA score label plot.jpg]

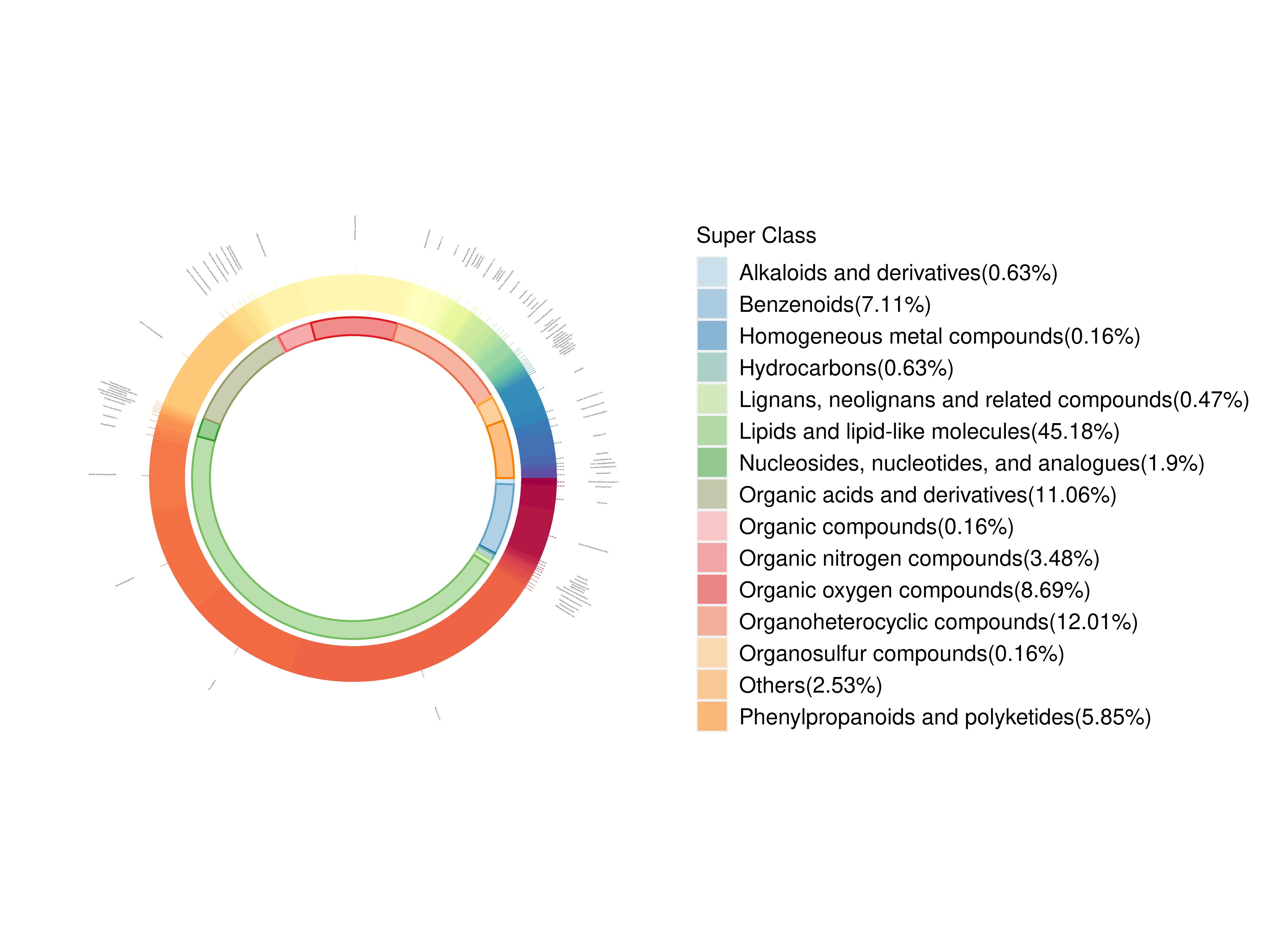

Supplement: S1 File — (ZIP) [file pone.0325562.s001.zip › S1_File/Metabolomic analysis/Statistical Analysis/TOTAL/DonutPlot.jpg]

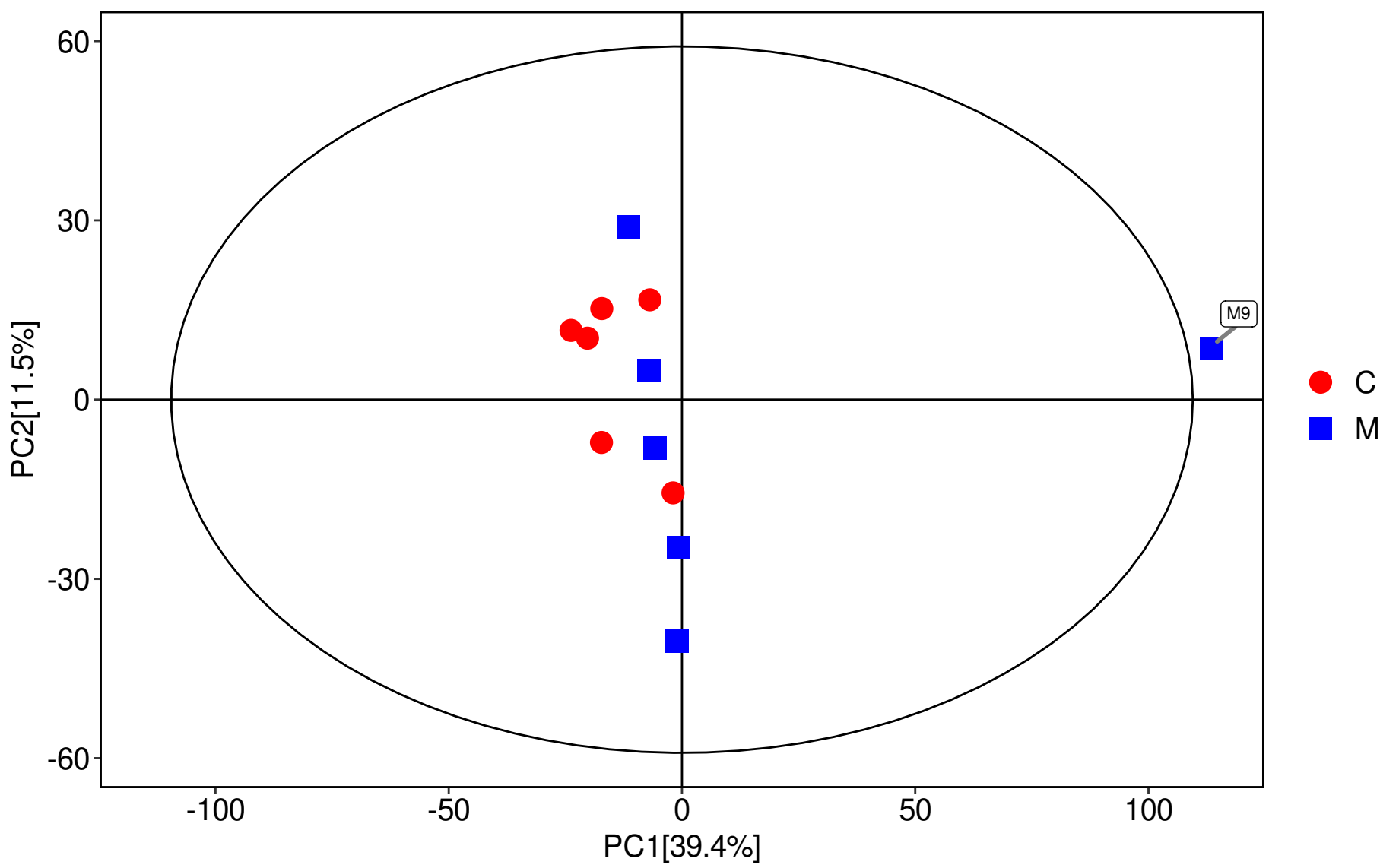

Supplement: S1 File — (ZIP) [file pone.0325562.s001.zip › S1_File/Metabolomic analysis/Statistical Analysis/TOTAL/PCA score label plot.pdf]

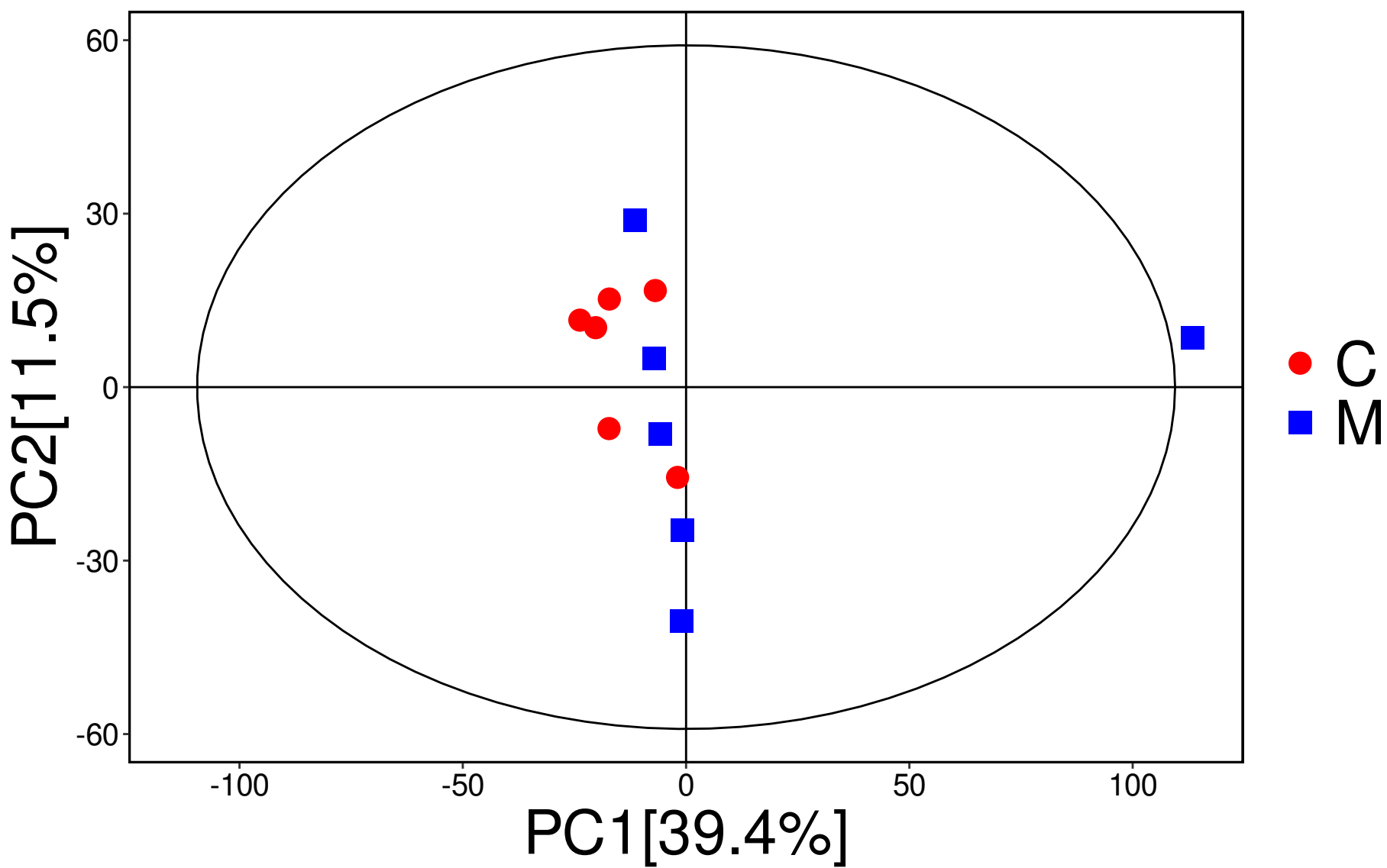

Supplement: S1 File — (ZIP) [file pone.0325562.s001.zip › S1_File/Metabolomic analysis/Statistical Analysis/TOTAL/PCA score plot.pdf]

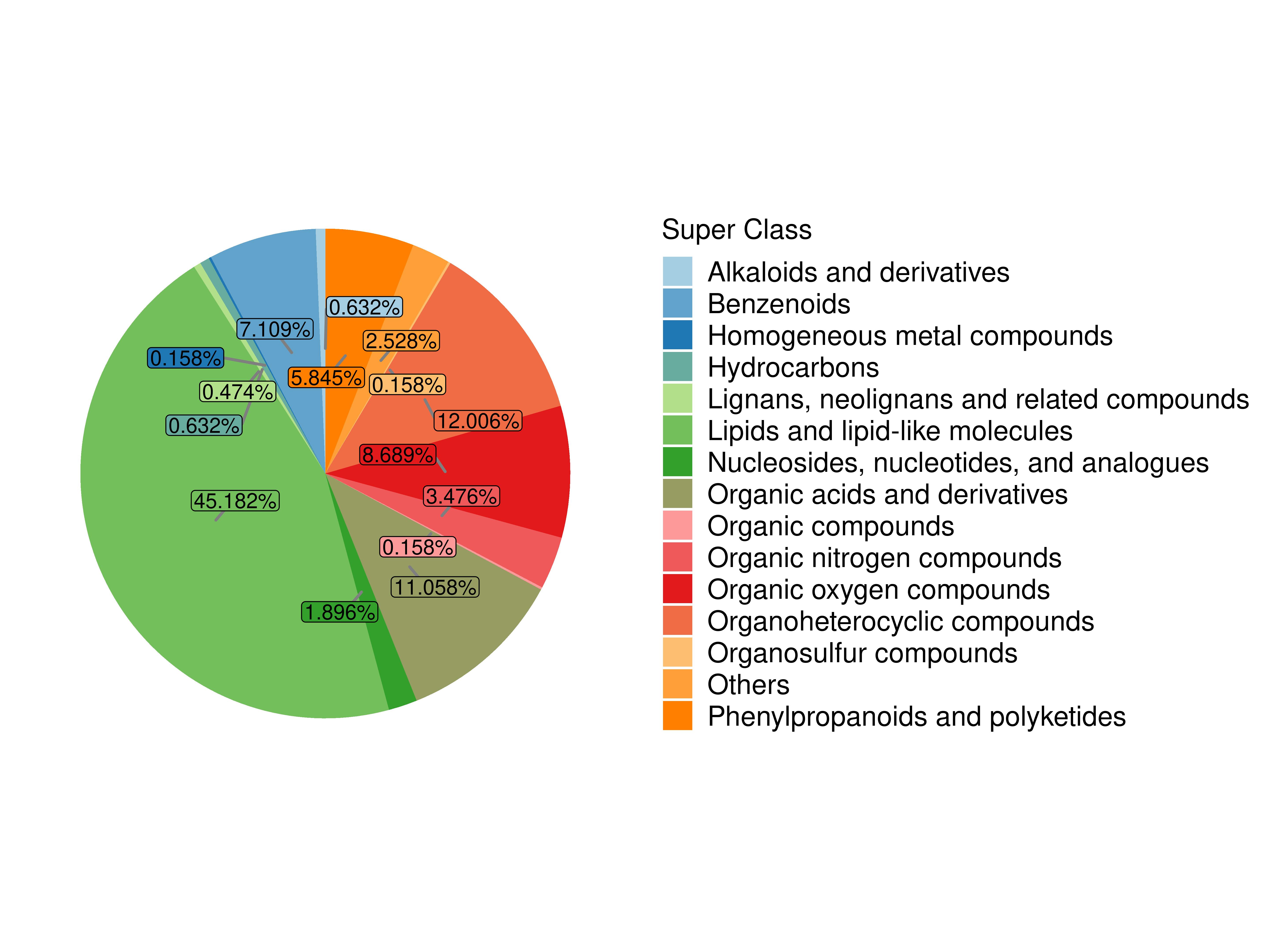

Supplement: S1 File — (ZIP) [file pone.0325562.s001.zip › S1_File/Metabolomic analysis/Statistical Analysis/TOTAL/pie plot.jpg]

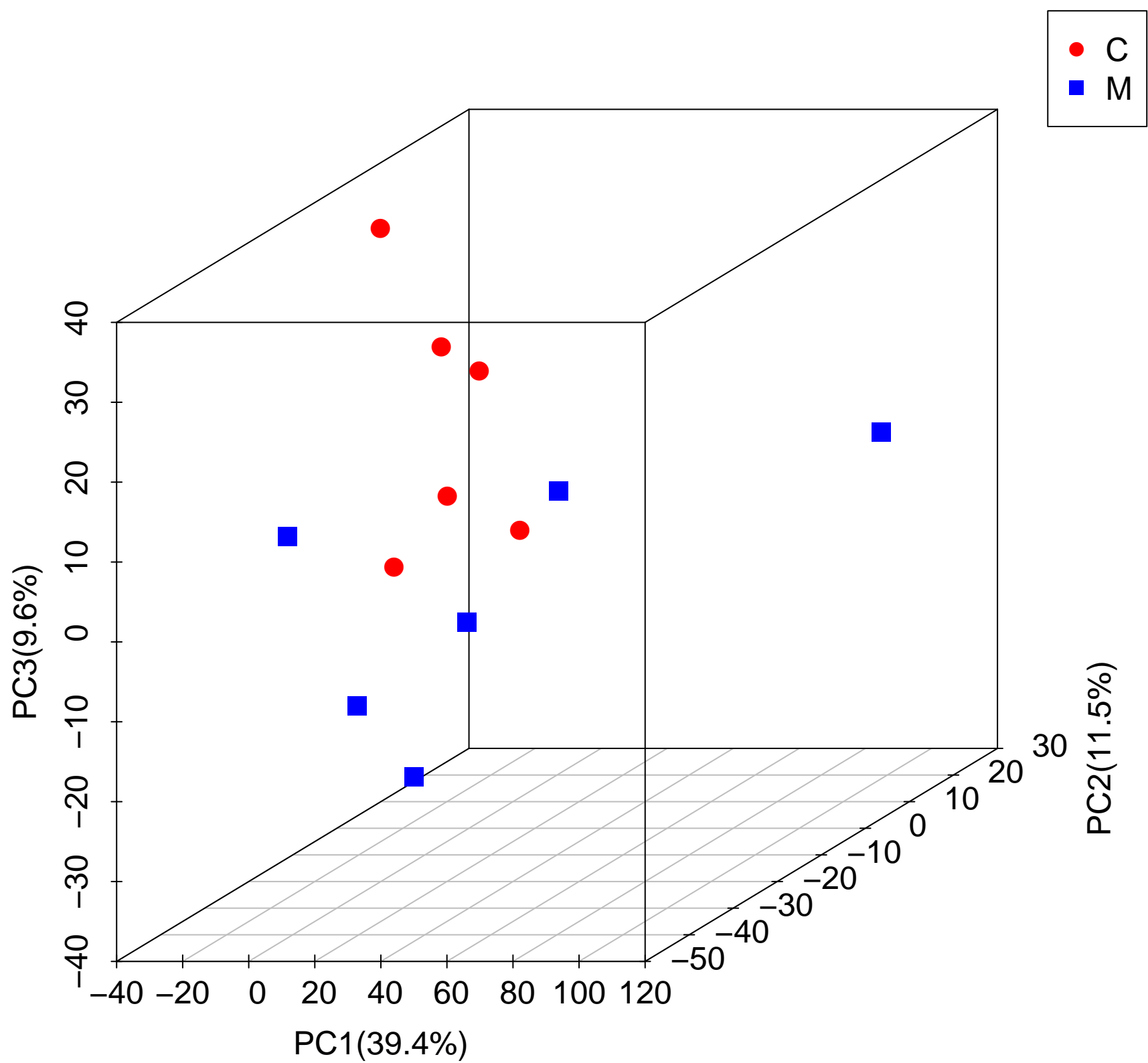

Supplement: S1 File — (ZIP) [file pone.0325562.s001.zip › S1_File/Metabolomic analysis/Statistical Analysis/TOTAL/PCA score plot 3D.pdf]

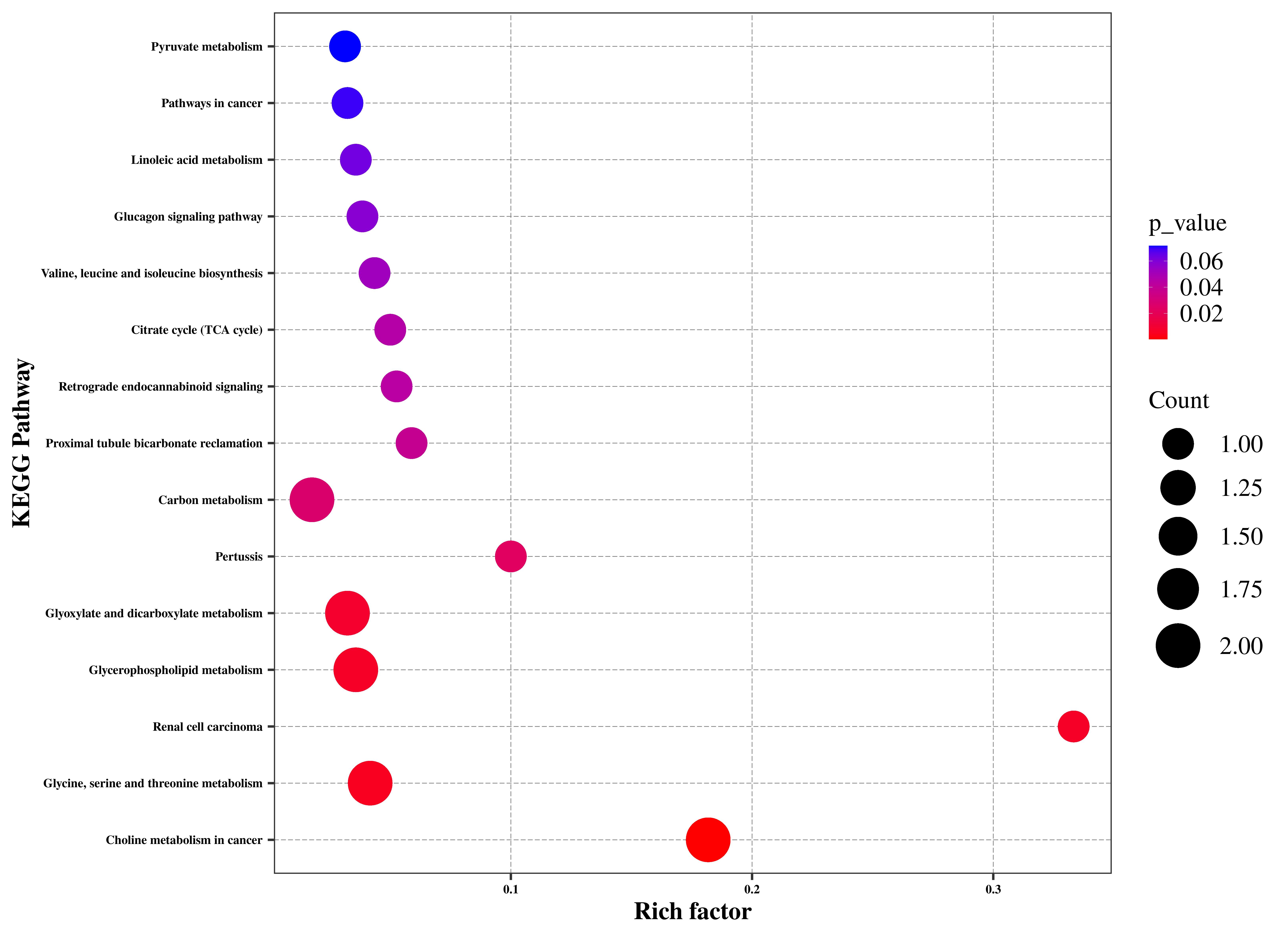

Supplement: S1 File — (ZIP) [file pone.0325562.s001.zip › S1_File/Metabolomic analysis/Enrichment Analysis/C-M/KEGG Enrichment bubble.jpg]

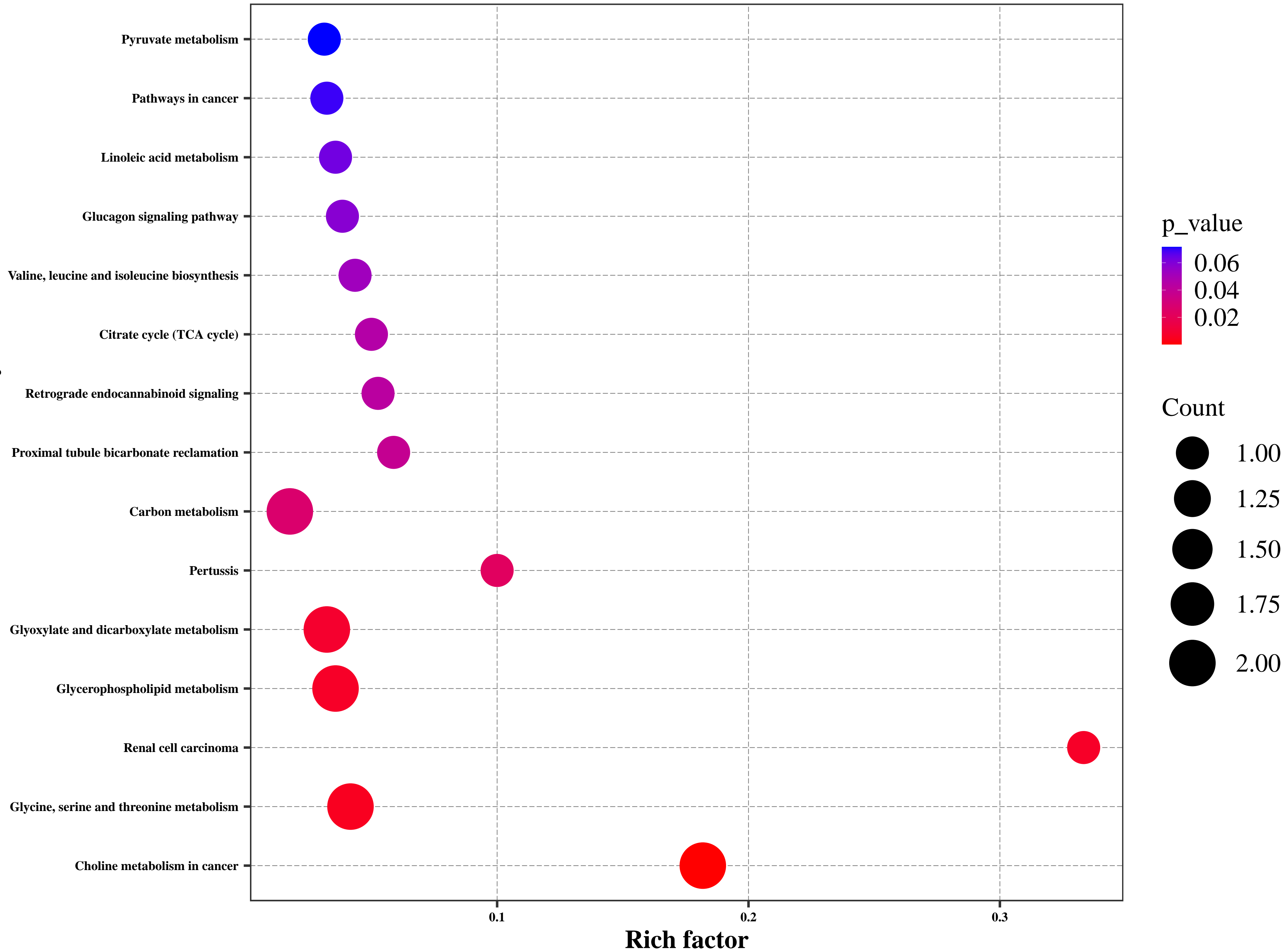

Supplement: S1 File — (ZIP) [file pone.0325562.s001.zip › S1_File/Metabolomic analysis/Enrichment Analysis/C-M/KEGG Enrichment bubble.pdf]

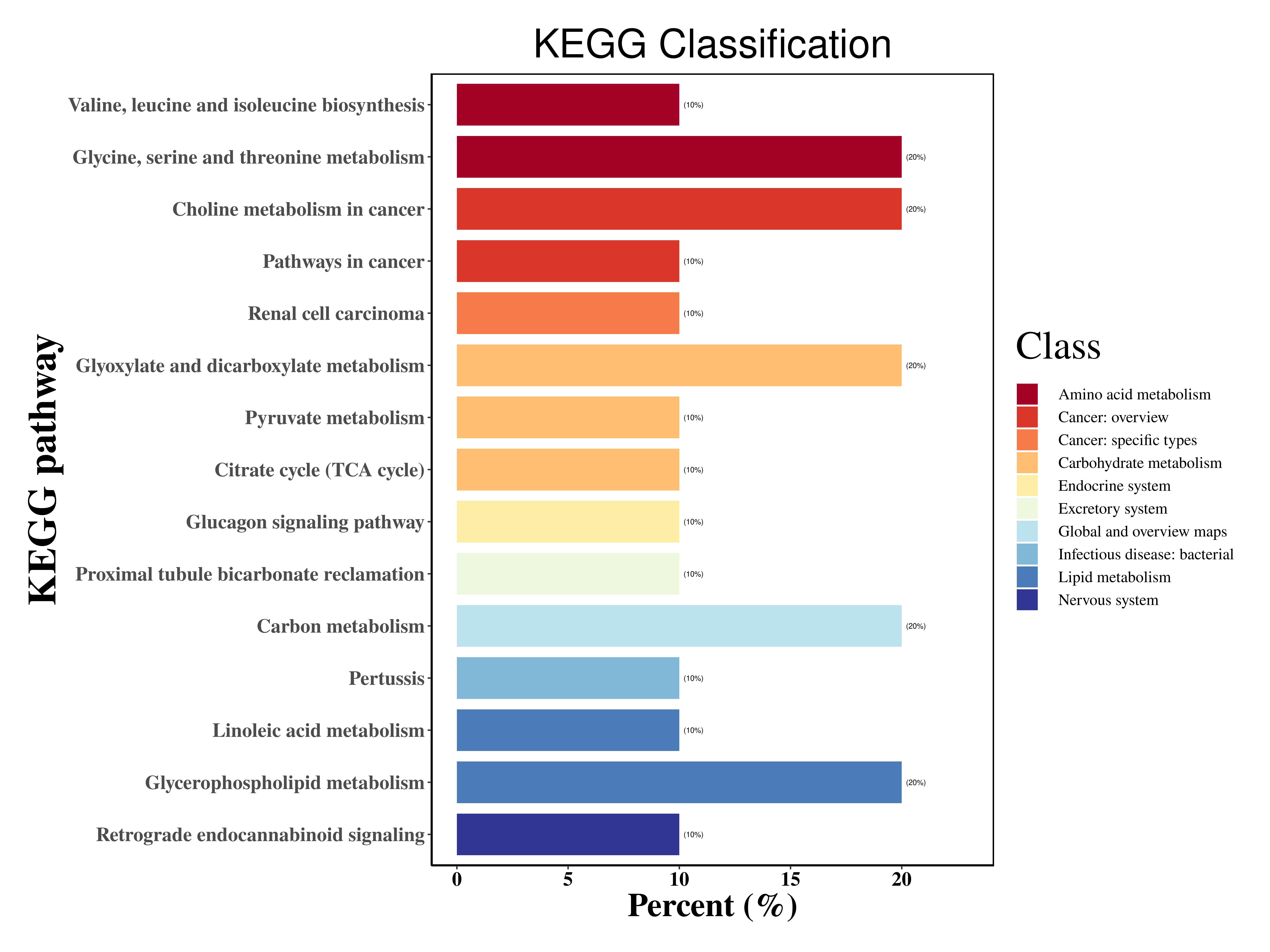

Supplement: S1 File — (ZIP) [file pone.0325562.s001.zip › S1_File/Metabolomic analysis/Enrichment Analysis/C-M/KEGG Classification.png]

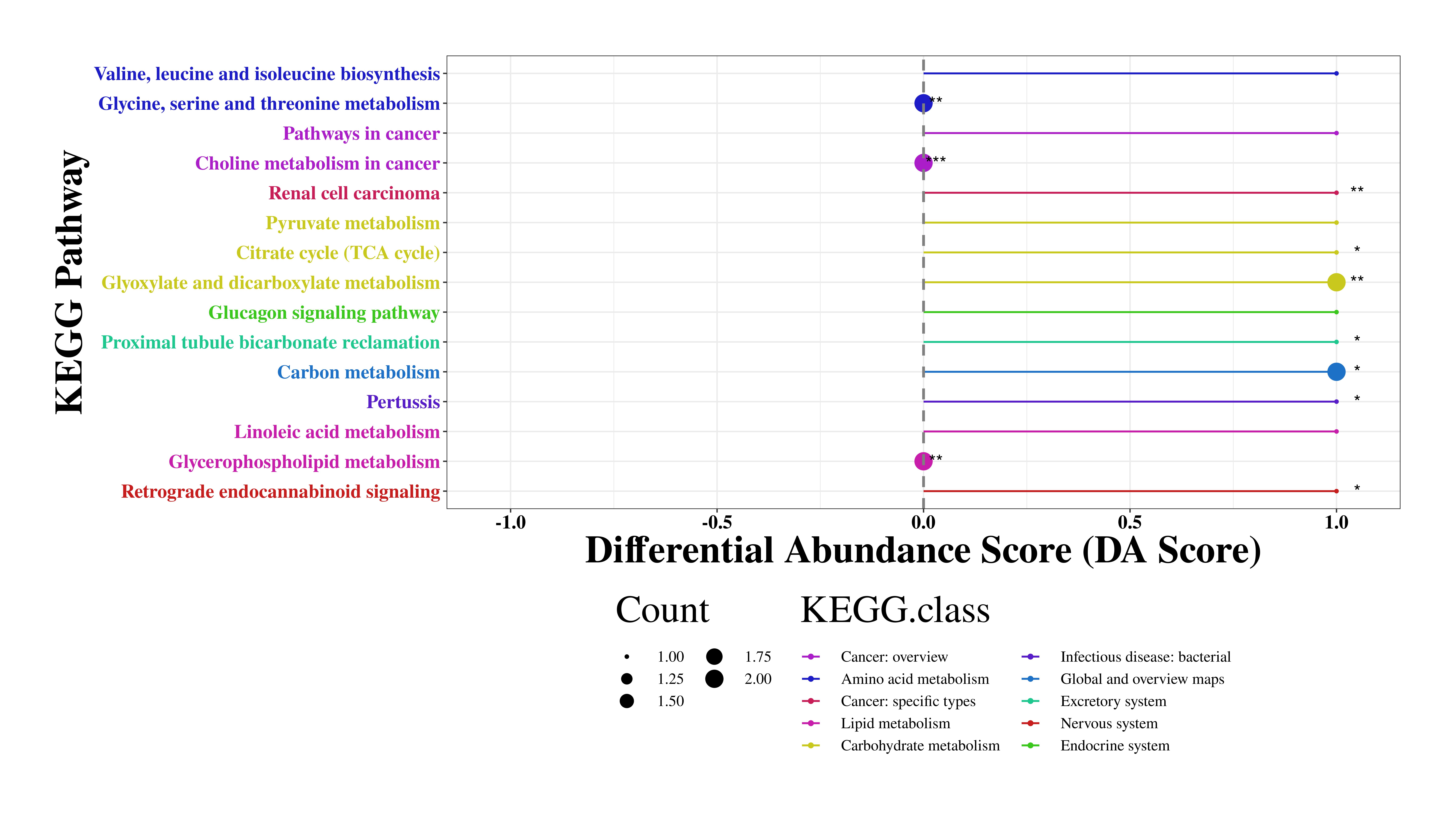

Supplement: S1 File — (ZIP) [file pone.0325562.s001.zip › S1_File/Metabolomic analysis/Enrichment Analysis/C-M/KEGG DA Score plot.jpg]

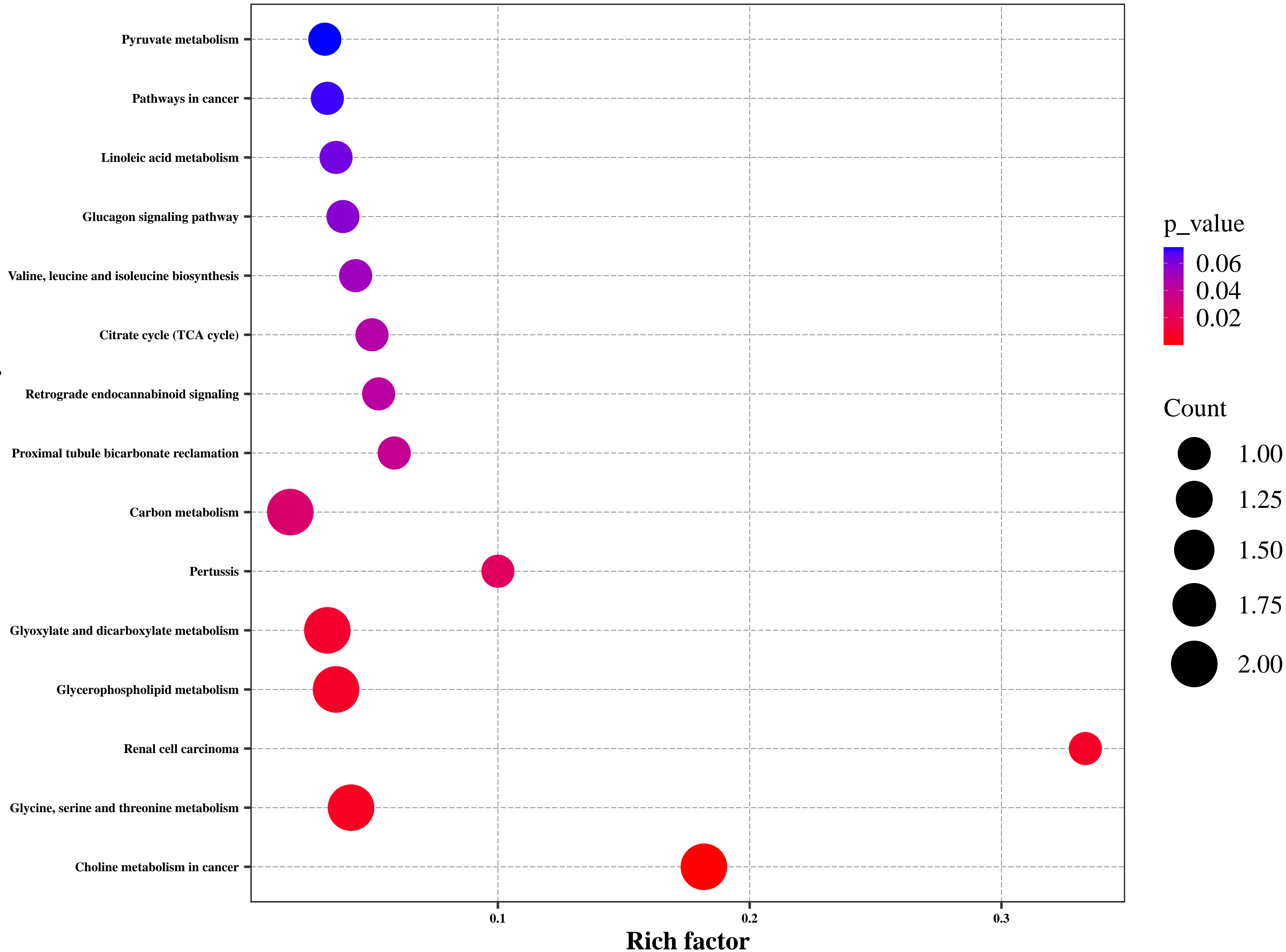

Supplement: S1 File — (ZIP) [file pone.0325562.s001.zip › S1_File/Metabolomic analysis/Enrichment Analysis/M-C/KEGG Enrichment bubble.pdf]

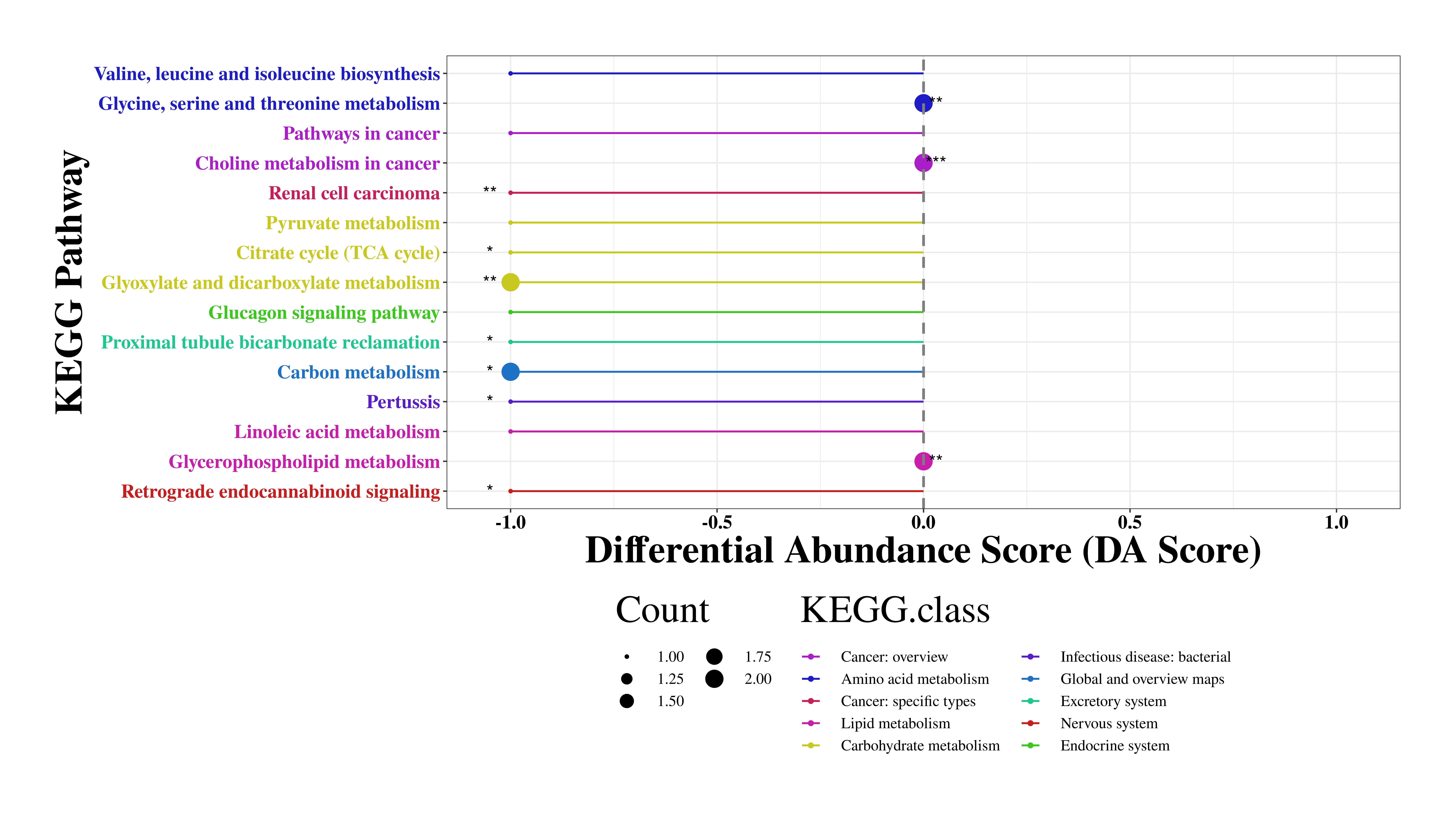

Supplement: S1 File — (ZIP) [file pone.0325562.s001.zip › S1_File/Metabolomic analysis/Enrichment Analysis/M-C/KEGG DA Score plot.jpg]

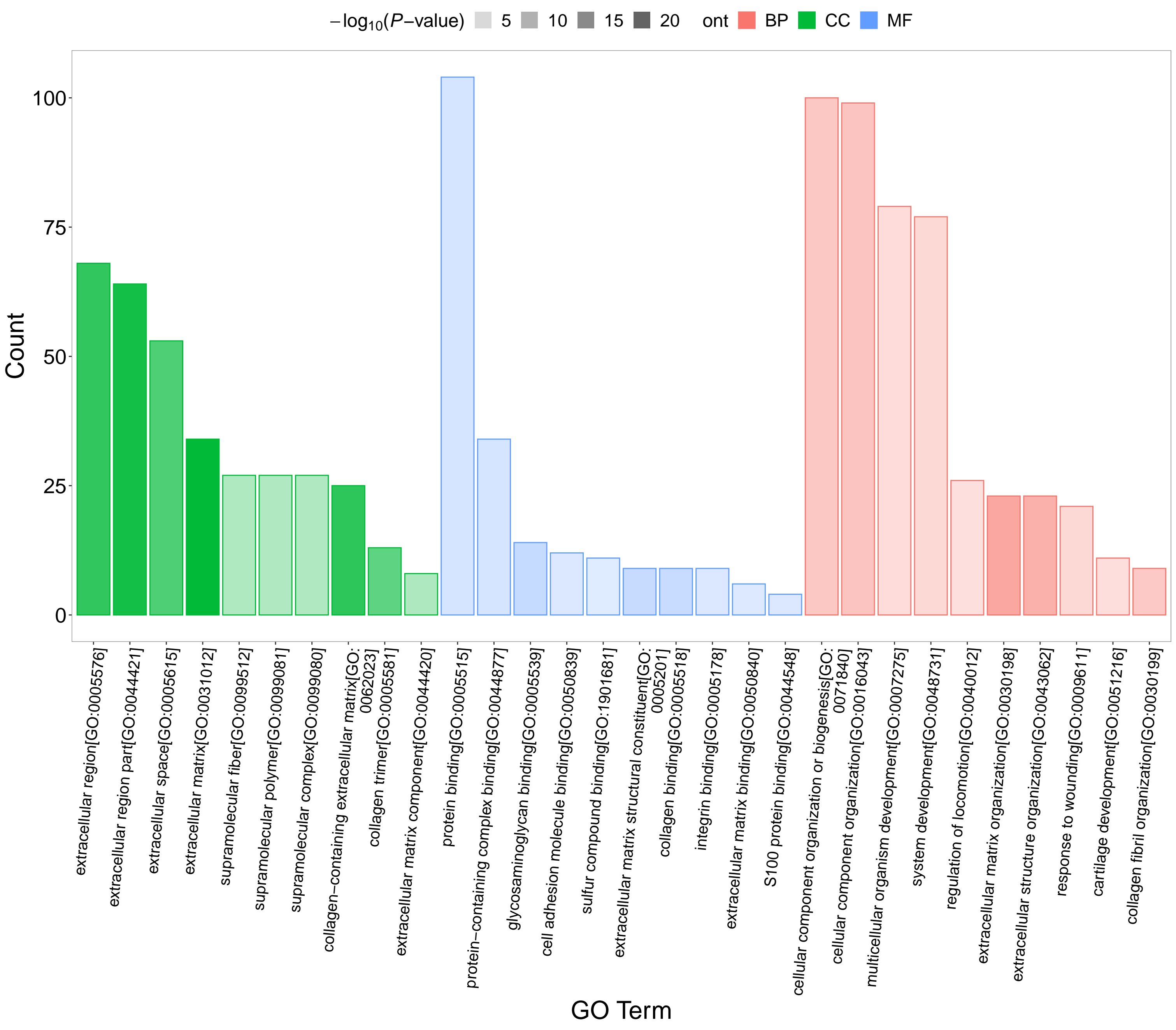

Supplement: S1 File — (ZIP) [file pone.0325562.s001.zip › S1_File/Proteomics analysis/GO Annotation Enrichment Analysis/M_vs_C/barplot.pdf]

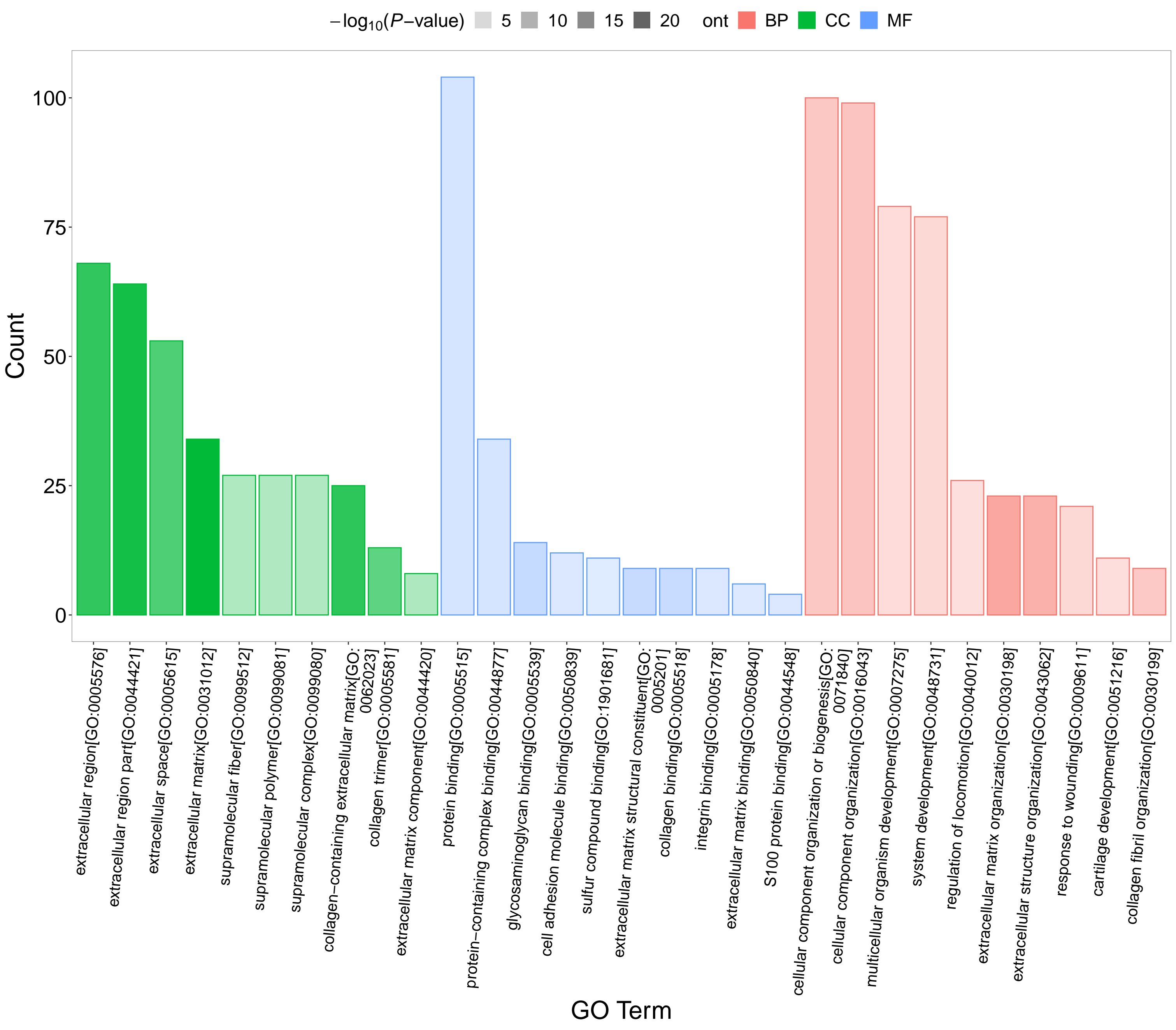

Supplement: S1 File — (ZIP) [file pone.0325562.s001.zip › S1_File/Proteomics analysis/GO Annotation Enrichment Analysis/C_vs_M/barplot.pdf]

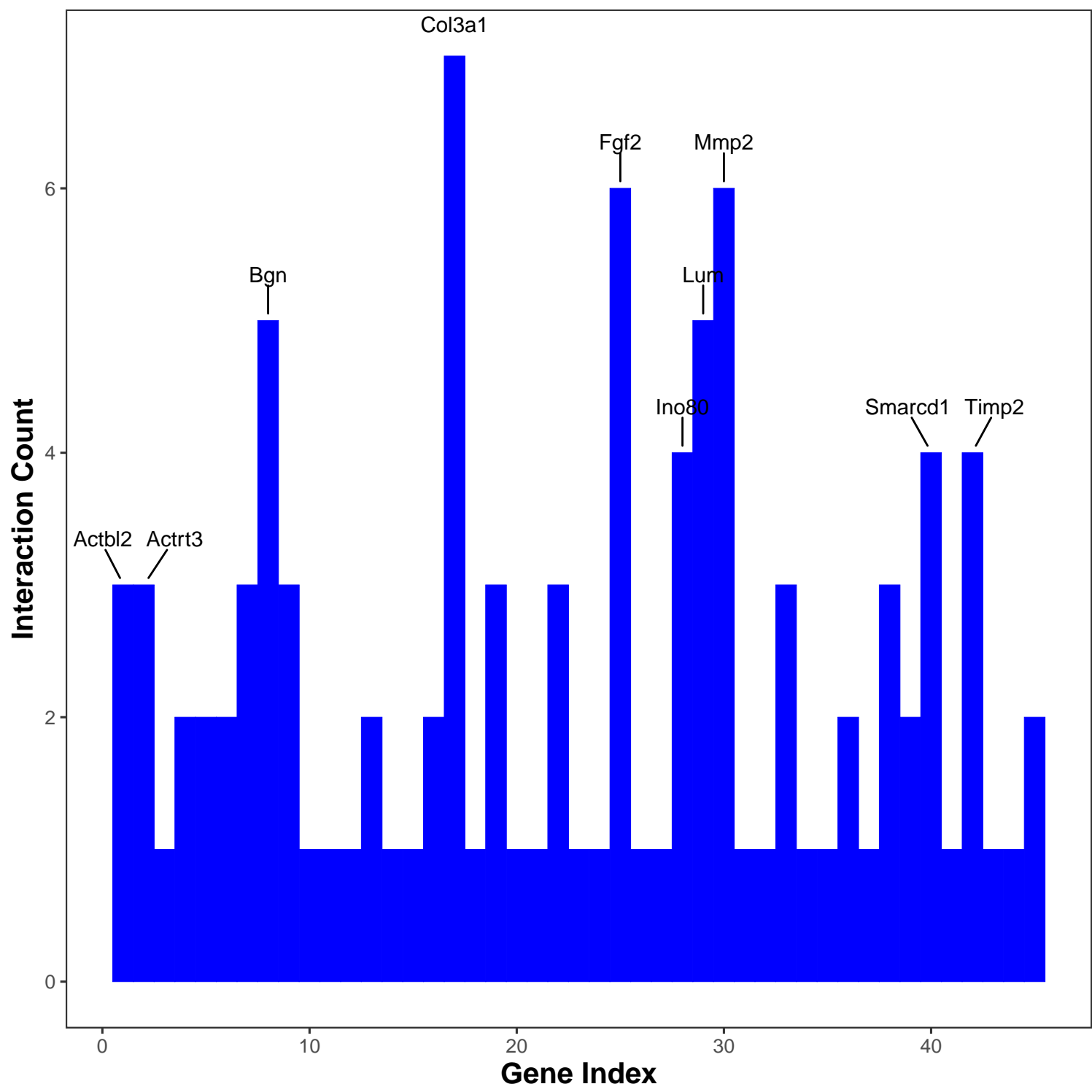

Supplement: S1 File — (ZIP) [file pone.0325562.s001.zip › S1_File/Proteomics analysis/PPI Network Analysis/M_vs_C/degrees.pdf]

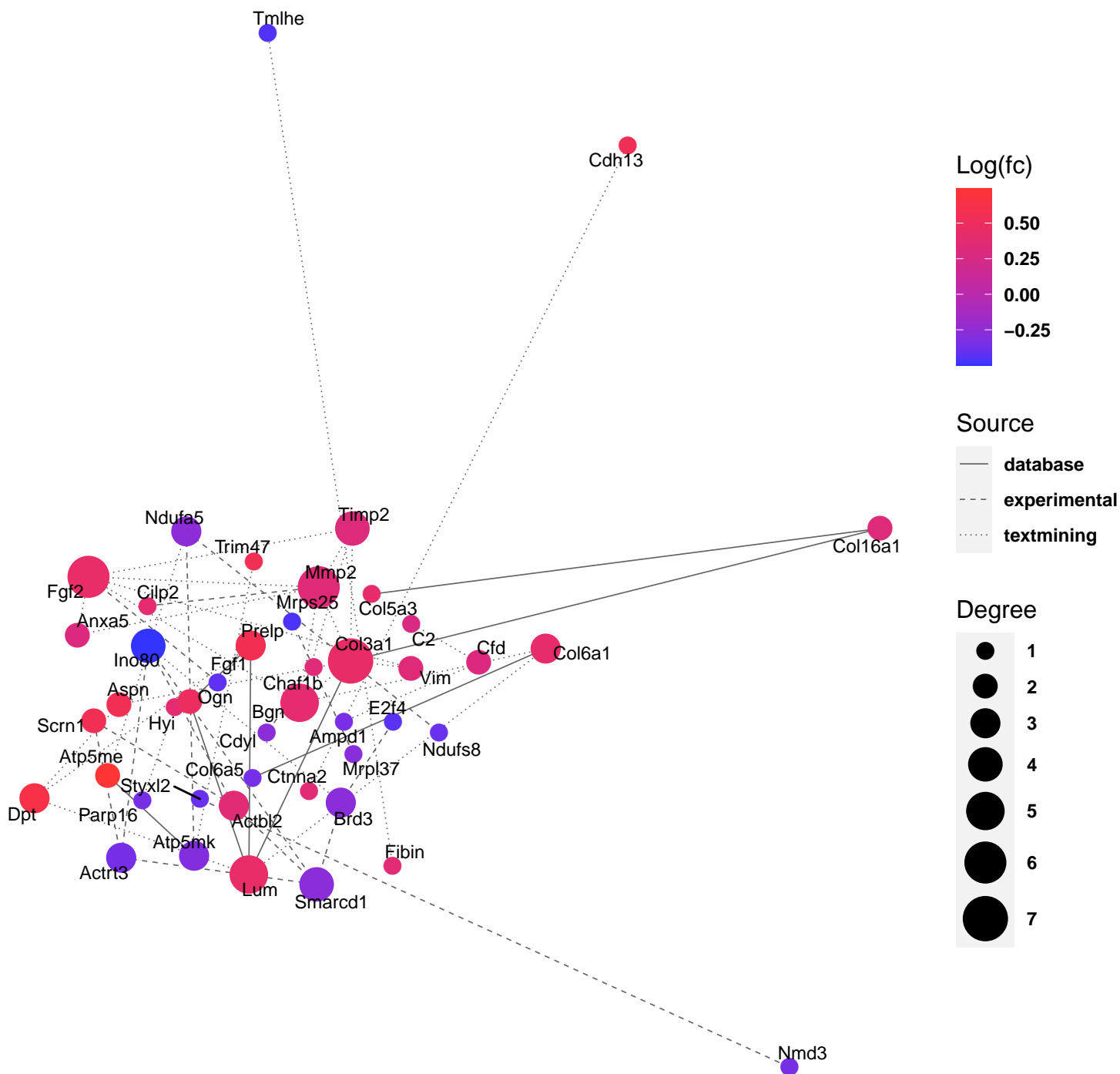

Supplement: S1 File — (ZIP) [file pone.0325562.s001.zip › S1_File/Proteomics analysis/PPI Network Analysis/M_vs_C/Network.pdf]

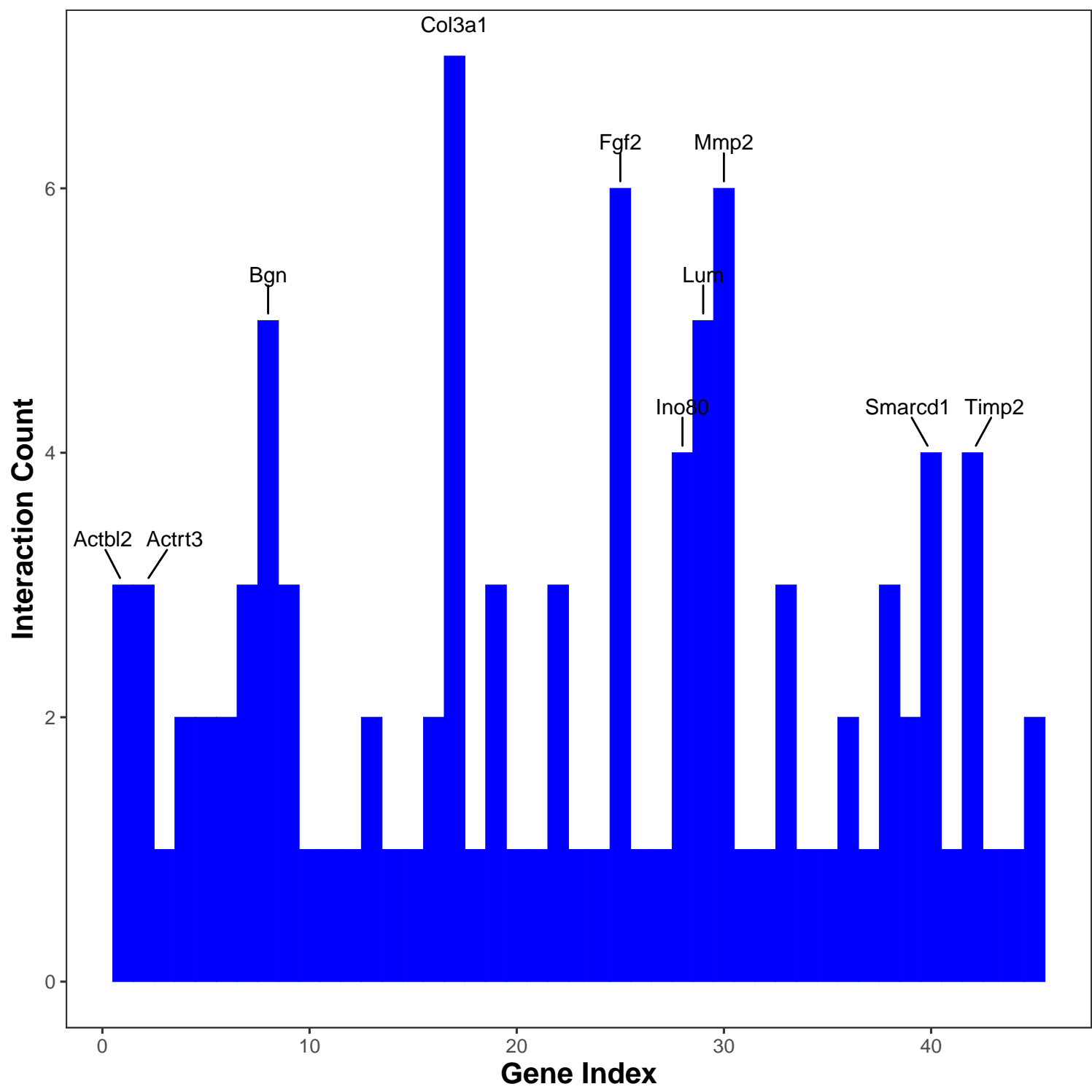

Supplement: S1 File — (ZIP) [file pone.0325562.s001.zip › S1_File/Proteomics analysis/PPI Network Analysis/C_vs_M/degrees.pdf]

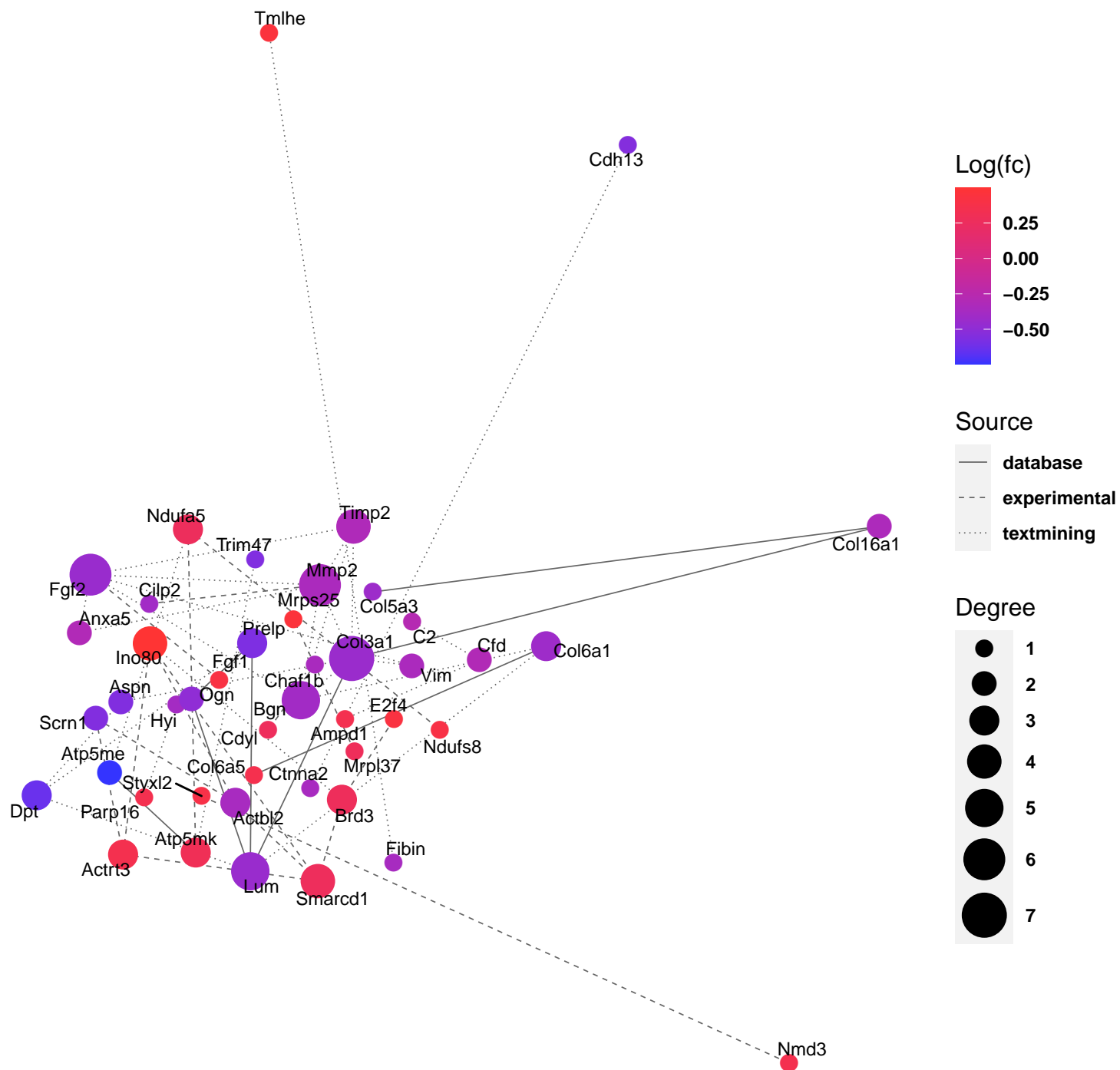

Supplement: S1 File — (ZIP) [file pone.0325562.s001.zip › S1_File/Proteomics analysis/PPI Network Analysis/C_vs_M/Network.pdf]

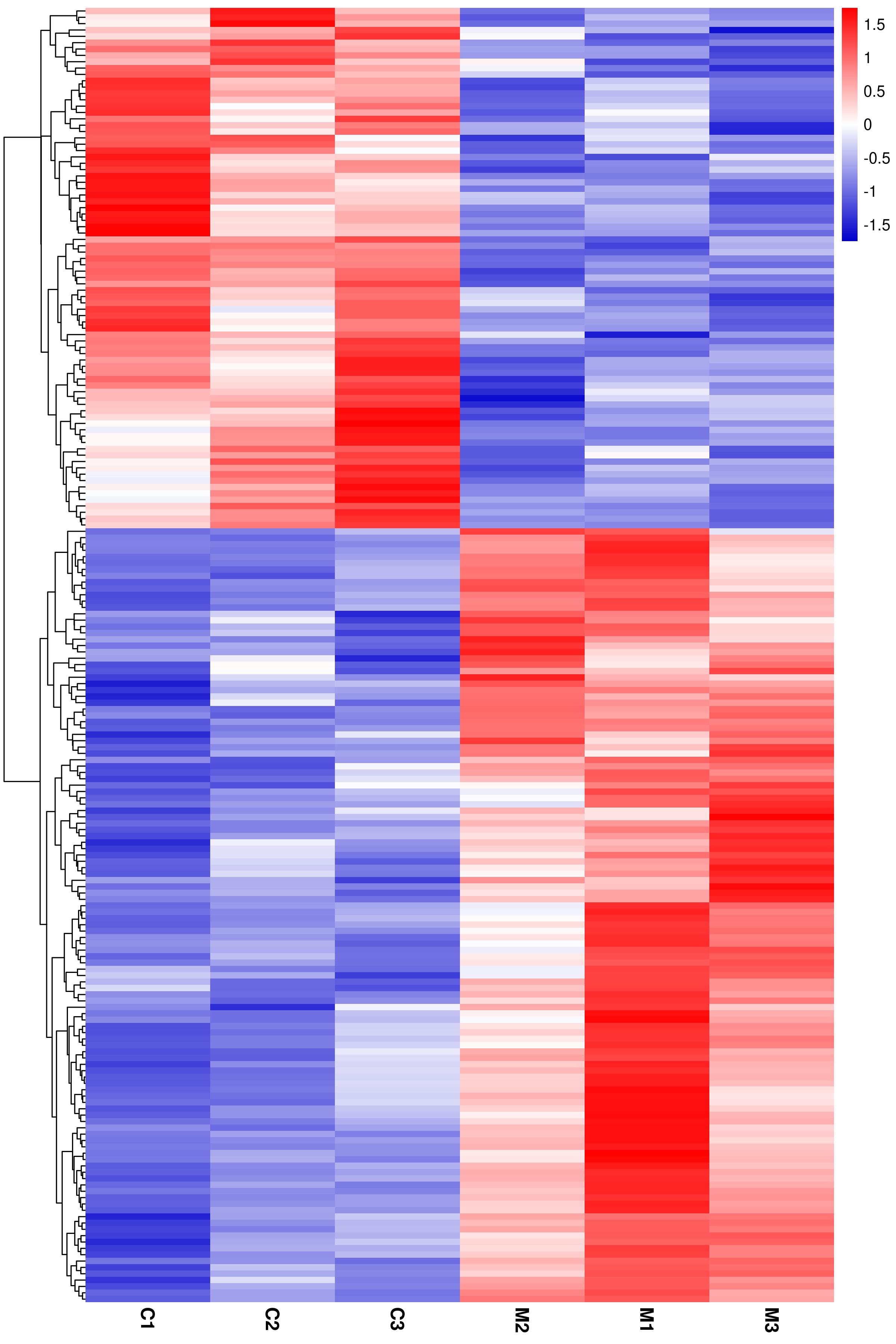

Supplement: S1 File — (ZIP) [file pone.0325562.s001.zip › S1_File/Proteomics analysis/Hierarchical Clustering Analysis/M_vs_C/heatmap_no_label.jpg]

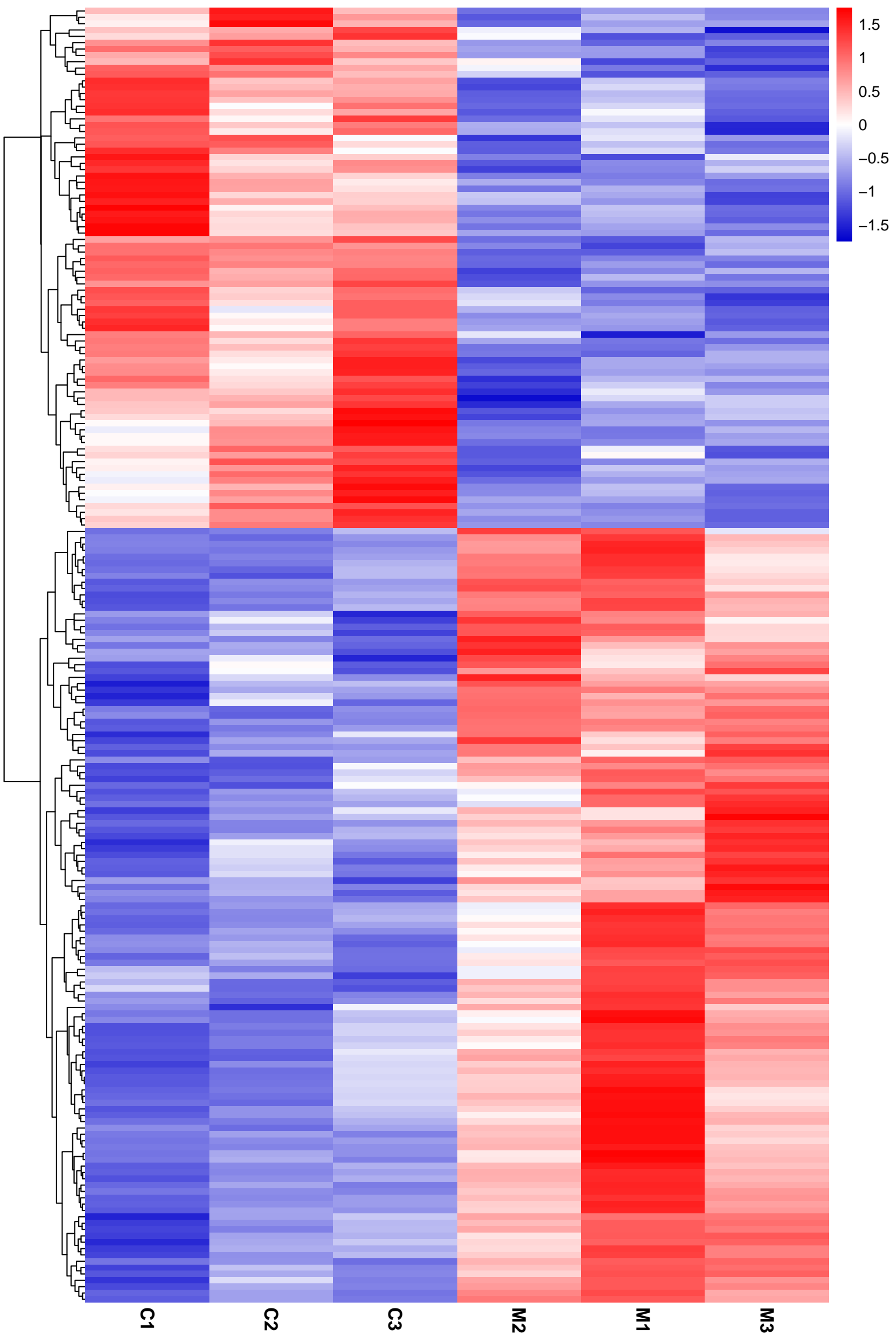

Supplement: S1 File — (ZIP) [file pone.0325562.s001.zip › S1_File/Proteomics analysis/Hierarchical Clustering Analysis/M_vs_C/heatmap_no_label.pdf]

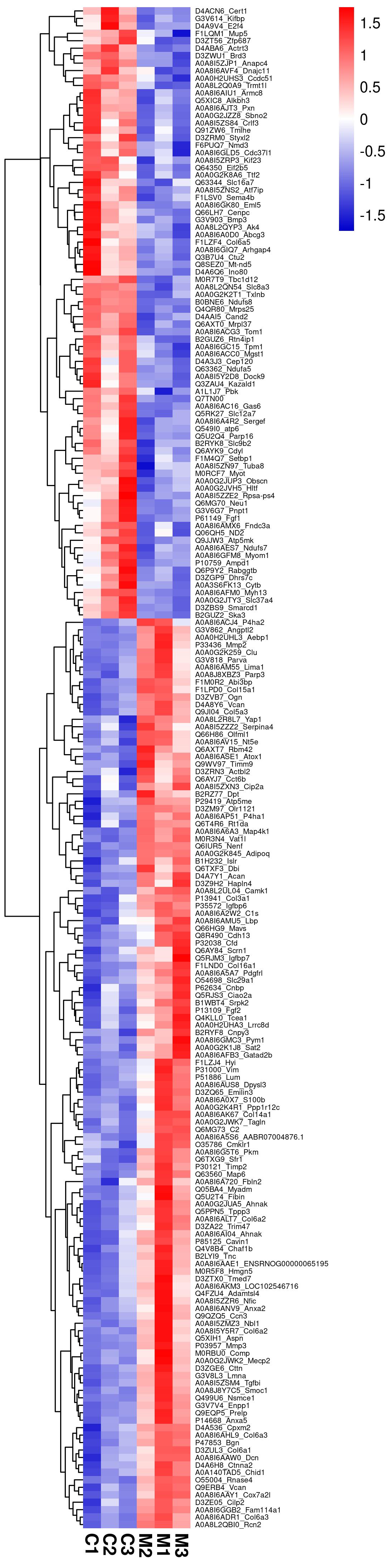

Supplement: S1 File — (ZIP) [file pone.0325562.s001.zip › S1_File/Proteomics analysis/Hierarchical Clustering Analysis/M_vs_C/heatmap.jpg]

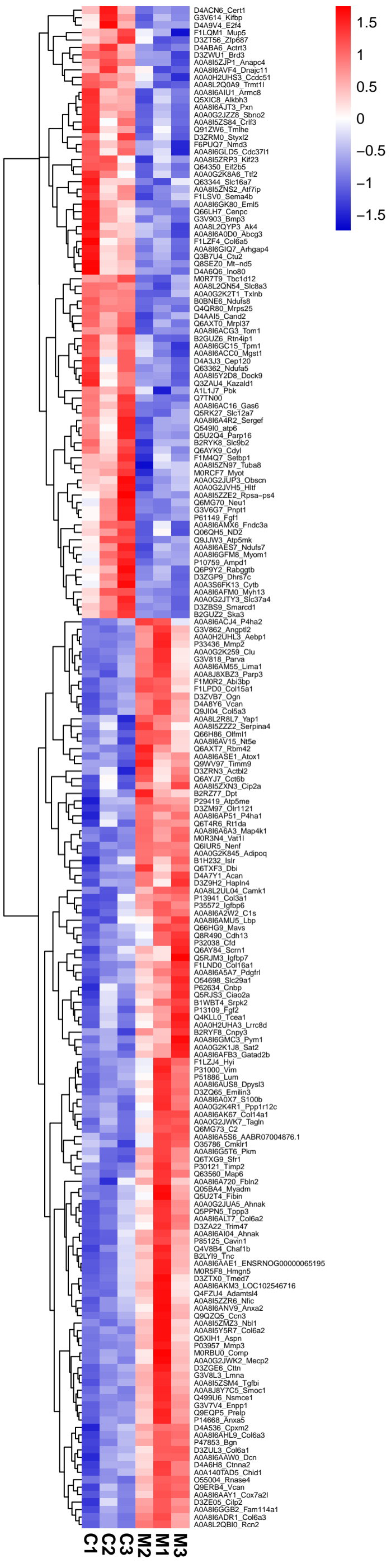

Supplement: S1 File — (ZIP) [file pone.0325562.s001.zip › S1_File/Proteomics analysis/Hierarchical Clustering Analysis/M_vs_C/heatmap.pdf]

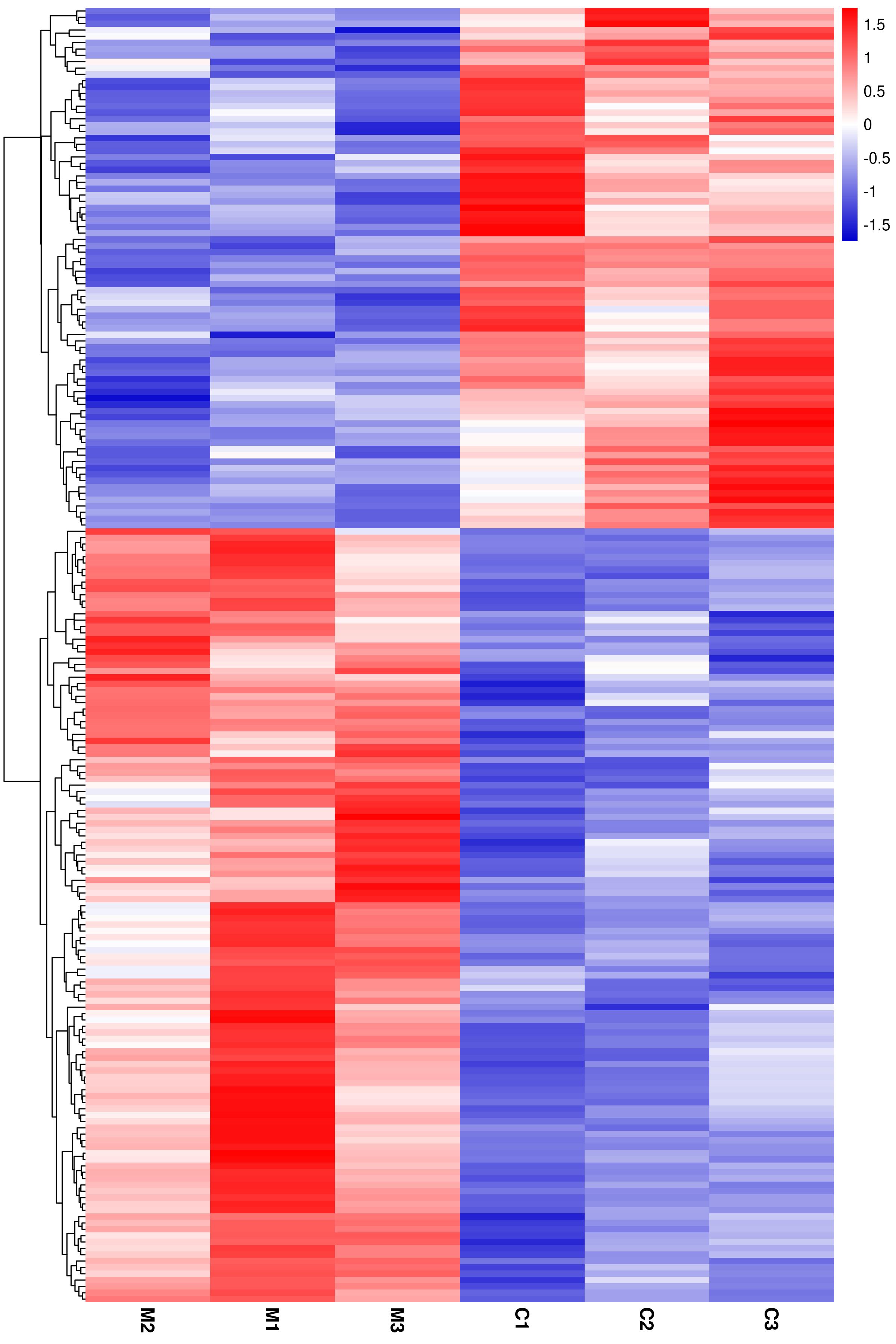

Supplement: S1 File — (ZIP) [file pone.0325562.s001.zip › S1_File/Proteomics analysis/Hierarchical Clustering Analysis/C_vs_M/heatmap_no_label.jpg]

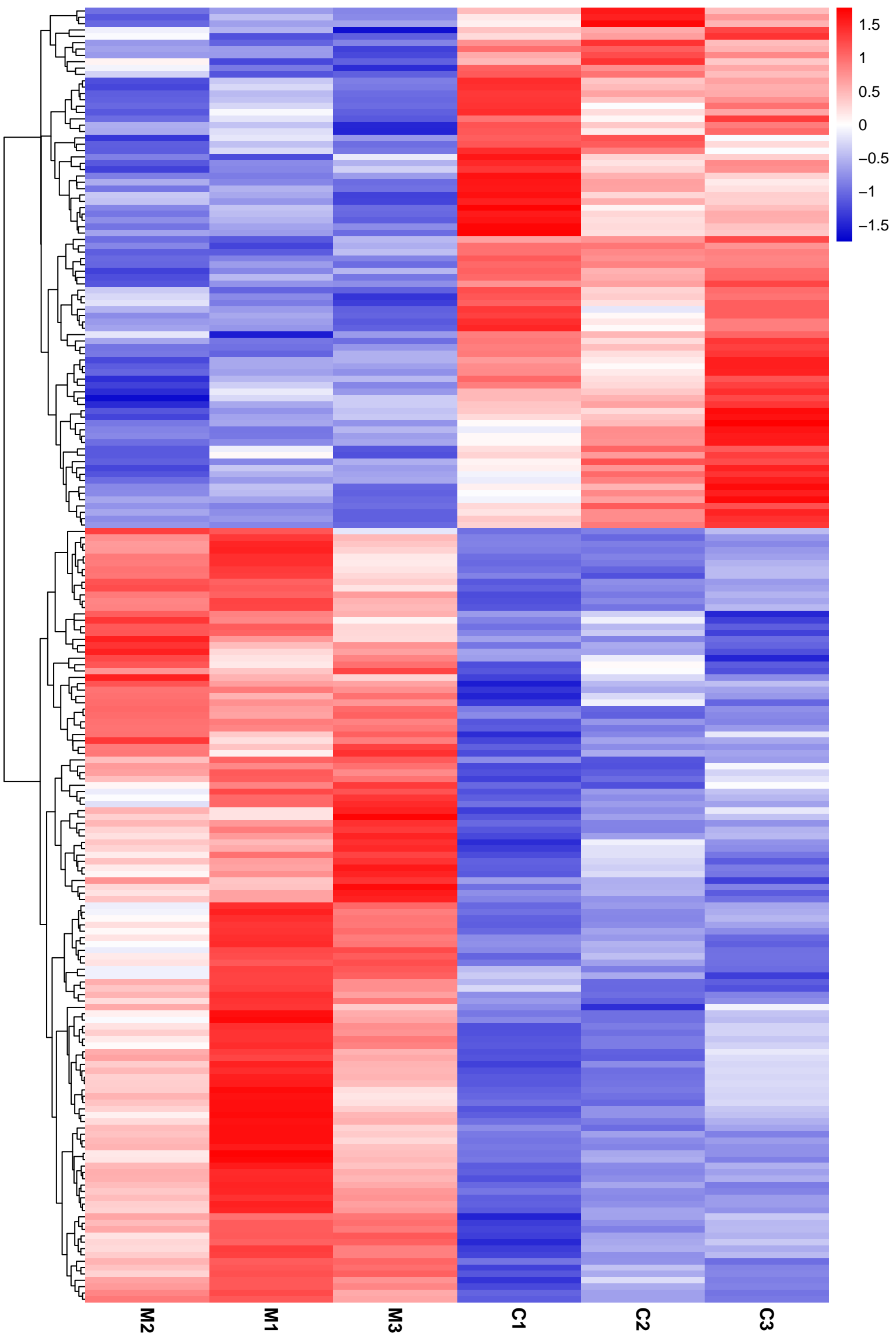

Supplement: S1 File — (ZIP) [file pone.0325562.s001.zip › S1_File/Proteomics analysis/Hierarchical Clustering Analysis/C_vs_M/heatmap_no_label.pdf]

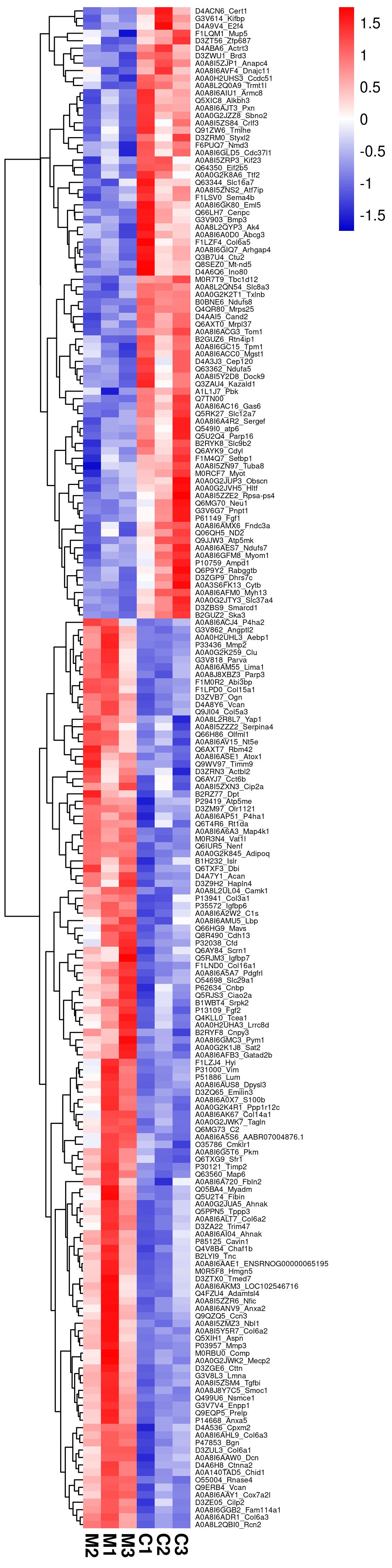

Supplement: S1 File — (ZIP) [file pone.0325562.s001.zip › S1_File/Proteomics analysis/Hierarchical Clustering Analysis/C_vs_M/heatmap.jpg]

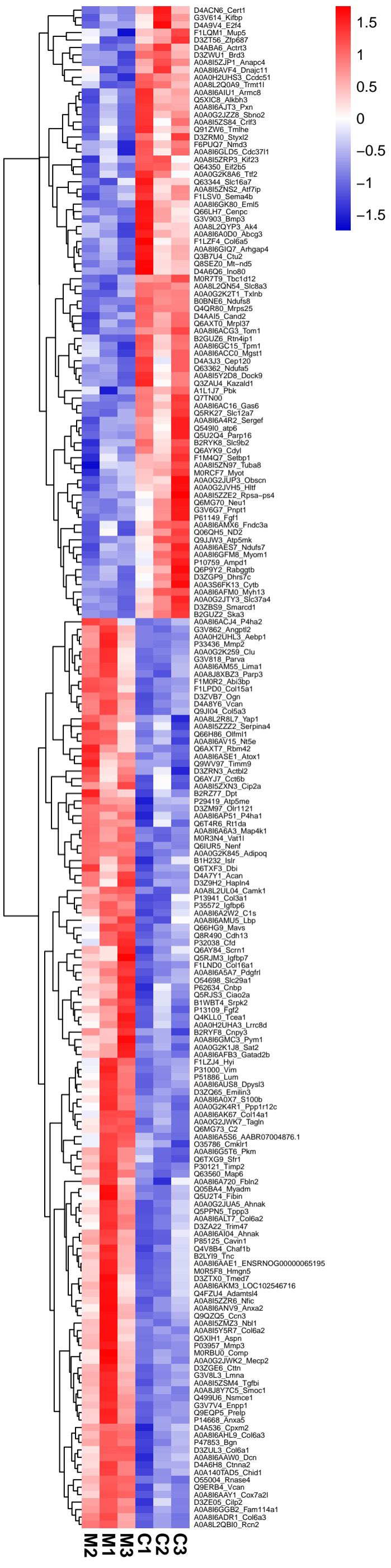

Supplement: S1 File — (ZIP) [file pone.0325562.s001.zip › S1_File/Proteomics analysis/Hierarchical Clustering Analysis/C_vs_M/heatmap.pdf]

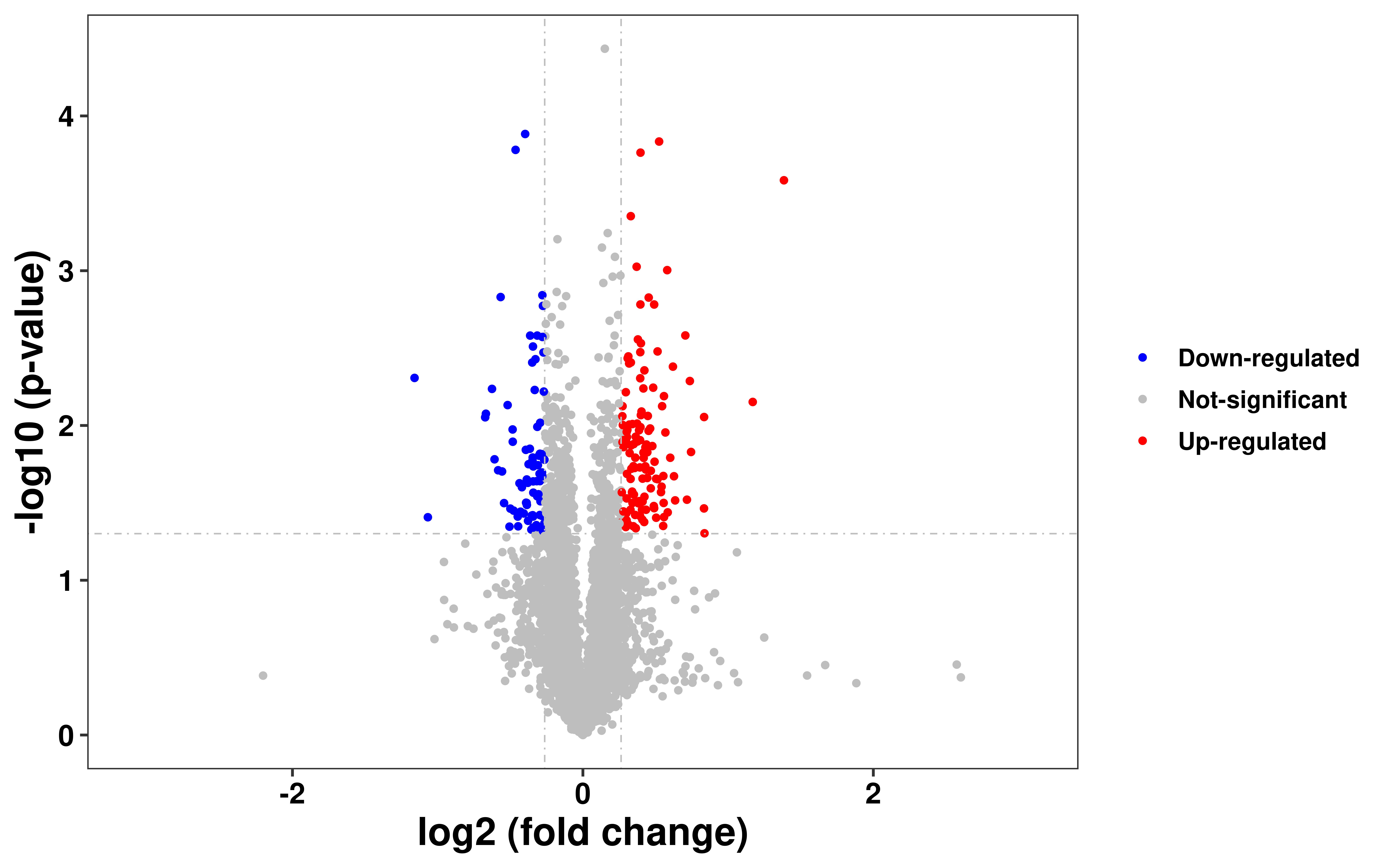

Supplement: S1 File — (ZIP) [file pone.0325562.s001.zip › S1_File/Proteomics analysis/Volcano Analysis/M_vs_C/volcano.jpg]

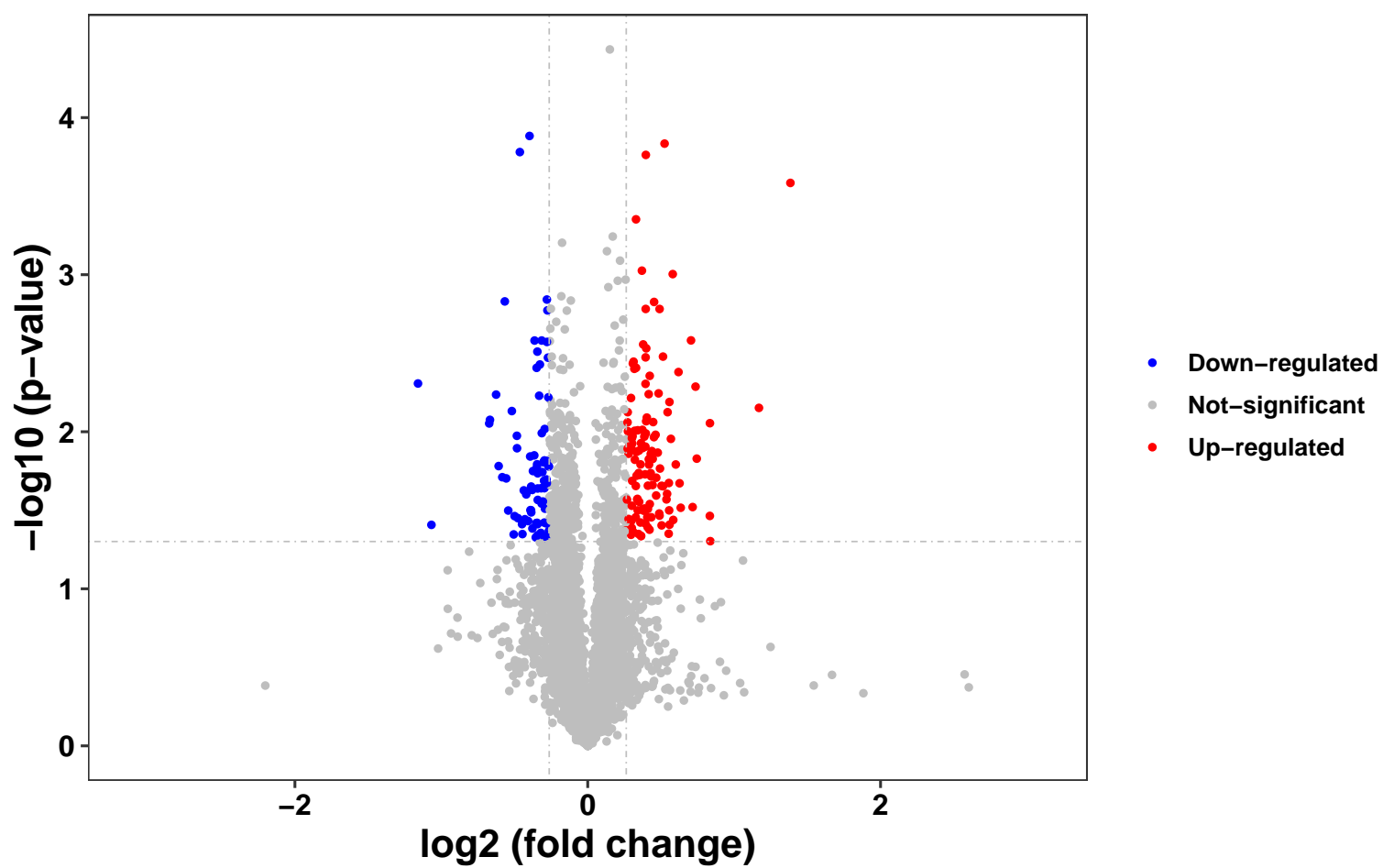

Supplement: S1 File — (ZIP) [file pone.0325562.s001.zip › S1_File/Proteomics analysis/Volcano Analysis/M_vs_C/volcano.pdf]

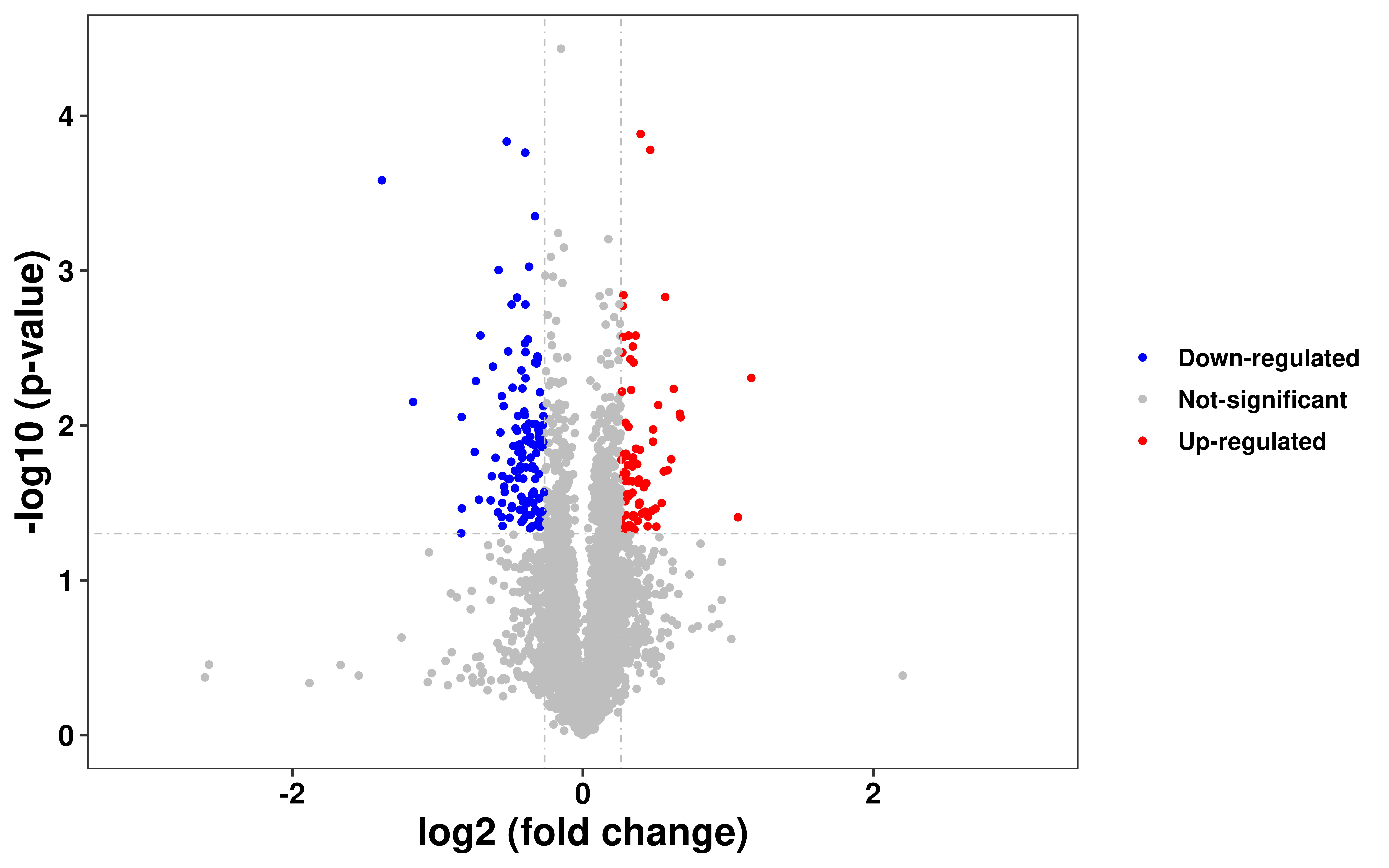

Supplement: S1 File — (ZIP) [file pone.0325562.s001.zip › S1_File/Proteomics analysis/Volcano Analysis/C_vs_M/volcano.jpg]

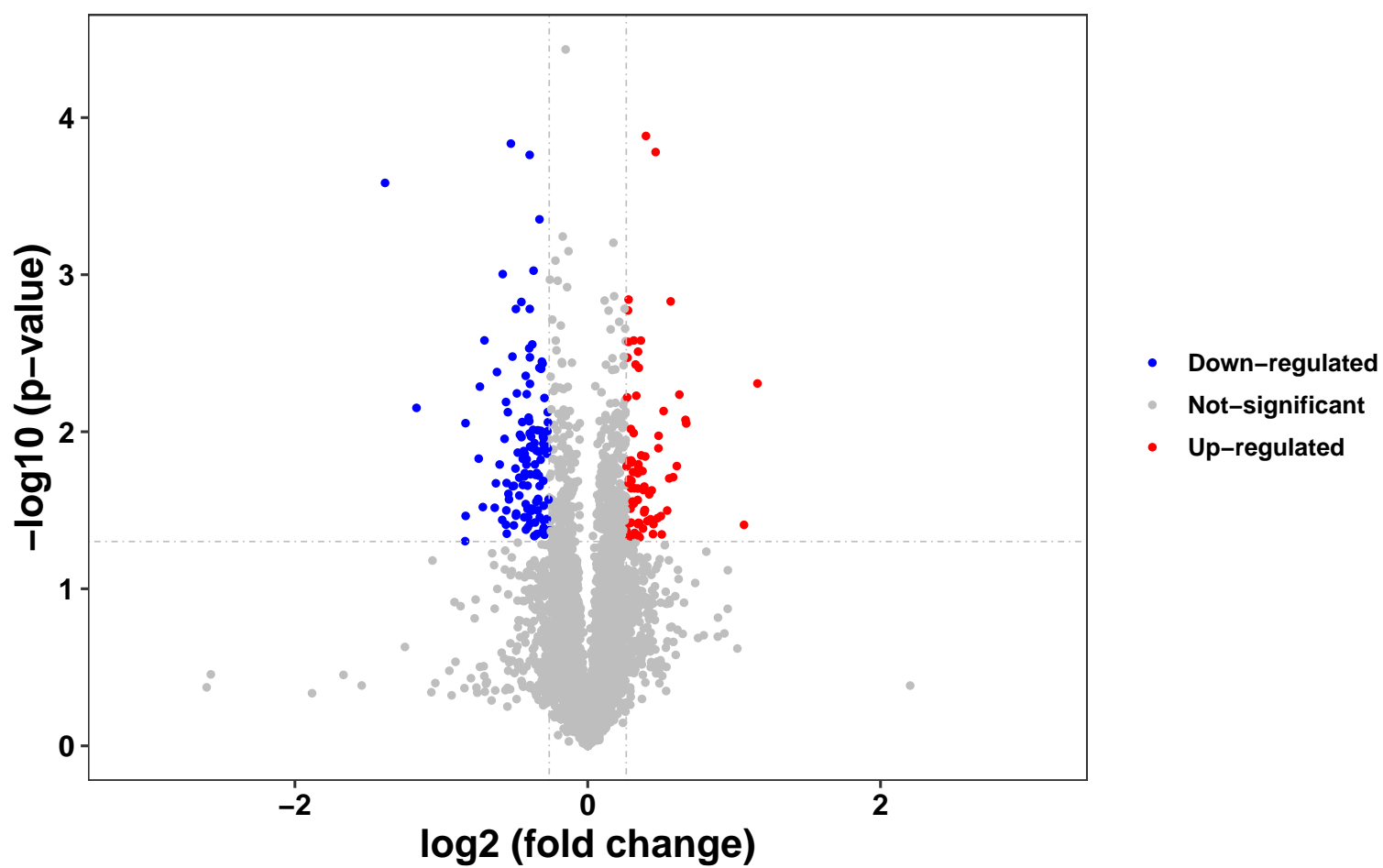

Supplement: S1 File — (ZIP) [file pone.0325562.s001.zip › S1_File/Proteomics analysis/Volcano Analysis/C_vs_M/volcano.pdf]

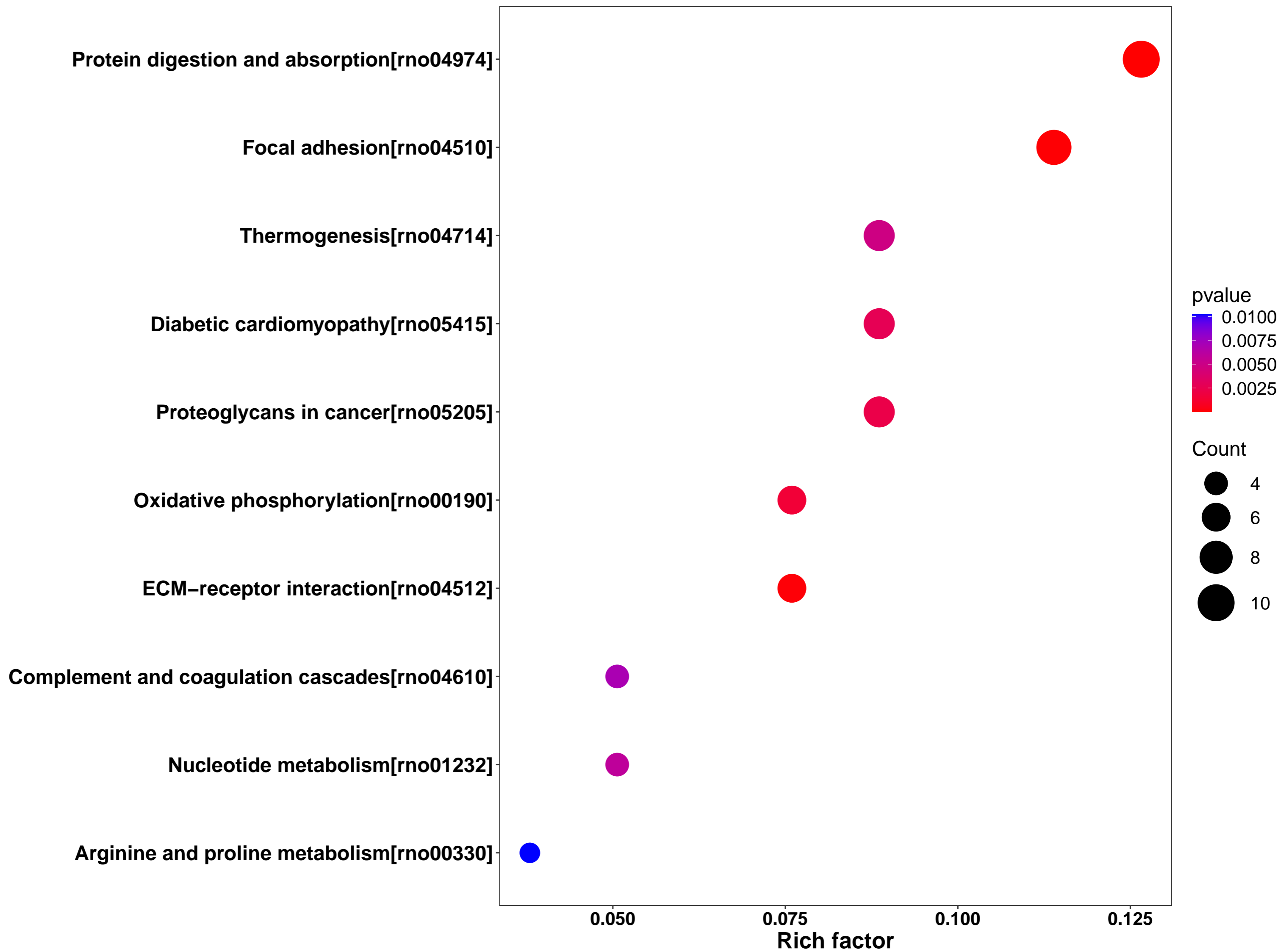

Supplement: S1 File — (ZIP) [file pone.0325562.s001.zip › S1_File/Proteomics analysis/KEGG Analysis/M_vs_C/bubble.pdf]

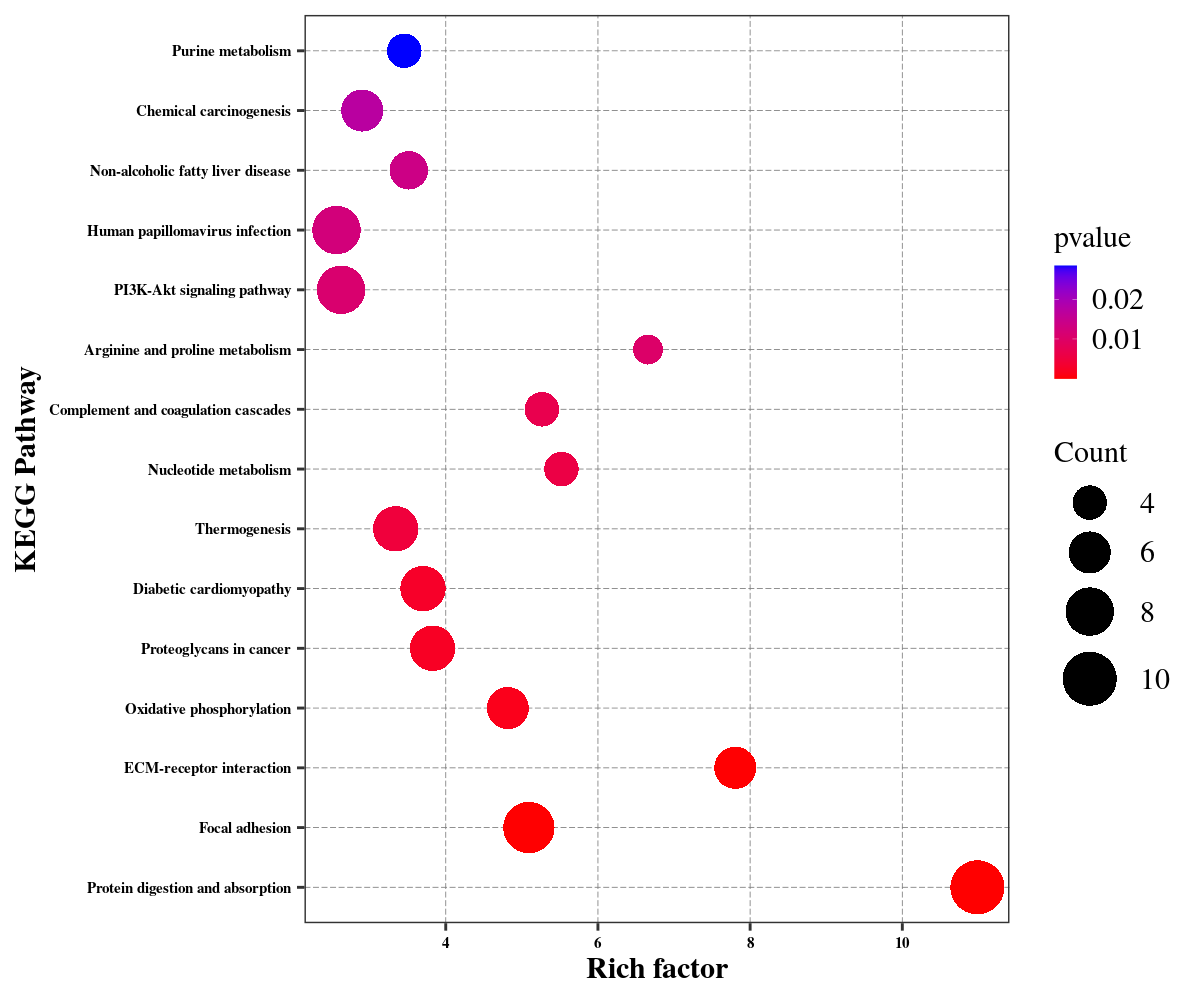

Supplement: S1 File — (ZIP) [file pone.0325562.s001.zip › S1_File/Proteomics analysis/KEGG Analysis/M_vs_C/KEGG Enrichment bubble.png]

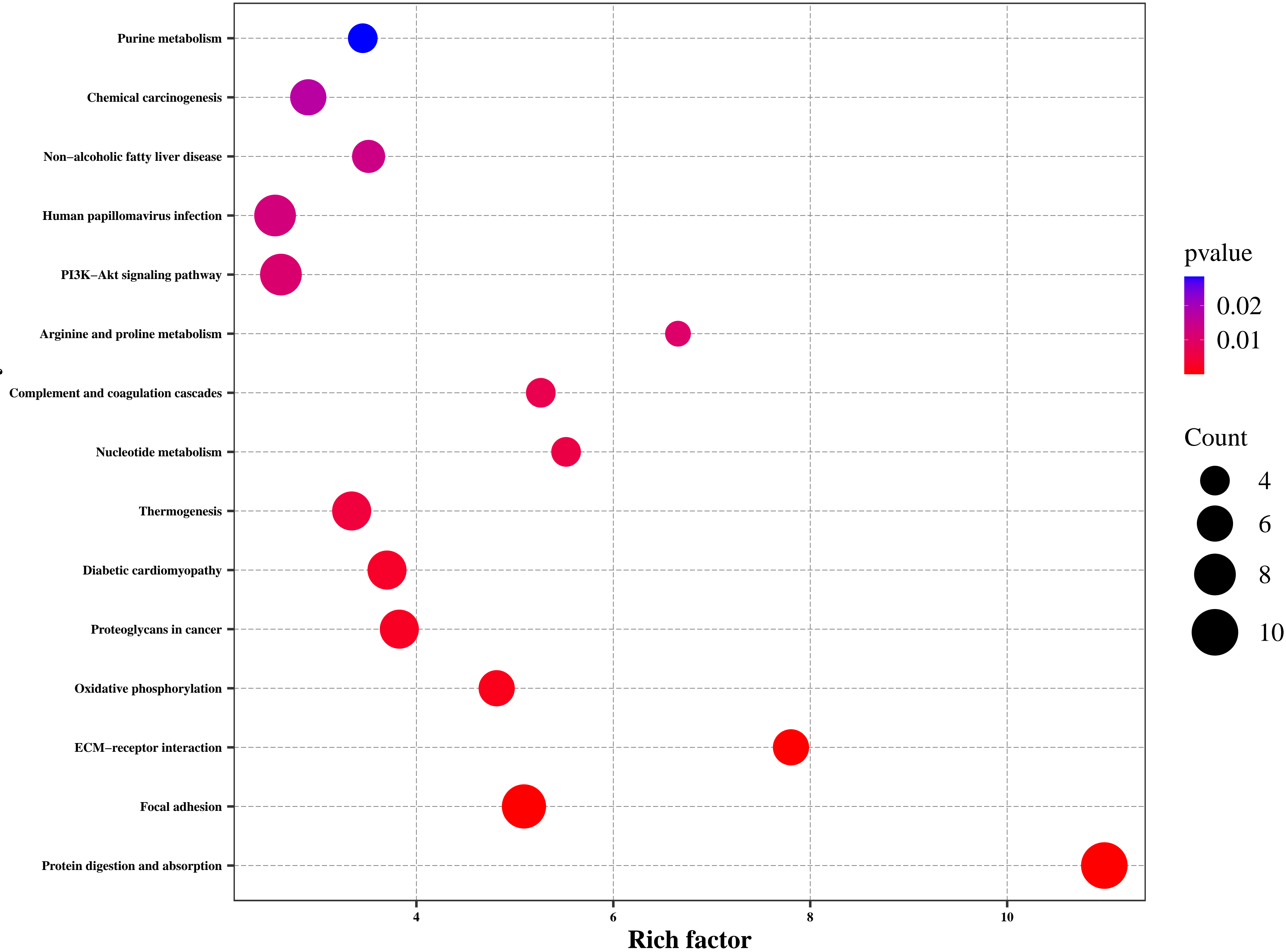

Supplement: S1 File — (ZIP) [file pone.0325562.s001.zip › S1_File/Proteomics analysis/KEGG Analysis/M_vs_C/KEGG Enrichment bubble.pdf]

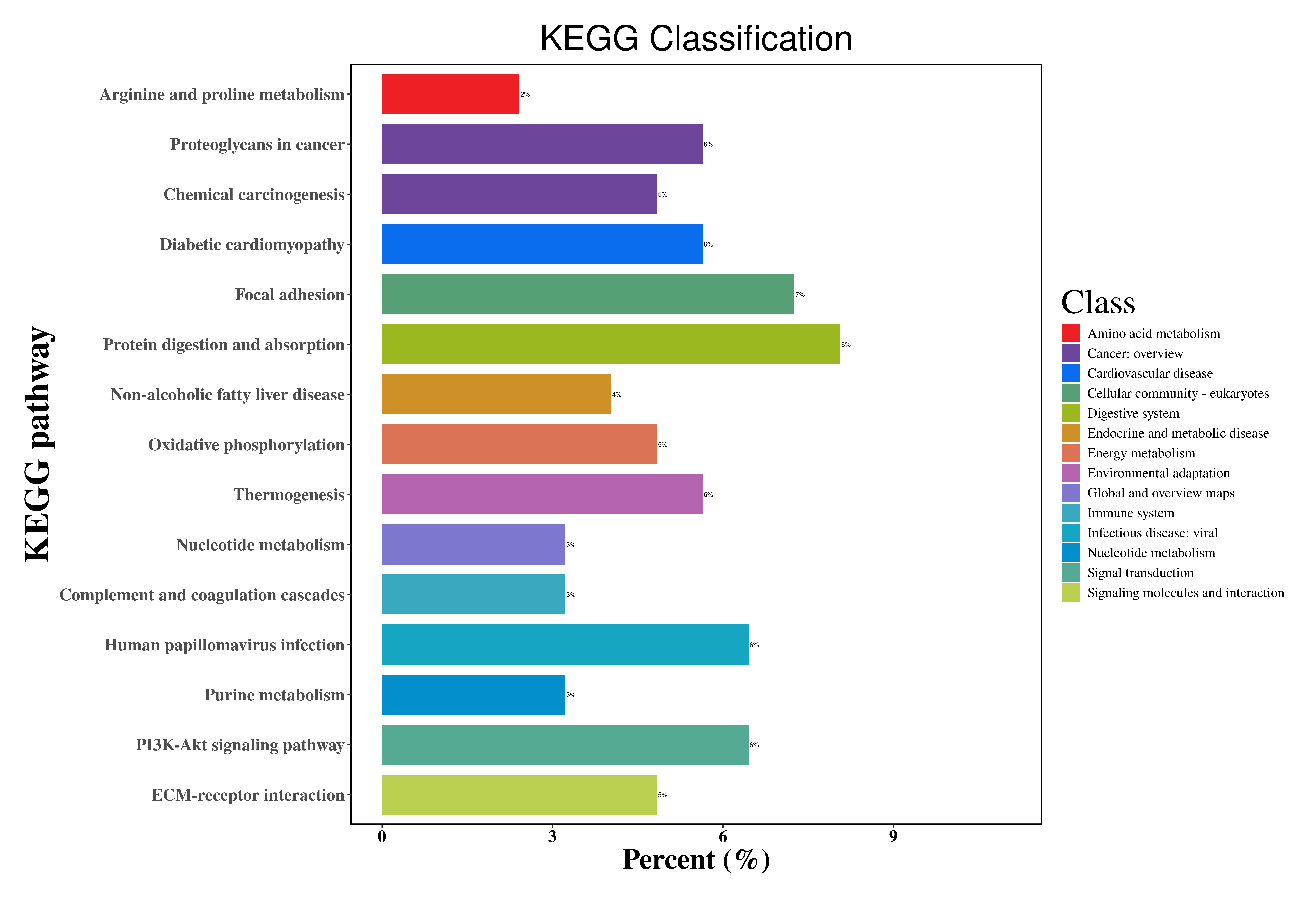

Supplement: S1 File — (ZIP) [file pone.0325562.s001.zip › S1_File/Proteomics analysis/KEGG Analysis/M_vs_C/KEGG Classification.png]
